# Discovery of tricyclic benzo[4,5]imidazo[2,1-*b*]thiazole-3-hydroxyindolin-2-one hybrids as selective MAO-B and cholinesterase inhibitors

**DOI:** 10.1039/d6ra05787j

**Published:** 2026-09-24

**Authors:** Wagdy M. Eldehna, Ashraf K. El-Damasy, Jaeyeop Lim, Mariam M. Hewala, Zainab M. Elsayed, Tarfah Al-Warhi, Anwar A. El-Hamaky, Man-Jeong Paik, Gyochang Keum, Mohamed R. Elnagar, Hoon Kim, Haytham O. Tawfik

**Affiliations:** a Department of Pharmaceutical Chemistry, Faculty of Pharmacy, Kafrelsheikh University P.O. Box 33516 Kafrelsheikh Egypt wagdy2000@gmail.com; b Department of Medicinal Chemistry, Faculty of Pharmacy, Mansoura University Mansoura 35516 Egypt ph_karem2000@man.edu.eg; c College of Pharmacy, Sunchon National University Suncheon 57922 Republic of Korea hoon@sunchon.ac.kr; d Scientific Research and Innovation Support Unit, Faculty of Pharmacy, Kafrelsheikh University Kafr El-Shaikh 33516 Egypt; e Department of Chemistry, College of Science, Princess Nourah Bint Abdulrahman University P.O. Box 84428 Riyadh 11671 Saudi Arabia; f Department of Pharmaceutical Chemistry, Faculty of Pharmacy, Tanta University Tanta 31527 Egypt; g Medicinal Materials Research Center, Biomedical Research Division, Korea Institute of Science and Technology (KIST) Hwarang-ro14gil 5, Seongbuk-gu Seoul 02792 Republic of Korea; h Department of Pharmacology and Toxicology, Faculty of Pharmacy, Al-Azhar University Cairo 11823 Egypt; i Department of Pharmacology, College of Pharmacy, The Islamic University Najaf 54001 Iraq; j Department of Pharmaceutical Chemistry, Faculty of Pharmacy, Alsalam University 31511 Tanta Egypt

## Abstract

A series of 13 novel 3-hydroxyindolin-2-one-benzo[4,5]imidazo[2,1-*b*]thiazole hybrids was designed, synthesized, and evaluated as potential anti-Alzheimer agents targeting monoamine oxidase B (MAO-B) and cholinesterases. The target compounds were obtained by condensing a benzo[4,5]imidazo[2,1-*b*]thiazole-based ketone with substituted and *N*-alkylated isatins and were structurally confirmed by ^1^H NMR, ^13^C NMR, and elemental analysis. All synthesized compounds were screened for their inhibitory activities against MAO-A and MAO-B enzymes. Most derivatives displayed weak MAO-A inhibition while exhibiting a marked preference for MAO-B inhibition. Among them, compounds 6c and 6b emerged as the most potent MAO-B inhibitors, with IC_50_ values of 0.90 ± 0.005 and 0.97 ± 0.010 µM, respectively. Structure–activity relationship studies revealed that substitution at the C-5 position of the isatin ring, particularly with halogen atoms, significantly enhanced MAO-B inhibitory activity, whereas *N*-alkylation generally reduced both potency and selectivity. Based on their superior MAO-B inhibitory profiles, compounds 6b and 6c were further evaluated against acetylcholinesterase (AChE) and butyrylcholinesterase (BuChE). Notably, compound 6c exhibited potent dual cholinesterase inhibition, showing AChE inhibitory activity comparable to that of donepezil and superior BuChE inhibition. Furthermore, SwissADME analysis indicated favorable drug-likeness properties for the most active compounds. Molecular docking studies demonstrated favorable binding interactions of the lead compounds within the active sites of MAO-B and AChE, while a 200 ns molecular dynamics simulation confirmed the stability of the MAO-B-6c complex. Collectively, these findings identify 6c as a promising multitarget lead compound combining selective MAO-B inhibition with potent cholinesterase inhibitory activity and highlight the 3-hydroxyindolin-2-one-benzo[4,5]imidazo[2,1-*b*]thiazole scaffold as a valuable platform for the development of novel therapeutic agents for Alzheimer's disease.

## Abstract 2

A series of 13 novel 3-hydroxyindolin-2-one-benzo[4,5]imidazo[2,1-*b*]thiazole hybrids was designed, synthesized, and evaluated as potential anti-Alzheimer agents targeting monoamine oxidase B (MAO-B) and cholinesterases.

## Graphical abstract

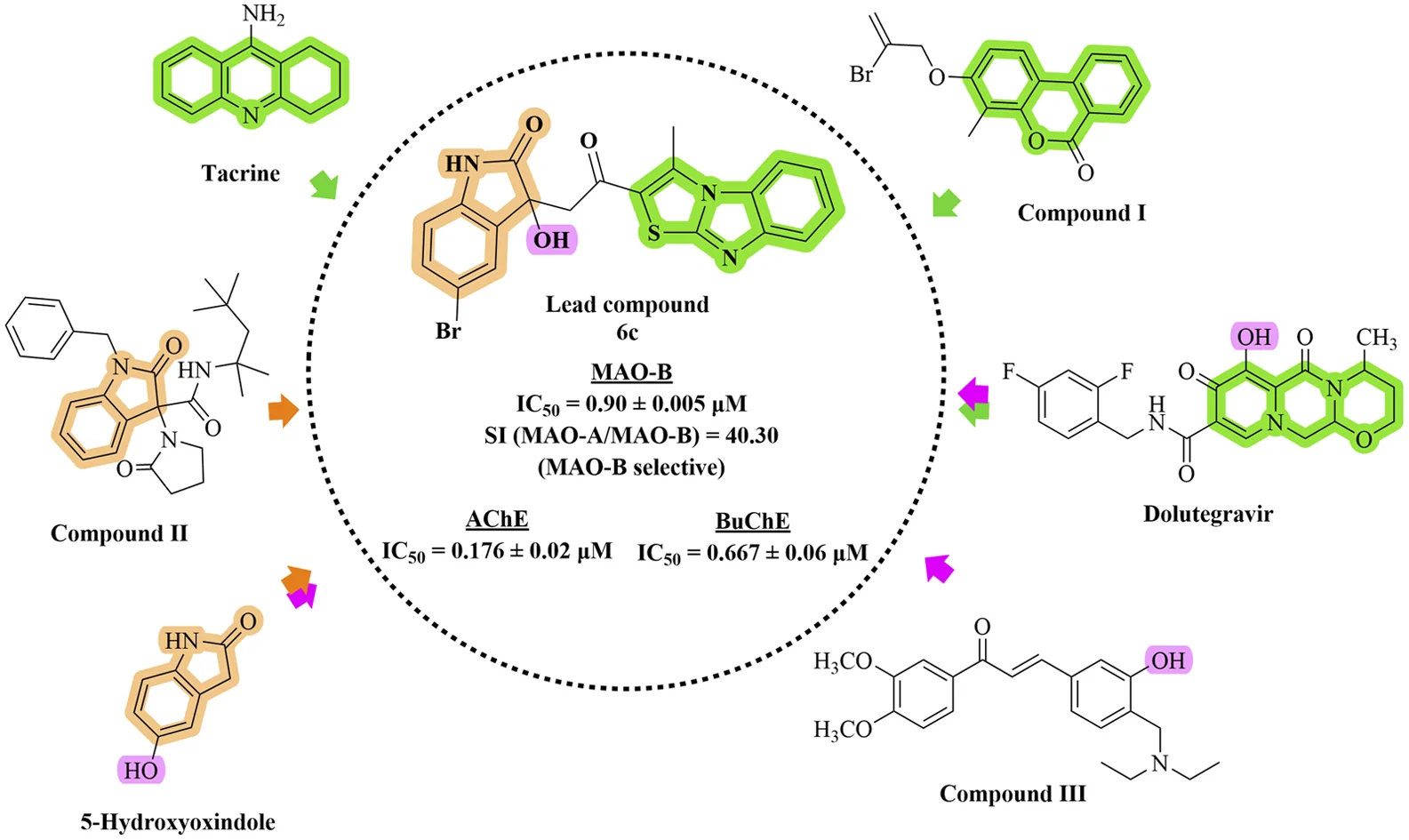

## Introduction

The progressive loss of neuronal structure and function, which eventually results in cognitive and motor deficits, is the hallmark of a diverse set of disorders known as neurodegenerative diseases (NDs).^1^ The prevalence of NDs has significantly increased due to the world's population's longer life expectancy, placing significant medical, social, and financial burdens on people all over the world.^2^ The most prevalent type of dementia among these conditions is Alzheimer's disease (AD), which continues to be a significant unresolved clinical problem.^3^ Even while our understanding of the molecular causes of neurodegeneration has advanced significantly, the treatments that are currently on the market primarily relieve symptoms rather than stopping the disease's progression.^4^ The limitations of single-target therapeutic approaches have been brought to light by the multifactorial nature of neurodegenerative disorders, which include oxidative stress, mitochondrial dysfunction, neuroinflammation, neurotransmitter imbalance, and neuronal loss.^5^ This has sparked increased interest in the development of multitarget-directed ligands (MTDLs), which simultaneously modulate multiple pathological pathways.^6^

Alzheimer's disease (AD), one of the most common neurodegenerative diseases, is particularly concerning because of its severe effects on cognitive abilities.^7,8^ Cholinergic dysfunction, oxidative stress, mitochondrial impairment, and neuroinflammation are only a few of the many interrelated pathways that make up the extremely complicated etiology of AD.^9^ Due to its involvement in the oxidative deamination of biogenic amines and the subsequent production of reactive oxygen species, monoamine oxidase-B (MAO-B) has garnered significant attention among the several therapeutic targets linked to AD.^10,11^ Notably, MAO-B expression and activity increase with age and in AD brains, leading to oxidative stress and neuronal damage.^12^ Concurrently, the cholinergic hypothesis, which holds that decreased acetylcholine levels cause progressive learning and memory impairment, remains one of the most generally recognized hypotheses of cognitive decline in AD.^13^ As a result, inhibiting MAO-B and cholinesterases has become a proven treatment approach for managing AD symptoms and reducing degenerative processes linked to neurodegeneration.^14^

A number of clinically licensed medications demonstrate the therapeutic value of MAO-B and cholinesterase inhibition. By modulating monoaminergic neurotransmission and reducing oxidative stress, selective MAO-B inhibitors such as safinamide, rasagiline, and selegiline have shown positive effects in neurodegenerative diseases.^15^ Similarly, cholinesterase inhibitors, such as galantamine, rivastigmine, and donepezil, continue to be among the standard therapies for AD-related cognitive impairments.^16–18^ However, there is increasing interest in multitarget-directed ligands that simultaneously control complementary pathogenic pathways, given the limited effectiveness of single-target therapies in addressing the complex nature of neurodegeneration.^19^ Dual MAO-B/cholinesterase inhibitors have become a viable therapeutic strategy in this regard, combining attenuation of MAO-B-mediated oxidative stress and neuronal damage with improvement of cholinergic neurotransmission (Fig. 1).^20^

**Fig. 1:**
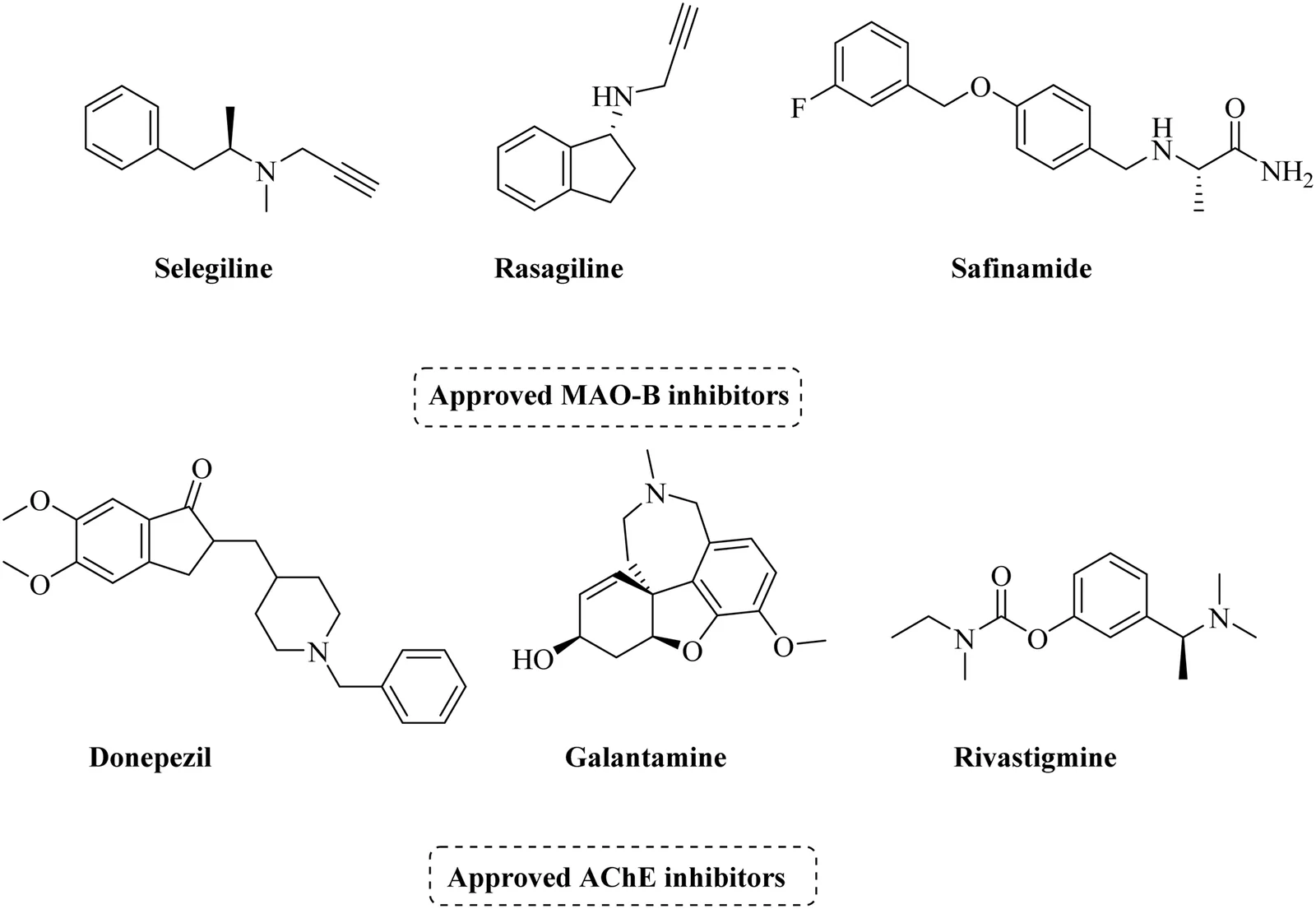
Clinically approved inhibitors targeting MAO-B and cholinesterases for the treatment of neurodegenerative disorders.

Because of their wide range of biological activity and structural diversity, fused heterocyclic systems have garnered significant interest in medicinal chemistry. Specifically, tricyclic heteroaromatic scaffolds have become preferred motifs in the development of enzyme inhibitors and neuroactive agents. Representative examples include the clinically approved acetylcholinesterase inhibitor tacrine^21^ and several tricyclic derivatives, such as compound I,^22^ which exhibit potent inhibitory activity against neurodegeneration-related targets. Moreover, the FDA-approved anti-HIV drug dolutegravir,^22^ which possesses a tricyclic heteroaromatic framework and a strategically positioned hydroxyl group, has recently attracted interest as a dual AChE/MAO-B inhibitor, highlighting the potential of combining structural rigidity with hydrogen-bonding functionalities in the development of multitarget neuroactive agents (Fig. 2).

**Fig. 2:**
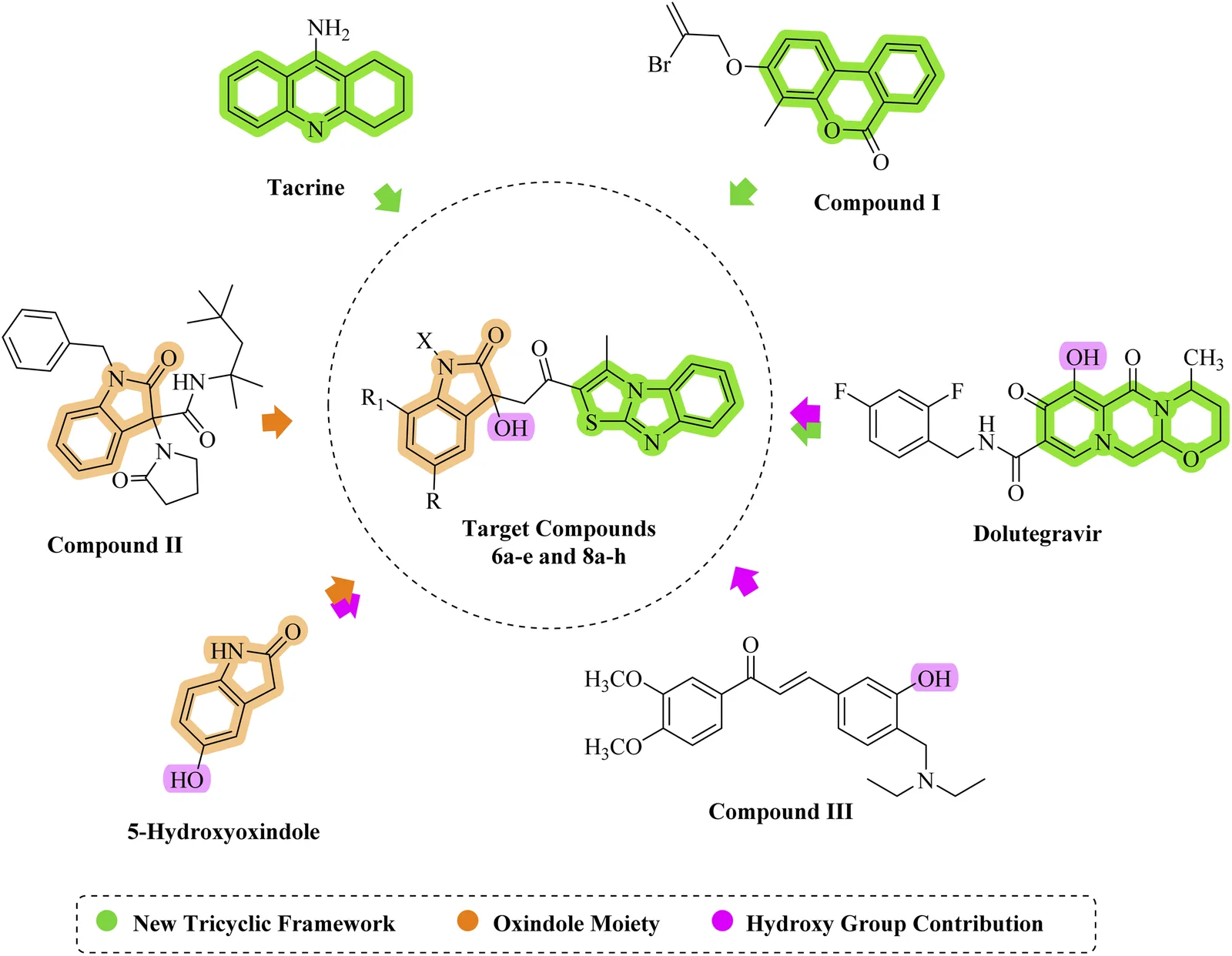
Rational design of tricyclic benzo[4,5]imidazo[2,1-*b*]thiazole-3-hydroxyindolin-2-one hybrids through integration of tricyclic heteroaromatic, oxindole, and hydroxyl pharmacophoric features.

Another privileged pharmacophore extensively explored in neurodegenerative drug discovery is the oxindole scaffold. Oxindole-containing molecules have demonstrated a wide range of neuroprotective and cholinesterase inhibitory activities. For example, 5-hydroxyoxindole,^23^ which incorporates both oxindole and hydroxyl functionalities, exhibited potent BuChE inhibitory activity, whereas compound II,^24^ bearing the oxindole nucleus, displayed promising activity against neurodegeneration-associated targets. In addition, hydroxyl-containing compounds such as compound III^22^ exhibited enhanced enzyme inhibitory activity, underscoring the importance of hydroxyl groups as hydrogen-bond donors/acceptors, which can improve enzyme recognition and binding affinity.

Within this structural class, isatin (1*H*-indole-2,3-dione) has attracted considerable interest owing to its broad spectrum of biological activities, including anticancer, antimicrobial, anti-inflammatory, antiviral, and neuroprotective effects.^25–29^ Of particular relevance to neurodegenerative disorders, numerous isatin derivatives have demonstrated inhibitory activity against key therapeutic targets, notably monoamine oxidases and cholinesterases. This broad pharmacological profile, together with its structural versatility, has established isatin as an attractive scaffold for the development of multitarget-directed ligands.

Motivated by these findings and continuing our efforts to create multitarget-directed ligands for neurodegenerative diseases, we hypothesized that combining three complementary structural elements, a hydroxyl functionality, an oxindole pharmacophore, and a rigid tricyclic heteroaromatic scaffold, could produce new chemotypes with enhanced inhibitory activity toward MAO-B and cholinesterases. As a result, thirteen new tricyclic benzo[4,5]imidazo[2,1-*b*]thiazole-3-hydroxyindolin-2-one hybrids were designed. The goal of the developed compounds was to combine the neuroactive qualities of oxindole and the hydrogen-bonding capacity of the hydroxyl group with the structural rigidity and hydrophobic/aromatic interactions of tricyclic systems. Additionally, the effects of *N*-alkylation and C-5/C-7 substitution on enzyme selectivity and inhibition were thoroughly examined. To elucidate the observed biological activities and assess the drug-likeness of the identified lead compounds, molecular docking, molecular dynamics simulations, and *in silico* ADME studies were performed. The synthesized compounds were evaluated against MAO-A and MAO-B, and the most active derivatives were further evaluated against AChE and BuChE.

## Results and discussion

### Chemistry

Schemes 1 and 2 describe the synthetic pathway used to prepare the target agents. First, *o*-phenylenediamine and carbon disulfide were reacted in an ethanolic potassium hydroxide solution to create the crucial precursor, 1*H*-benzo[*d*]imidazole-2-thiol (2), in accordance with the stated approach. Compound 2 was then reacted with 3-chloropentane-2,4-dione in alcoholic potassium hydroxide to produce 3-((1*H*-benzo[*d*]imidazol-2-yl)thio)pentane-2,4-dione (3). Compound 3's cyclization in acetic anhydride with a catalytic quantity of pyridine produced 1-(3-methylbenzo[4,5]imidazo[2,1-*b*]thiazol-2-yl)ethan-1-one (4).^30,31^ Crucially, compound 4 served as a synthetic intermediate in the synthesis of the intended target derivatives. Key intermediate 4 was treated with several isatins (5a–e) in ethanol containing catalytic diethylamine at 20 °C, as shown in Scheme 1, to produce the target compounds 6a–e*via* Knoevenagel-type condensation in good yields.

**Scheme 1:**
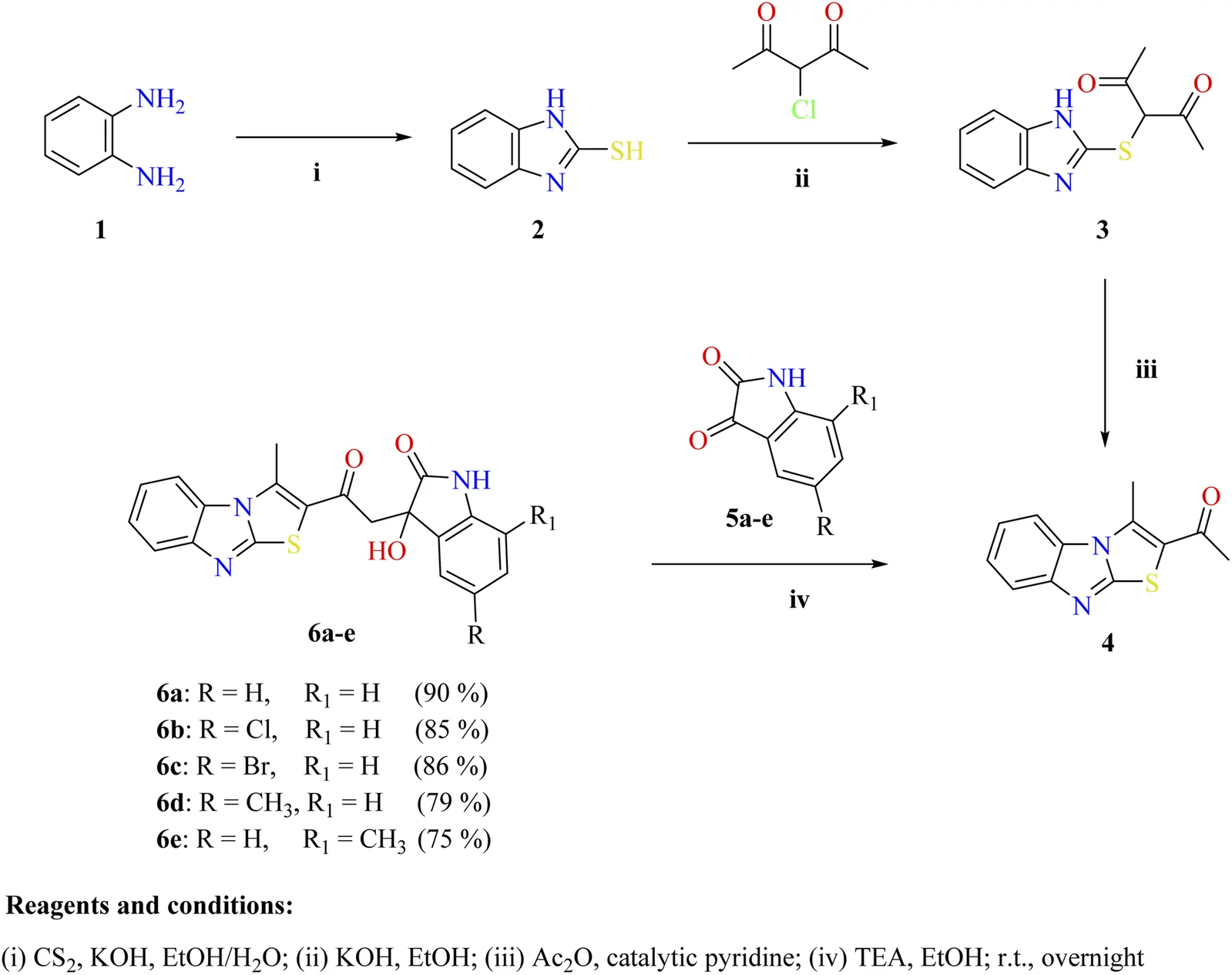
The synthetic route of 3-hydroxyindolin-2-one-benzo[4,5]imidazo[2,1-*b*]thiazole hybrids 6a–e.

**Scheme 2:**
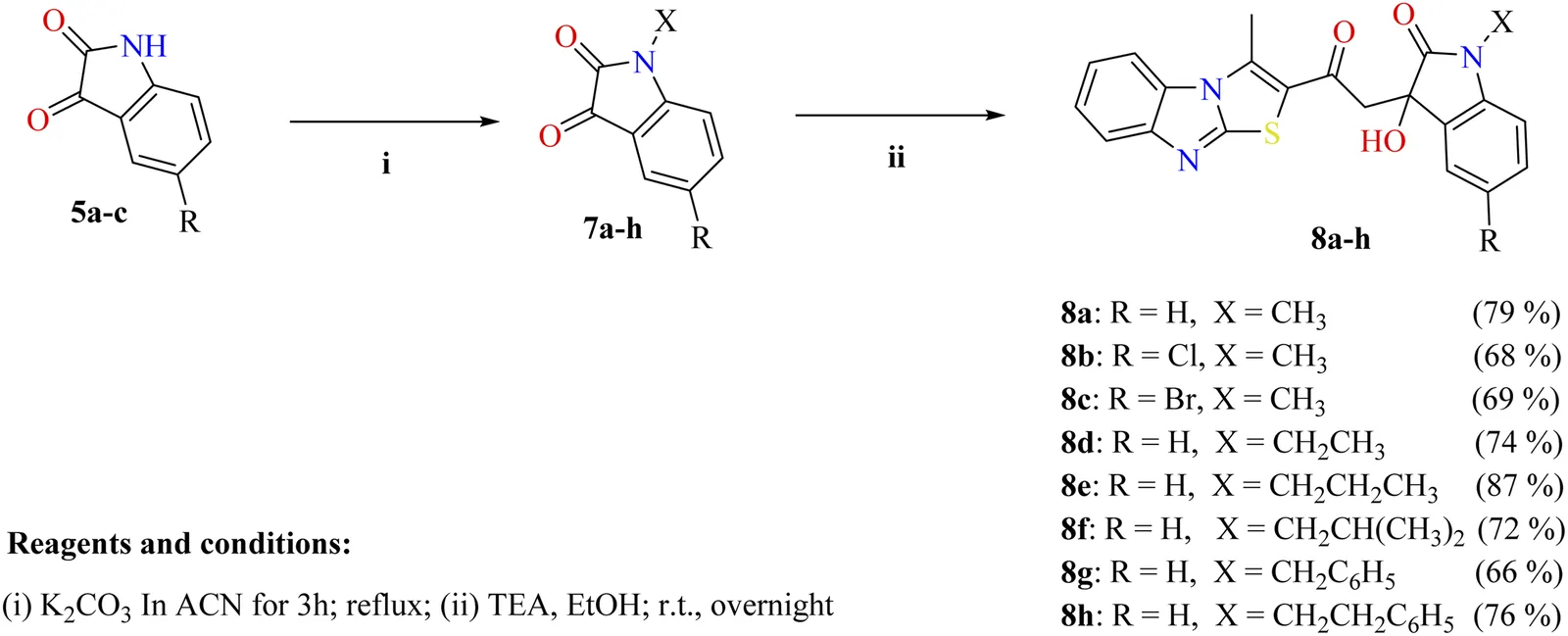
The synthetic route of 3-hydroxyindolin-2-one-benzo[4,5]imidazo[2,1-*b*]thiazole hybrids 8a–h.

The corresponding *N*-alkylated isatin derivatives (7a–h) were created by alkylating the isatin nitrogen using a variety of alkylating agents, including methyl bromide, ethyl bromide, propyl bromide, isobutyl bromide, benzyl bromide, and 2-phenylethyl bromide, in order to examine the impact of *N*-alkylation on the biological activity (Scheme 2). The corresponding *N*-alkylated target derivatives 8a–h were then obtained by subjecting compounds 7a–h to the identical condensation conditions with intermediate 4 in ethanol containing diethylamine at 20 °C.

Structural characterization of compounds 6a–e and 8a–h was accomplished using ^1^H and ^13^C NMR spectroscopy, together with elemental analysis, which yielded values in satisfactory agreement with the calculated compositions.

The consumption of the first ketone moiety during the reaction was confirmed by the full disappearance of the distinctive singlet in the ^1^H NMR spectra that corresponded to the three protons of the acetyl methyl group of intermediate 4 around 2.1 ppm. At roughly *δ* 3.60 and 4.00 ppm, a new methylene signal emerged simultaneously as two separate doublets that each integrated for one proton. These two protons are nonequivalent because they are diastereotopic, arising from the formation of a nearby stereogenic center during cyclization. As a result, instead of producing a single methylene signal, each proton experiences a distinct magnetic environment and interacts geminally with the other proton, producing two doublets. The development of the desired hydroxy-containing scaffold was further supported by the observation of a distinctive hydroxyl proton signal at *δ* 6.30 ppm.

The ^13^C NMR spectra provided additional corroboration, demonstrating the elimination of the acetyl methyl carbon resonance that was first detected in intermediate 4 at about *δ* 25 ppm. Additionally, the ketonic carbonyl carbon signal of the isatin precursor vanished, consistent with its involvement in the cyclization. Concurrently, the formation of the newly formed CH_2_ group in the final products was supported by the appearance of a new methylene carbon resonance at approximately *δ* 48 ppm. The suggested structures were completely compatible with the remaining carbon and proton resonances.

The molecular formulas assigned to compounds 6a–e and 8a–h were supported by their corresponding molecular ion signals and elemental analysis data, with the latter showing deviations within the acceptable ±0.4% range.

### Biological section

#### MAO inhibitory activity and isoform selectivity

The synthesized 3-hydroxyindolin-2-one-benzo[4,5]imidazo[2,1-*b*]thiazole derivatives exhibited a pronounced preference for MAO-B over MAO-A. Most compounds were essentially inactive against MAO-A, displaying IC_50_ values exceeding 40 µM, except for compounds 6b, 6c, and 6d, which showed moderate MAO-A inhibition with IC_50_ values of 16.62 ± 0.55, 36.27 ± 2.08, and 37.80 ± 2.92 µM, respectively. The marked difference in the inhibitory potencies of MAO-A and MAO-B highlights the designed scaffold's ability to preferentially recognize the MAO-B active site.

In terms of MAO-B inhibition, the unalkylated isatin derivatives (6a–e) generally displayed superior activity compared with their corresponding *N*-alkylated counterparts (8a–h), suggesting that preservation of the NH functionality is favorable for enzyme binding. The unsubstituted analogue 6a inhibited MAO-B with an IC_50_ value of 3.89 ± 0.40 µM, while structural modification at the isatin ring significantly enhanced potency. Introduction of substituents at C-5 proved particularly beneficial, indicating that this position plays a pivotal role in modulating MAO-B inhibitory activity. For example, incorporation of a bromine atom afforded 6c, the most potent member of the series (IC_50_ = 0.90 ± 0.005 µM), closely followed by the chloro analogue 6b (IC_50_ = 0.97 ± 0.010 µM). The methyl-substituted derivative 6d remained highly active (IC_50_ = 1.83 ± 0.17 µM) but was less potent than the corresponding halogenated analogues. Accordingly, the activity trend for C-5 substitution was established as: Br (6c) > Cl (6b) > CH_3_ (6d) > H (6a).

Interestingly, comparison of compounds 6d and 6e revealed that the position of substitution on the isatin nucleus markedly influences activity. Relocation of the methyl group from C-5 (6d) to C-7 (6e) caused a substantial loss of potency, with the MAO-B IC_50_ increasing from 1.83 ± 0.17 µM to 4.83 ± 0.18 µM, indicating that substitution at C-5 is considerably more advantageous than at C-7 for MAO-B inhibition (Table 1).

**Table 1:** IC_50_ (µM) values of the newly synthesized compounds against hMAO isoforms

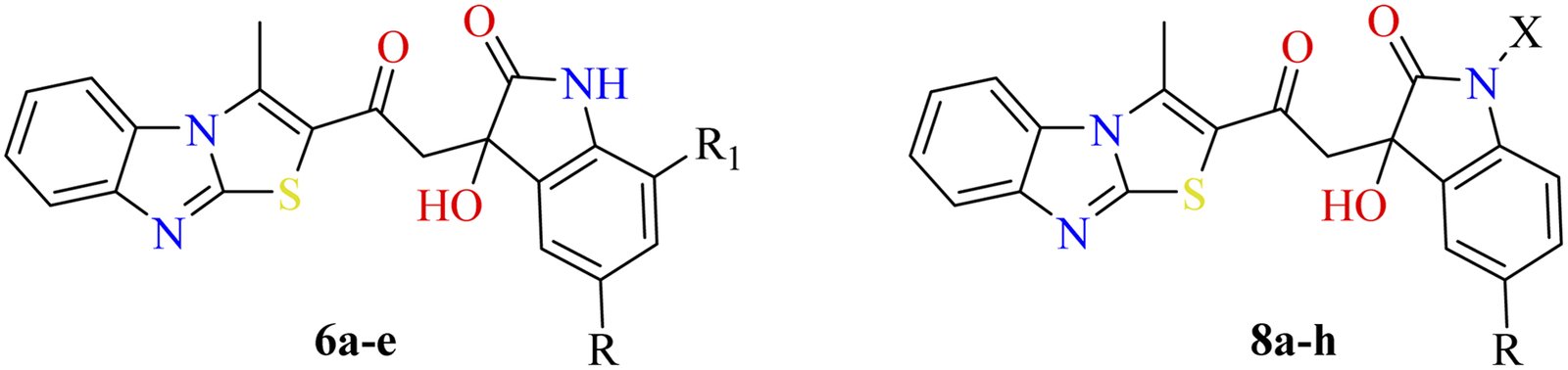							
Code	Compounds			IC_50_ (µM)^a^		SI^b^	Selectivity
	Variants						
	R	R_1_	X	MAO-A	MAO-B		
6a	H	H	—	>40	3.89 ± 0.40	>10.28	MAO-B
6b	Cl	H	—	16.62 ± 0.55	0.97 ± 0.010	17.13	MAO-B
6c	Br	H	—	36.27 ± 2.08	0.90 ± 0.005	40.30	MAO-B
6d	CH_3_	H	—	37.80 ± 2.92	1.83 ± 0.17	20.66	MAO-B
6e	H	CH_3_	—	>40	4.83 ± 0.18	>8.28	MAO-B
8a	H	—	CH_3_	>40	6.69 ± 0.37	>5.98	MAO-B
8b	Cl	—	CH_3_	>40	7.16 ± 1.09	>5.59	MAO-B
8c	Br	—	CH_3_	>40	4.27 ± 0.18	>9.37	MAO-B
8d	H	—	CH_2_CH_3_	>40	8.31 ± 0.26	>4.81	MAO-B
8e	H	—	CH_2_CH_2_CH_3_	>40	8.16 ± 0.26	>4.90	MAO-B
8f	H	—	CH_2_CH(CH_3_)_2_	>40	8.50 ± 0.20	>4.71	MAO-B
8g	H	—	CH_2_C_6_H_5_	>40	9.27 ± 0.29	4.31	MAO-B
8h	H	—	CH_2_CH_2_C_6_H_5_	>40	4.25 ± 0.16	>9.41	MAO-B
Toloxatone				1.510 ± 0.28	>40	—	MAO-A
Clorgyline				0.018 ± 0.00005	4.644 ± 0.331	—	MAO-A
Safinamide				>40	0.033 ± 0.0006	—	MAO-B
Pargyline				2.390 ± 0.14	0.126 ± 0.0049	—	MAO-B

a Measurements were performed in a minimum of three independent replicates to confirm reproducibility.

b SI denotes the selectivity index, calculated as IC_50_ (MAO-A)/IC_50_ (MAO-B).

The detrimental effect of *N*-alkylation was further confirmed by direct comparison of matched pairs. Thus, compound 6a was approximately 1.7-fold more potent than 8a (3.89 *vs.* 6.69 µM), while much larger reductions in activity were observed for 6b*versus*8b (0.97 *vs.* 7.16 µM) and 6c*versus*8c (0.90 *vs.* 4.27 µM). These findings suggest that the free isatin NH group may favorably contribute to enzyme recognition and binding, whereas *N*-alkylation compromises these interactions.

Within the *N*-alkylated series lacking C-5/C-7 substitution, the nature of the *N*-substituent exerted a measurable influence on activity. The phenethyl derivative 8h emerged as the most potent analogue (IC_50_ = 4.25 ± 0.16 µM), followed by 8a, 8e, 8d, 8f, and 8g, with IC_50_ values of 6.69 ± 0.37, 8.16 ± 0.26, 8.31 ± 0.26, 8.50 ± 0.20, and 9.27 ± 0.29 µM, respectively. Although none of the *N*-alkylated analogues surpassed the corresponding NH-containing derivatives, the improved activity of 8h relative to the remaining members suggests that elongation of the *N*-substituent with a terminal phenyl ring is more favorable than simple aliphatic substitution (Fig. 3).

**Fig. 3:**
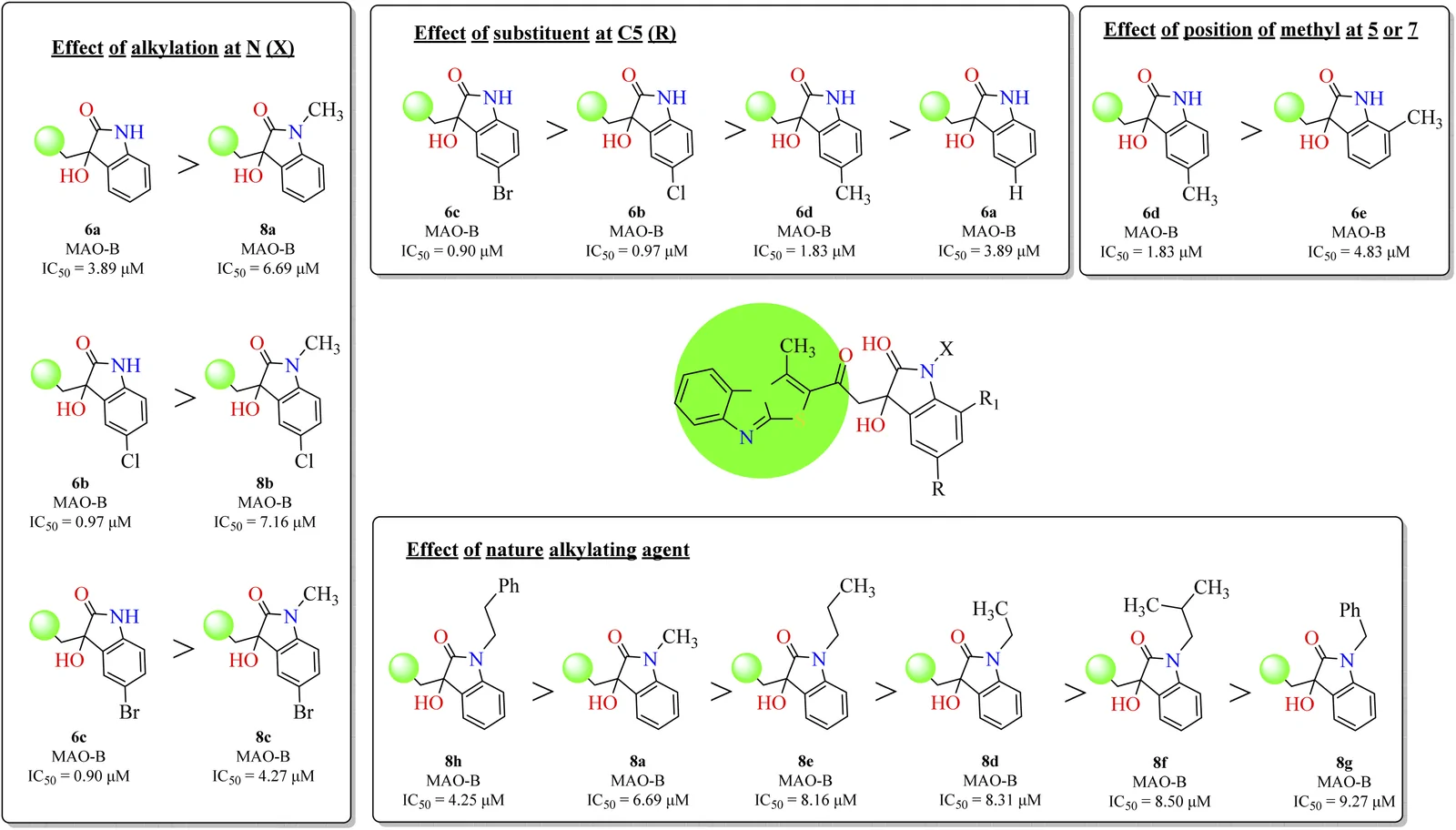
Structure–activity relationship of tricyclic benzo[4,5]imidazo[2,1-*b*]thiazole-3-hydroxyindolin-2-one hybrids (6a–e and 8a–h) for MAO-B inhibition.

#### Selectivity index (SI)

Regarding selectivity, all synthesized compounds preferentially inhibited MAO-B, exhibiting selectivity indices (SI) ranging from 4.31 to 40.30. The highest selectivity was observed for the brominated analogue 6c (SI = 40.30), followed by 6d (SI = 20.66) and 6b (SI = 17.13), whereas the lowest selectivity was recorded for 8g (SI = 4.31). Notably, SI values exceeding 10 were almost exclusively associated with the unalkylated isatin derivatives, namely 6a (10.28), 6b (17.13), 6c (40.30), and 6d (20.66). In contrast, introduction of either C-7 substitution (6e, SI = 8.28) or *N*-alkyl groups (8a–h, SI = 4.31–9.41) generally reduced MAO-B selectivity. These findings indicate that maintaining a free isatin NH, together with appropriate substitution at C-5, is beneficial not only for enhancing MAO-B inhibitory potency but also for maximizing enzyme selectivity.

Interestingly, the unalkylated analogues (6a–e), bearing a free lactam NH within the 3-hydroxyindolin-2-one moiety, generally exhibited superior MAO-B inhibitory activity compared with their corresponding *N*-alkylated derivatives (8a–h). For instance, compounds 6a, 6b, and 6c showed improved MAO-B inhibition compared with their *N*-methyl counterparts 8a, 8b, and 8c, respectively, suggesting that the presence of a free amide NH may favor enzyme recognition and binding. This observation is in agreement with previous reports highlighting the prevalence of amide-containing pharmacophores among clinically used and experimentally validated MAO inhibitors, including isatin, safinamide, moclobemide, and lazabemide.^32^ Notably, most of these compounds possess a free amide NH group, which has been proposed to contribute favorably to molecular recognition through hydrogen-bonding interactions within the enzyme binding site. Therefore, the enhanced activity of the present unalkylated analogues may, at least in part, be attributed to the retention of this important structural feature.

#### AChE and BuChE inhibitory activity

Based on their potent MAO-B inhibitory activity, compounds 6b and 6c were initially selected for further evaluation against AChE and BuChE. To provide broader insight into the cholinesterase inhibitory potential of this scaffold and establish a preliminary structure–activity relationship, the structurally related analogues 6d and 8c were additionally evaluated (Table 2).

**Table 2:** IC_50_ values (µM) and selectivity indices (SI) for the inhibition of AChE and BuChE by compounds 6b, 6c, 6d and 8c

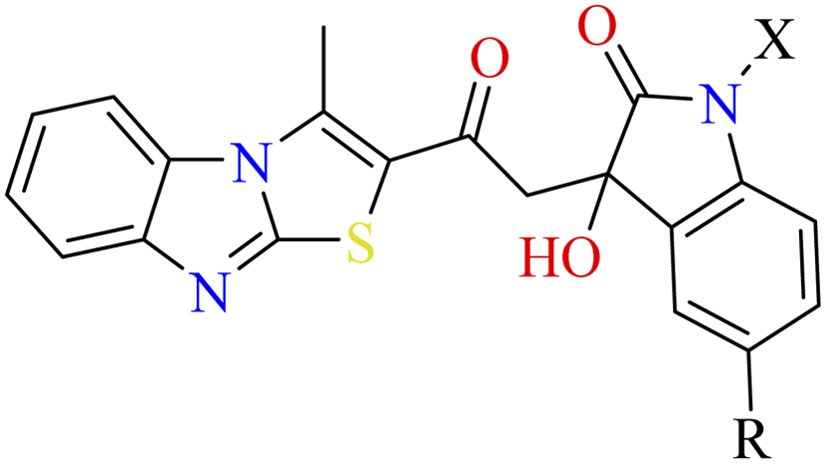				
Compounds			IC_50_ ± SD (µM)^a^	
Code	Variant			
	R	X	AChE	BuChE
6b	Cl	H	0.725 ± 0.05	3.029 ± 0.19
6c	Br	H	0.176 ± 0.02	0.667 ± 0.06
6d	CH_3_	H	2.714 ± 0.05	7.113 ± 0.35
8c	Br	CH_3_	0.539 ± 0.04	1.458 ± 0.05
Donepezil			0.098 ± 0.001	1.933 ± 0.06

a IC_50_ values are reported as the mean ± SD of five independent experiments, after excluding the highest and lowest values.

Among the tested derivatives, 6c exhibited the most potent cholinesterase inhibitory profile, with IC_50_ values of 0.176 ± 0.02 µM and 0.667 ± 0.06 µM against AChE and BuChE, respectively. Compound 6b showed lower inhibitory activity, with corresponding IC_50_ values of 0.725 ± 0.05 µM and 3.029 ± 0.19 µM, whereas the methyl-substituted analogue 6d was considerably less active against both enzymes (IC_50_ = 2.714 ± 0.05 µM for AChE and 7.113 ± 0.35 µM for BuChE). These results indicate that replacement of the substituent in this series follows the potency trend Br > Cl > CH_3_, suggesting a favorable contribution of the bromo substituent to cholinesterase inhibition.

Interestingly, compound 8c, which retains the bromo substituent but bears an additional methyl group at the X position, maintained appreciable inhibitory activity against both AChE and BuChE, with IC_50_ values of 0.539 ± 0.04 µM and 1.458 ± 0.05 µM, respectively. Nevertheless, 6c remained approximately 3.1-fold and 2.2-fold more potent than 8c against AChE and BuChE, respectively, suggesting that the unsubstituted X position is more favorable for cholinesterase inhibition.

Compared with donepezil, 6c was only approximately 1.8-fold less potent against AChE (donepezil IC_50_ = 0.098 ± 0.001 µM), while showing approximately 2.9-fold greater potency against BuChE (donepezil IC_50_ = 1.933 ± 0.06 µM). Collectively, the expanded cholinesterase evaluation supports 6c as the most balanced inhibitor within the tested subset, combining sub-micromolar inhibition of MAO-B, AChE, and BuChE, and further supports its potential as a promising multitarget lead for subsequent optimization.

### *In silico* study

#### Theoretical prediction of the ADME properties

SwissADME^33–35^ was employed to estimate the physicochemical and drug-likeness profiles of the leading MAO-B inhibitors 6b and 6c (Table 3). Their predicted molecular weights were 411.04 and 454.99 Da, respectively, with both values satisfying the molecular-weight criterion of Lipinski's rule of five. Additionally, the two compounds exhibited moderate lipophilicity (log *P* = 3.085–3.113) and identical topological polar surface area (tPSA = 83.70 Å^2^), suggesting a balanced polarity profile that may support adequate membrane permeability. Furthermore, compounds 6b and 6c possessed a limited number of rotatable bonds (nrotb = 3) and an acceptable hydrogen-bonding capacity (nON = 6 and nOHNH = 2), indicating suitable molecular flexibility and potential for intermolecular interactions. Importantly, neither compound triggered any PAINS alerts, suggesting a low probability of assay interference or nonspecific biological activity. Collectively, the predicted parameters supported acceptable drug-like characteristics for both compounds. On the basis of its stronger combined activity toward MAO-B, AChE, and BuChE, compound 6c was subsequently selected for detailed molecular docking and molecular dynamics analyses.

**Table 3:** Predicted drug-likeness profiles of compounds 6b and 6c relative to donepezil

Compound	*M*_W_ a	nrotb^b^	nON^c^	nOHNH^d^	tPSA^e^	log *P*^f^	PAINS alerts
6b	411.04	3	6	2	83.70	3.085	0
6c	454.99	3	6	2	83.70	3.113	0
Donepezil	379.21	6	4	0	38.77	3.245	0

a *M*_W_: molecular weight.

b nrotb: rotatable bond count.

c nON: hydrogen-bond acceptor count.

d nOHNH: hydrogen-bond donor count.

e tPSA: topological polar surface area.

f log *P*: octanol–water partition coefficient.

#### Molecular docking

The crystal structure of human MAO-B bound to safinamide (PDB ID: 2V5Z)^36^ was adopted for docking compound 6c. Validation by redocking the native ligand yielded an RMSD of 0.73 Å relative to its crystallographic pose, supporting the suitability of the docking protocol (Fig. 4).

**Fig. 4:**
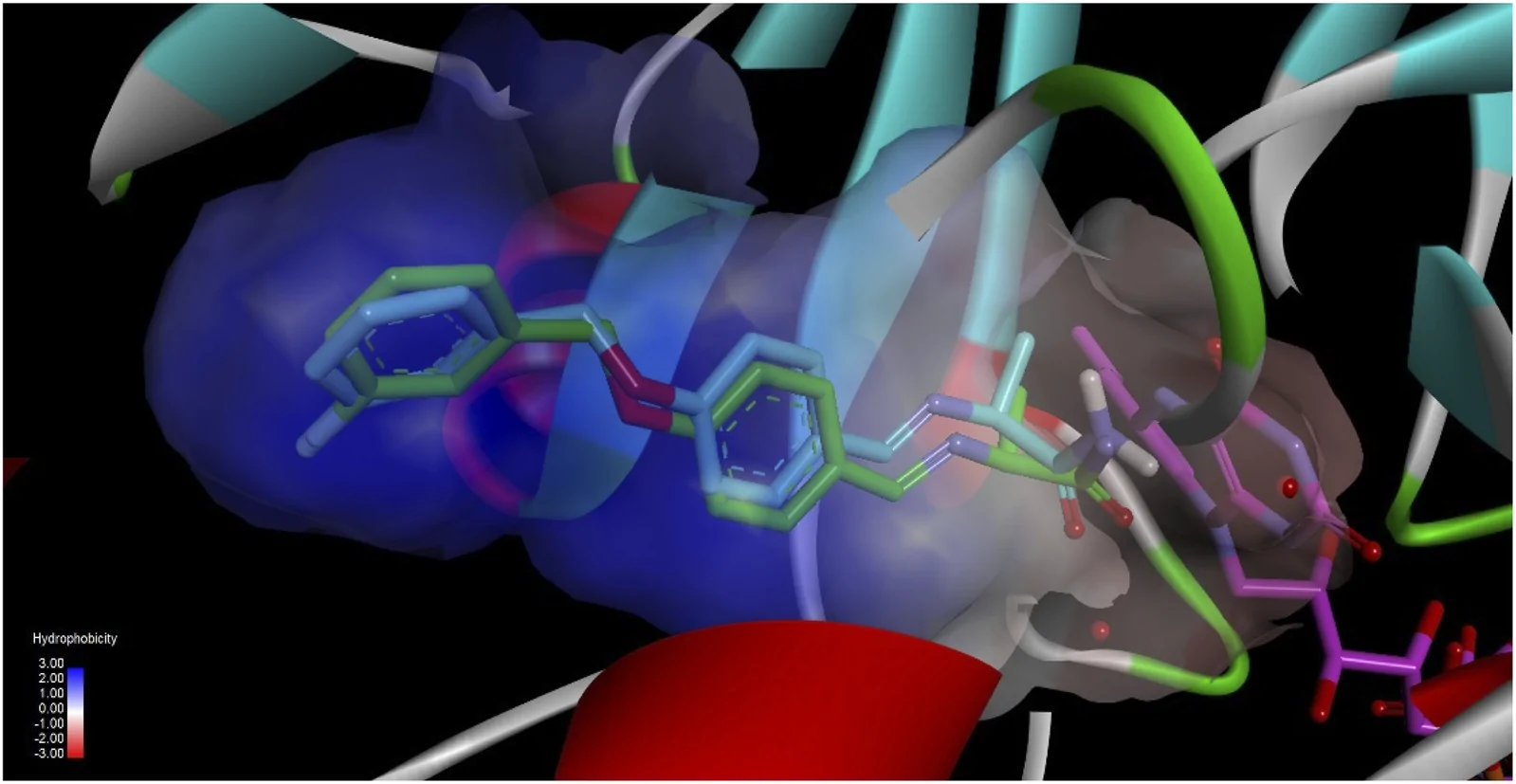
Redocking validation of the docking protocol in MAO-B. The co-crystallized safinamide, shown in green, is overlaid with its redocked pose, shown in cyan, within the MAO-B active site, shown as a surface, with the FAD cofactor in magenta. The tested compound is not shown in this figure.

Docking of compound 6c within the bipartite MAO-B binding cavity afforded a score of −9.99 kcal mol^−1^ (Fig. 5). The ligand spanned the active site and established π-alkyl contacts with the gating residues Ile199 and Tyr326. Its binding pose was further stabilized by conventional hydrogen bonds involving Leu171 and the aromatic-cage residue Tyr435, together with carbon–hydrogen bond interactions with Phe168 and Cys172. Also, several hydrophobic interactions were observed with Tyr60, Leu164, Ile316, Leu328, Met341, and Phe343. Moreover, the sulfur-containing ring of compound 6c contributed to π-sulfur interactions with Phe168 and Cys172. Finally, a π–π stacking interaction with the aromatic cage residue Tyr398 further stabilized the structure within the MAO-B active site (Fig. 5).

**Fig. 5:**
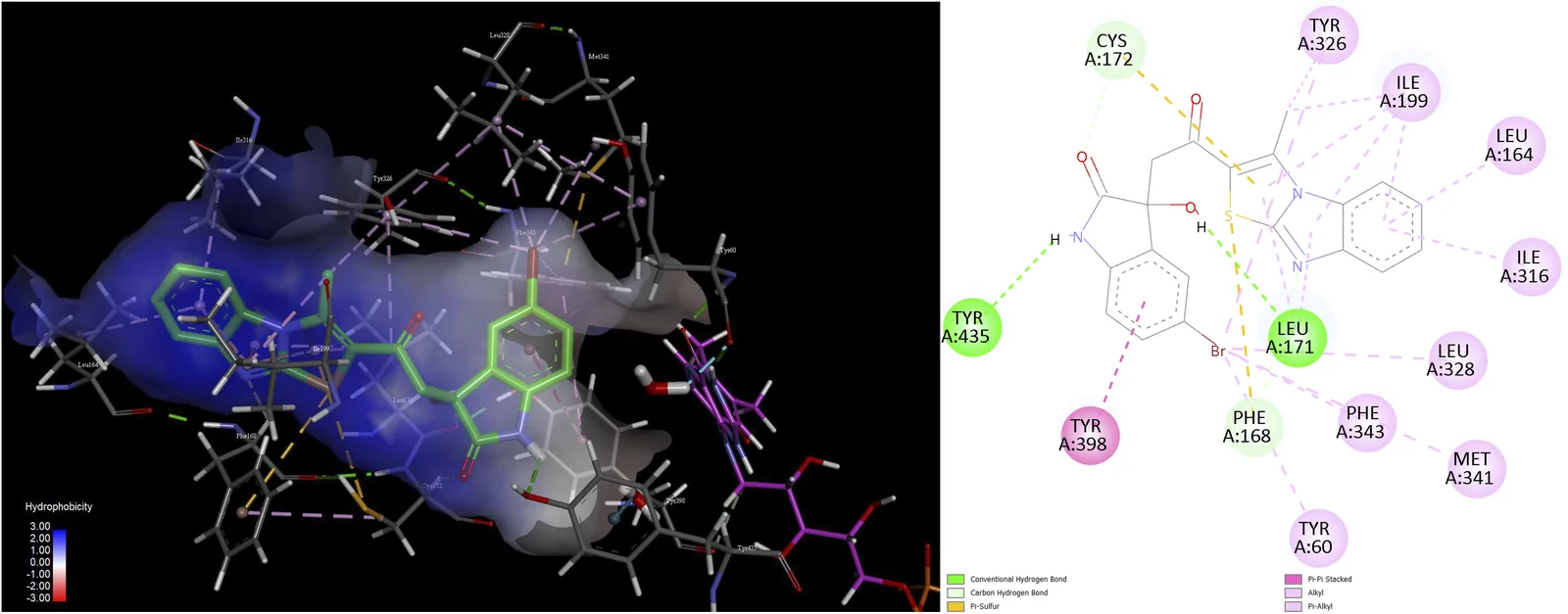
Three-dimensional (left) and two-dimensional (right) representations of the binding interactions of compound 6c within the MAO-B active site.

For the cholinesterase target, docking studies were conducted using the crystal structure of human acetylcholinesterase (hAChE) in complex with donepezil (PDB ID: 4EY7).^37^ Redocking of the native ligand reproduced its crystallographic binding pose with an RMSD of 0.55 Å, supporting the validity of the adopted docking procedure (Fig. 6).

**Fig. 6:**
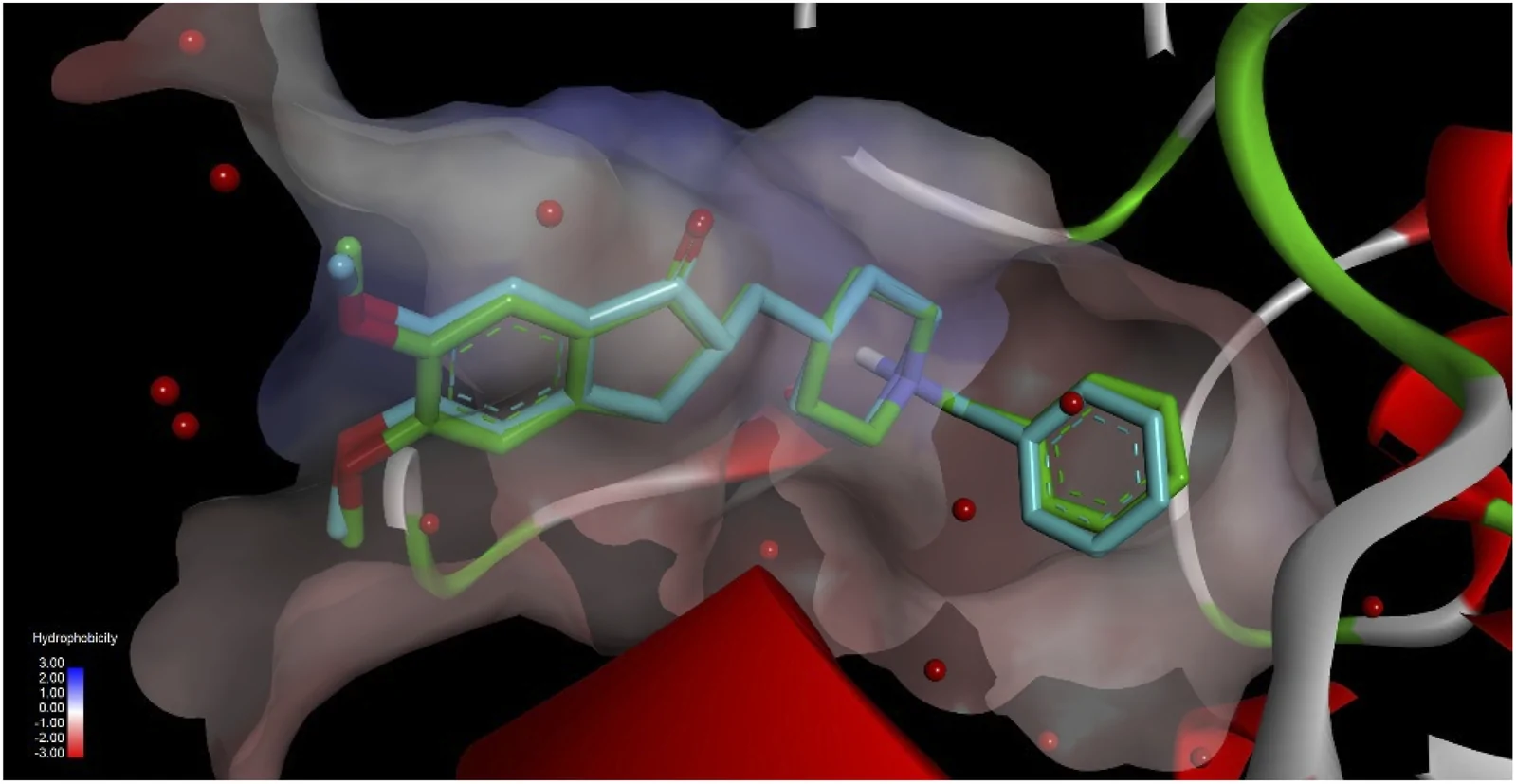
Redocking validation of the docking protocol in hAChE. The co-crystallized donepezil, shown in green, is overlaid with its redocked pose, shown in cyan, within the hAChE binding pocket, shown as a surface. The tested compound is not shown in this figure.

Docking analysis suggested that compound 6c adopted a dual-site binding orientation within the hAChE gorge, resembling the binding pattern of donepezil and simultaneously engaging the catalytic and peripheral anionic regions (Fig. 7). At the catalytic end, the bromo-dihydroindol-2-one moiety contacted Trp86, whereas the tricyclic portion extended toward the peripheral region and established π–π stacking with Trp286. Further aromatic contacts involved Phe297, Tyr337, and Tyr341, accompanied by π-alkyl interactions with Tyr124 and Val294. The bromine substituent formed a halogen bond with His447, while Val294 additionally participated in a carbon–hydrogen bond. The sulfur atom contributed π-sulfur contacts with Phe297 and Phe338. Moreover, the carbonyl group of the bromo-dihydroindol-2-one moiety engaged in a water-bridged hydrogen-bonding network involving HOH954, Asp74, and Tyr124 (Fig. 7). Together with the docking score of −10.40 kcal mol^−1^, this interaction pattern supports the ability of compound 6c to span both regions of the hAChE binding gorge.

**Fig. 7:**
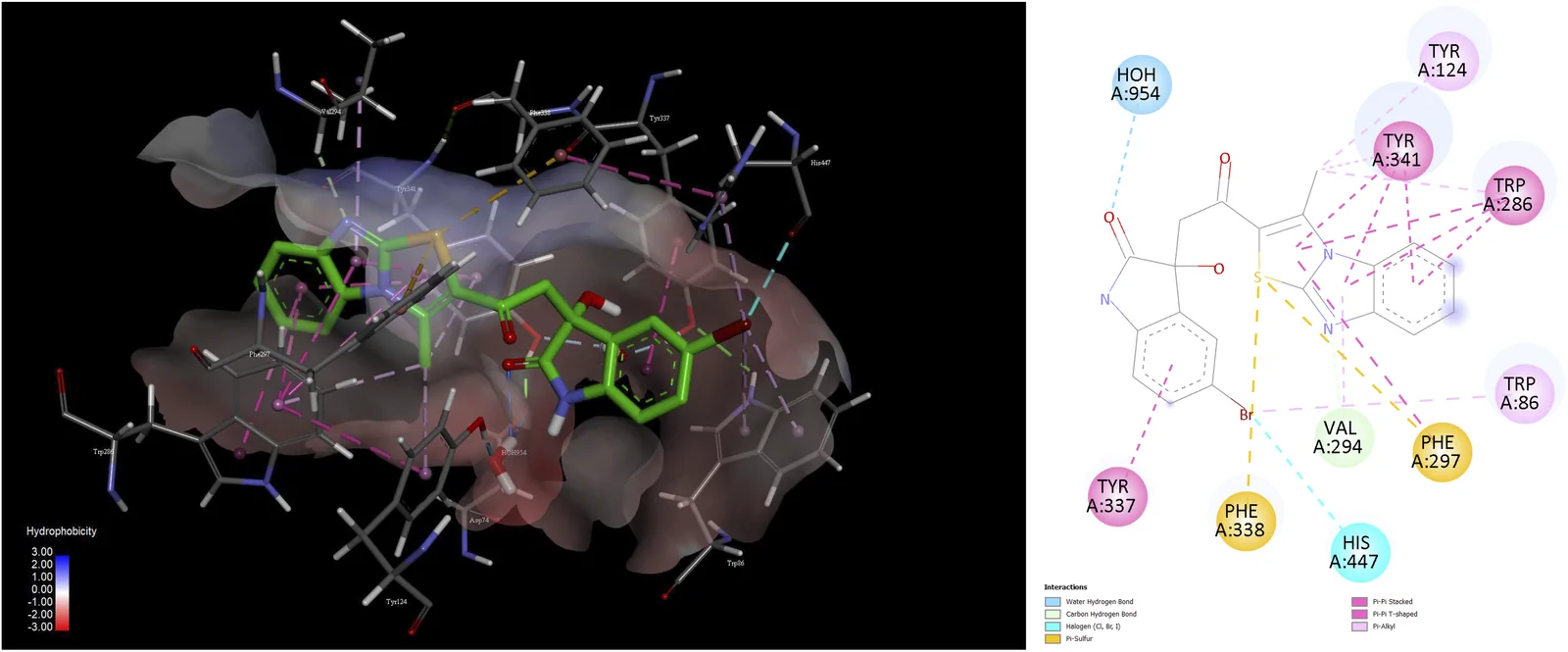
Three-dimensional (left) and two-dimensional (right) representations of the binding interactions of compound 6c within the hAChE binding pocket.

#### Molecular dynamics simulation

The docking-derived MAO-B-6c complex was subjected to a 200 ns molecular dynamics simulation to assess its stability over time, with apo MAO-B included as a reference system. Throughout the trajectory, both systems maintained consistent temperature, pressure, and potential energy profiles (Fig. 8), confirming stable simulation conditions.

**Fig. 8:**
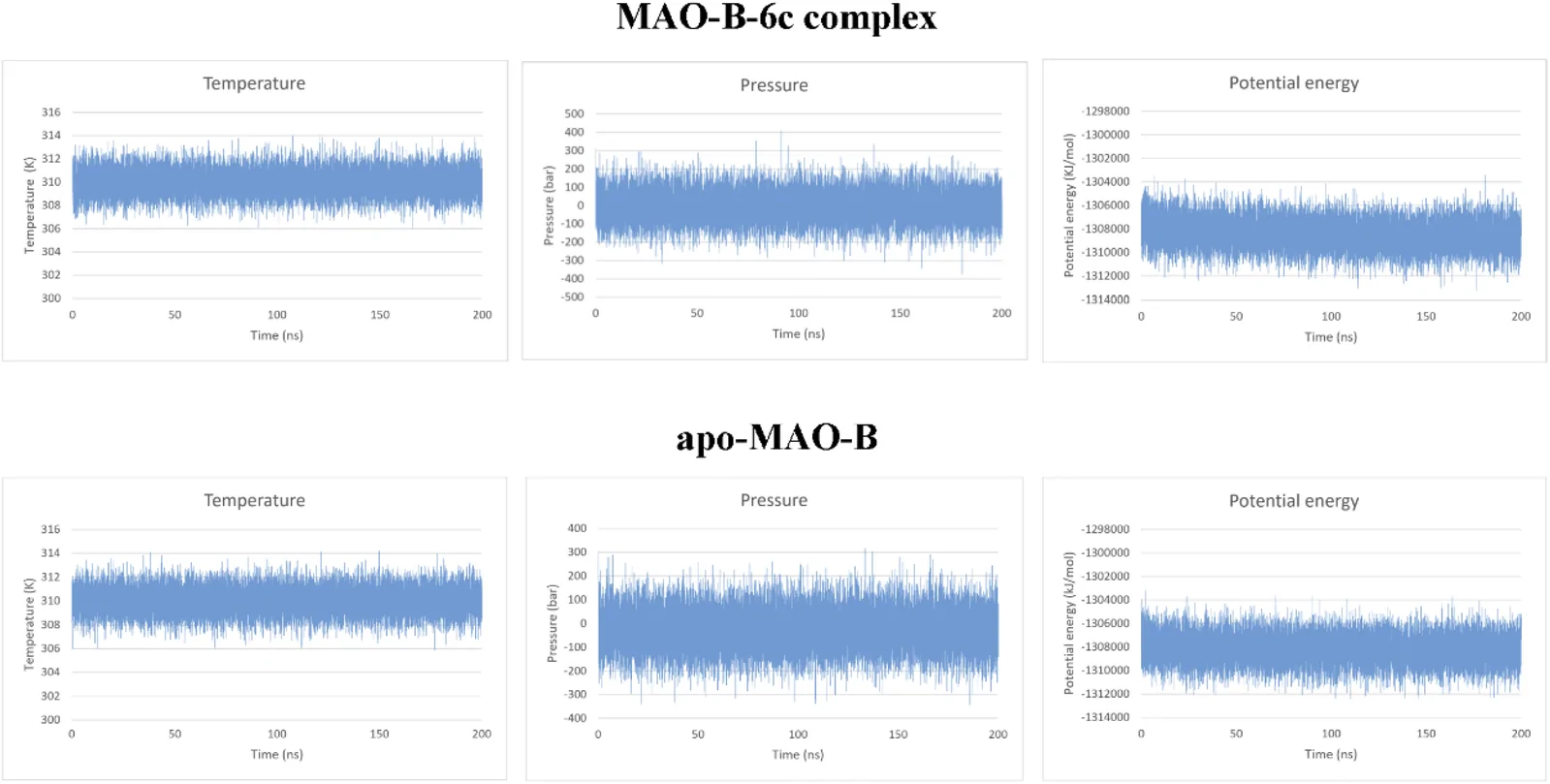
Temperature, pressure, and potential energy profiles for both MAO-B-6c complex and apo-MAO-B systems throughout the 200 ns molecular dynamics simulation.

RMSD analysis revealed an initial equilibration phase for the apo-MAO-B backbone, followed by fluctuations around 4.0–4.5 Å. By contrast, the backbone of the MAO-B-6c complex displayed lower RMSD values, remaining predominantly within 2.5–3.3 Å after equilibration. The whole-complex RMSD followed a comparable trend, with values largely ranging from 3.0 to 3.7 Å. Compound 6c itself maintained relatively low RMSD values throughout most of the trajectory, with only a modest increase toward the end of the simulation. Overall, these observations suggest that 6c retained a stable binding orientation within the MAO-B pocket during the 200 ns simulation (Fig. 9a).

**Fig. 9:**
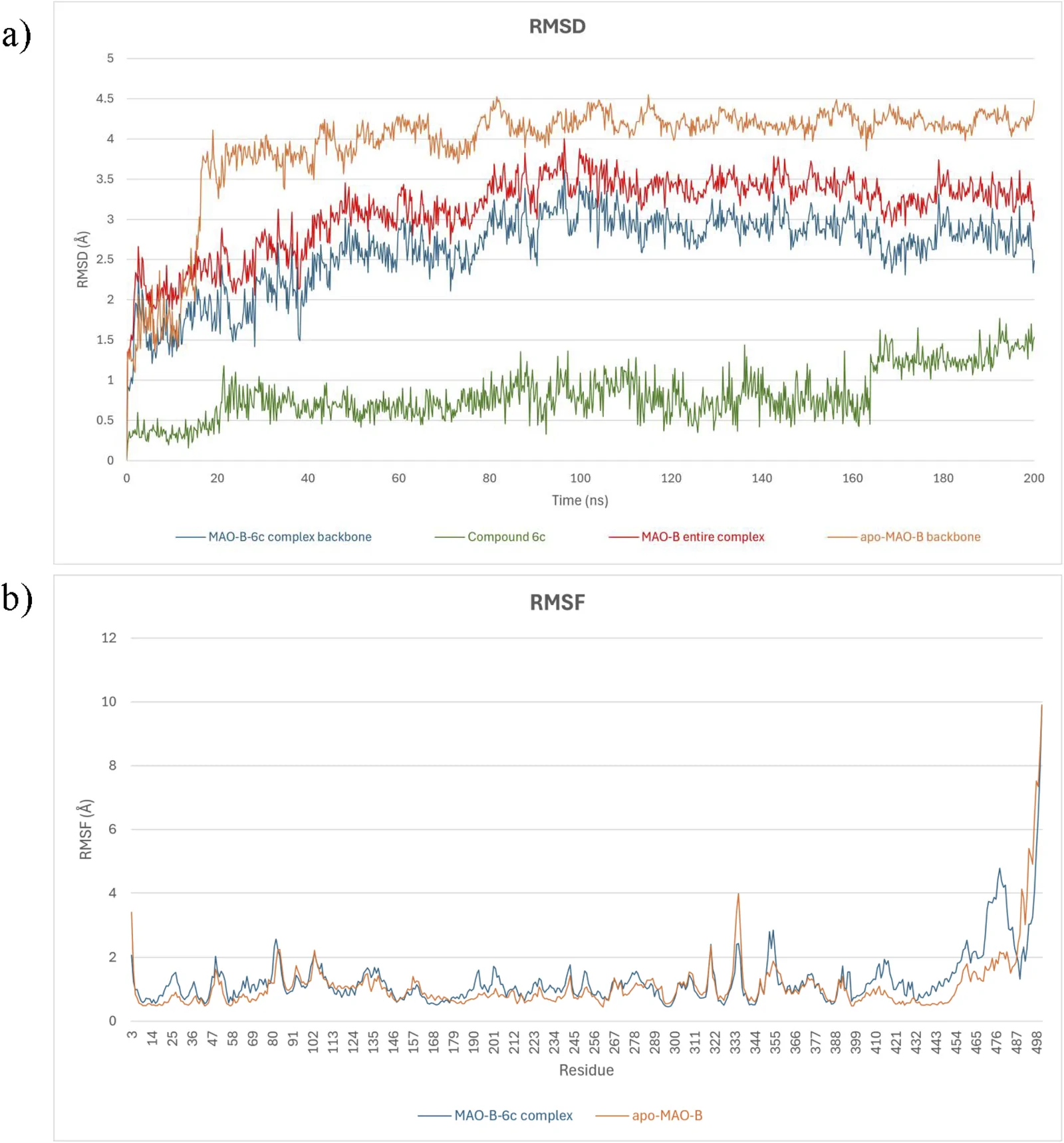
Temporal profiles of root-mean-square deviation (RMSD) (a) and root-mean-square fluctuation (RMSF) (b) for the MAO-B-6c complex and apo MAO-B during the 200 ns MD simulation.

RMSF analysis revealed broadly similar residue-fluctuation patterns for the MAO-B-6c complex and apo MAO-B. Most residues exhibited limited fluctuations, generally below 2 Å, except for the flexible terminal regions. Remarkably, the active site residues, including the gating residue Ile199, maintained low fluctuations, indicating that the binding of compound 6c did not destabilize the MAO-B active site (Fig. 9b).

To evaluate MAO-B's overall compactness and solvent exposure, the radius of gyration (*R*_g_) and solvent-accessible surface area (SASA) profiles were examined. Compound 6c caused a conformational change in MAO-B, as evidenced by slightly increased *R*_g_ and SASA values when compared to apo-MAO-B. However, the absence of continuous drift in both *R*_g_ and SASA profiles suggests that compound 6c did not destabilize the protein's overall structure (Fig. 10a and b).

**Fig. 10:**
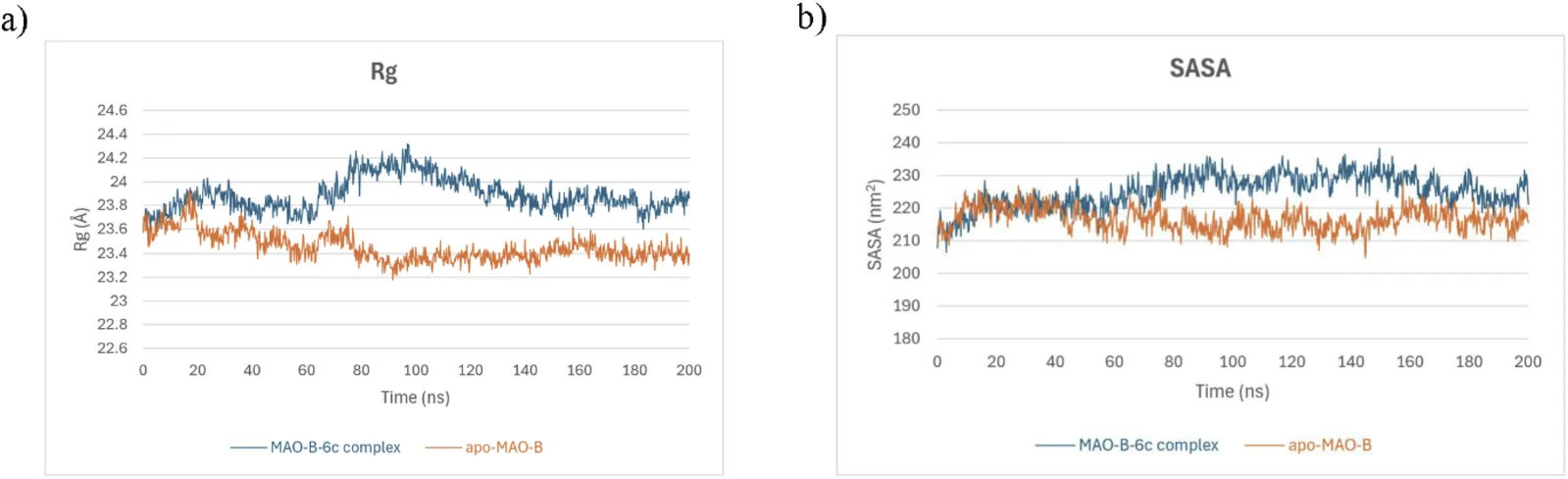
Time-dependent changes in the radius of gyration (*R*_g_) (a) and solvent-accessible surface area (SASA) (b) of the MAO-B-6c complex and apo MAO-B over the 200 ns MD trajectory.

Throughout the 200 ns simulation, the distance between the center-of-mass of compound 6c and the gating residue Ile199 usually stayed between 6.5 and 8 Å, with occasional variations. This result shows that compound 6c stayed stable in the MAO-B binding site (Fig. 11).

**Fig. 11:**
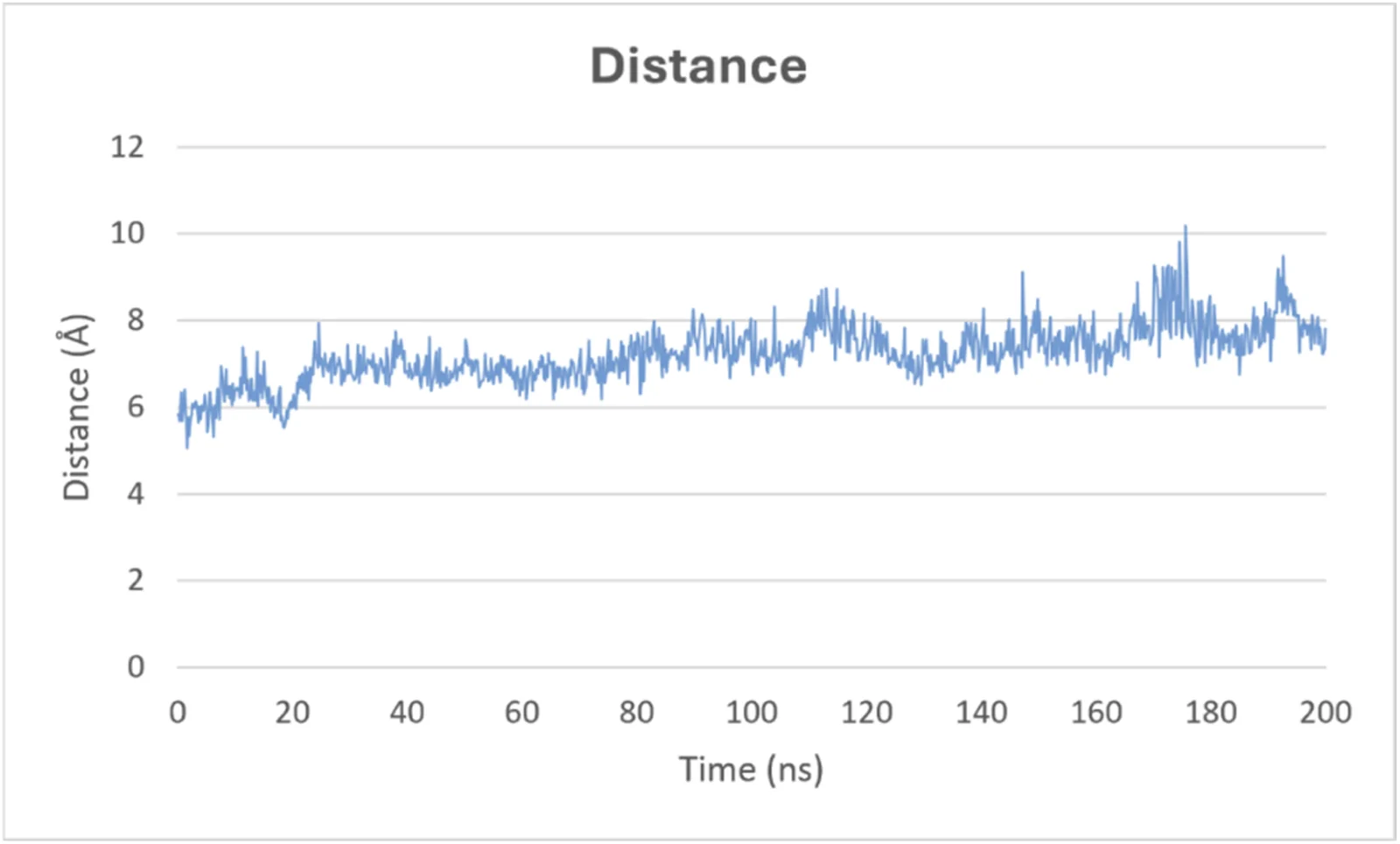
The distance between the compound 6c center-of-mass and Ile199 gating residue throughout the 200 ns molecular dynamics simulation.

Finally, the MM/PBSA analysis indicated an energetically favorable MAO-B-6c complex, with van der Waals contacts providing the dominant contribution. This is consistent with the largely hydrophobic character of the MAO-B bipartite cavity and compound 6c, which extends across both the entrance and the substrate cavities. Electrostatic interactions contributed to a smaller extent and were largely offset by the unfavorable polar solvation term. Also, the standard deviation across the analyzed frames indicates the expected level of fluctuation. It should be noted that the applied protocol does not include a configurational entropy term and relies on an implicit solvent description (Table 4).

**Table 4:** MM/PBSA-derived binding energetics for the MAO-B-6c complex (kcal mol^−1^)

Energy component (kcal mol^−1^)	Δ*G* (MM/PBSA) ± SD^a^
Electrostatic energy	−12.37 ± 3.85
van der Waals energy	−53.60 ± 3.01
Polar solvation energy	37.78 ± 4.95
Nonpolar solvation energy	−4.49 ± 0.15
Solvation free energy	33.30 ± 4.93
Gas-phase interaction energy	−65.97 ± 4.78
Overall MM/PBSA binding free energy	−32.67 ± 6.54

a Values represent the average over 1001 frames extracted from the 200 ns trajectory, reported as the mean plus or minus the sample standard deviation. Calculations were performed with gmx_MMPBSA v1.6.4 using the internal PBSA solver. Entropic contributions were not included, so the reported values are relative binding energies and not absolute binding free energies.

## Conclusion

In the present study, a series of 13 novel 3-hydroxyindolin-2-one-benzo[4,5]imidazo[2,1-*b*]thiazole hybrids was successfully designed, synthesized, and structurally characterized using ^1^H and ^13^C NMR and elemental analysis. The synthesized compounds were evaluated for their inhibitory activities against MAO-A and MAO-B enzymes. Most derivatives exhibited weak MAO-A inhibition, with preferential MAO-B inhibition, indicating a favorable selectivity profile. Among the investigated compounds, the 5-bromo (6c) and 5-chloro (6b) derivatives emerged as the most potent MAO-B inhibitors, with IC_50_ values of 0.90 ± 0.005 and 0.97 ± 0.010 µM, respectively. Structure–activity relationship analysis revealed that substitution at the C-5 position of the isatin ring, particularly with halogen atoms, significantly enhanced MAO-B inhibitory activity, whereas *N*-alkylation generally reduced both potency and selectivity. Based on their superior MAO-B profiles, compounds 6b and 6c were further evaluated for their AChE and BuChE inhibitory activities. Notably, compound 6c exhibited potent dual cholinesterase inhibition, with AChE inhibitory activity comparable to that of donepezil and superior BuChE inhibition. In addition, SwissADME analysis suggested favorable drug-likeness for the most active compounds, while molecular docking and 200-ns molecular dynamics simulations supported stable binding of the lead compound within the MAO-B active site and provided a plausible explanation for its observed biological activity. Collectively, these findings identify compound 6c as a promising lead molecule that combines selective MAO-B inhibition with potent cholinesterase inhibitory activity, warranting further investigation as a multitarget-directed candidate for the management of Alzheimer's disease.

## Experimental

### Chemistry

#### General

The National Research Center (NRC), Thermo Fisher, SRL, Sigma-Aldrich, Alfa Aesar, or Merck provided the materials, which were utilized exactly as supplied. Every synthetic procedure was carried out at predetermined temperatures and in environmental circumstances. Melting points were determined using the one-open-at-one-end capillary method with the Stuart (Cole-Parmer) MP-400D (SMP30), and the results were shown without correction. Using *n*-hexane : ethyl acetate (in different ratios) as the developing solvent at 254 nm, thin-layer chromatography (TLC) was used to track the reactions. A PerkinElmer 2400 CHNS analyzer was used to do elemental analysis (% C, H, N, and S). DMSO-*d*_6_ was used as the solvent when recording the ^1^H NMR (400 MHz) and ^13^C NMR-DEPTQ (101 MHz) spectra on a Bruker Avance III 600 spectrometer. When necessary, modest volumes of deuterated ethanol were added to improve chemical solubility. Chemical shifts (*δ*) are expressed in parts per million (ppm), while coupling constants (*J*) are expressed in hertz (Hz). Some compounds had comparatively weak ^13^C NMR signals due to their restricted solubility in the NMR solvent. To increase sample solubility and spectral quality, tiny aliquots of appropriate deuterated co-solvents were added as needed. This process allowed for increased signal strength and made it easier to identify the carbon resonances.

##### General steps for the preparation of target compounds (6a–e and 8a–h)

A mixture of the appropriate isatin derivative (5a–e or 7a–h, 1 mmol) and ketone 4 (1 mmol) in absolute ethanol (20 mL) containing diethylamine (2 drops) was stirred at room temperature overnight. The resulting yellow precipitate of the corresponding 3-hydroxy derivatives was collected by filtration and recrystallized from ethanol. The obtained solid was washed successively with distilled water, absolute ethanol, and diethyl ether, then dried to afford the target compounds 6a–e and 8a–h.

###### 3-Hydroxy-3-(2-(3-methylbenzo[4,5]imidazo[2,1-*b*]thiazol-2-yl)-2-oxoethyl)indolin-2-one (6a)

White powder (90%), with M.P. 255 °C. ^1^H NMR (400 MHz, DMSO-*d*_6_) *δ* (ppm): 10.33 (s, 1H, CONH), 8.04 (d, *J* = 7.9 Hz, 1H, proton of the aromatic ring), 7.70 (d, *J* = 8.1 Hz, 1H, proton of the aromatic ring), 7.41 (t, *J* = 7.7 Hz, 1H, proton of the aromatic ring), 7.36 (d, *J* = 7.4 Hz, 1H, proton of the aromatic ring), 7.30 (t, *J* = 7.8 Hz, 1H, proton of the aromatic ring), 7.20 (t, *J* = 7.7 Hz, 1H, proton of the aromatic ring), 6.91 (t, *J* = 7.5 Hz, 1H, proton of the aromatic ring), 6.84 (d, *J* = 7.8 Hz, 1H, proton of the aromatic ring), 6.19 (s, 1H, OH), 3.92 (d, *J* = 17.2 Hz, 1H, COCH_2_), 3.58 (d, *J* = 17.2 Hz, 1H, COCH_2_), 3.07 (s, 3H, CH_3_). ^13^C NMR (101 MHz, DMSO-*d*_6_) *δ* (ppm): 190.17 (**C**OCH_2_), 178.45 (CONH), 154.15 (**C**_3_-CH_3_), 148.78, 143.35, 139.34, 131.88, 130.75, 129.57, 124.98, 124.27, 122.30, 121.94, 121.70, 119.19, 113.30, 109.99, 73.33 (**C**_3_-OH), 48.90 (CO**C**H_2_), 14.83 (CH_3_). Elemental analysis of compound C_20_H_15_N_3_O_3_S demonstrated close agreement between the calculated values [C, 63.65%; H, 4.01%; N, 11.13%; S, 8.49%] and the experimentally determined values [C, 63.80%; H, 4.00%; N, 11.15%; S, 8.54%].

###### 5-Chloro-3-hydroxy-3-(2-(3-methylbenzo[4,5]imidazo[2,1-*b*]thiazol-2-yl)-2-oxoethyl)indolin-2-one (6b)

White powder (85%), with M.P. 248 °C. ^1^H NMR (400 MHz, DMSO-*d*_6_) *δ* (ppm): 10.47 (s, 1H, CONH), 8.06 (d, *J* = 7.9 Hz, 1H, proton of the aromatic ring), 7.70 (d, *J* = 8.2 Hz, 1H, proton of the aromatic ring), 7.59 (s, 1H, proton of the aromatic ring), 7.44–7.27 (m, 3H, protons of the aromatic ring), 6.81 (d, *J* = 8.3 Hz, 1H, proton of the aromatic ring), 6.33 (s, 1H, OH), 4.06 (d, *J* = 17.4 Hz, 1H, 1H, COCH_2_), 3.62 (d, *J* = 17.4 Hz, 1H, 1H, COCH_2_), 3.08 (s, 3H, CH_3_). ^13^C NMR (101 MHz, DMSO-*d*_6_) *δ* (ppm): 190.27 (**C**OCH_2_), 178.07 (CONH), 154.21 (**C**_3_-CH_3_), 148.81, 142.74, 139.55, 134.48, 132.14, 130.79, 127.33, 125.00, 121.99, 119.21, 113.36, 111.94, 73.29 (**C**_3_-OH), 48.73 (CO**C**H_2_), 14.91 (CH_3_). Elemental analysis of compound C_20_H_14_ClN_3_O_3_S demonstrated close agreement between the calculated values [C, 58.33%; H, 3.43%; N, 10.20%; S, 7.78%] and the experimentally determined values [C, 58.14%; H, 3.42%; N, 10.17%; S, 7.77%].

###### 5-Bromo-3-hydroxy-3-(2-(3-methylbenzo[4,5]imidazo[2,1-*b*]thiazol-2-yl)-2-oxoethyl)indolin-2-one (6c)

White powder (86%), with M.P. 240 °C. ^1^H NMR (400 MHz, DMSO-*d*_6_) *δ* (ppm): 10.47 (s, 1H, CONH), 8.06 (d, *J* = 8.2 Hz, 1H, proton of the aromatic ring), 7.70 (d, *J* = 8.1 Hz, 1H, proton of the aromatic ring), 7.48 (d, *J* = 2.3 Hz, 1H, proton of the aromatic ring), 7.41 (t, *J* = 7.7 Hz, 1H, proton of the aromatic ring), 7.31 (t, *J* = 7.5 Hz, 1H, proton of the aromatic ring), 7.26 (dd, *J* = 8.3, 2.3 Hz, 1H, proton of the aromatic ring), 6.86 (d, *J* = 8.3 Hz, 1H, proton of the aromatic ring), 6.34 (s, 1H, OH), 4.06 (d, *J* = 17.5 Hz, 1H, COCH_2_), 3.63 (d, *J* = 17.5 Hz, 1H, COCH_2_), 3.08 (s, 3H, CH_3_). ^13^C NMR (101 MHz, DMSO-*d*_6_) *δ* (ppm): 190.28 (**C**OCH_2_), 178.20 (CONH), 154.20 (**C**_3_-CH_3_), 148.80, 142.33, 139.58, 134.08, 130.77, 129.28, 125.73, 125.02, 124.62, 122.30, 121.97, 119.22, 113.31, 111.40, 73.33 (**C**_3_-OH), 48.72 (CO**C**H_2_), 14.90 (CH_3_). Elemental analysis of compound C_20_H_14_BrN_3_O_3_S demonstrated close agreement between the calculated values [C, 52.64%; H, 3.09%; N, 9.21%; S, 7.03%] and the experimentally determined values [C, 52.85%; H, 3.10%; N, 9.24%; S, 7.00%].

###### 3-Hydroxy-5-methyl-3-(2-(3-methylbenzo[4,5]imidazo[2,1-*b*]thiazol-2-yl)-2-oxoethyl)indolin-2-one (6d)

Buff powder (79%), with M.P. 238 °C. ^1^H NMR (400 MHz, DMSO-*d*_6_) *δ* (ppm): 10.21 (s, 1H, CONH), 8.05 (d, *J* = 8.1 Hz, 1H, proton of the aromatic ring), 7.70 (d, *J* = 8.1 Hz, 1H, proton of the aromatic ring), 7.41 (t, *J* = 8.1 Hz, 1H, proton of the aromatic ring), 7.30 (t, *J* = 8.0 Hz, 1H, proton of the aromatic ring), 7.18 (s, 1H, proton of the aromatic ring), 7.00 (d, *J* = 7.9 Hz, 1H, proton of the aromatic ring), 6.72 (d, *J* = 7.9 Hz, 1H, proton of the aromatic ring), 6.13 (s, 1H, OH), 3.89 (d, *J* = 17.5 Hz, 1H, COCH_2_), 3.57 (d, *J* = 17.5 Hz, 1H, COCH_2_), 3.07 (s, 3H, CH_3_), 2.20 (s, 3H, CH_3_). ^13^C NMR (101 MHz, DMSO-*d*_6_) *δ* (ppm): 190.16 (**C**OCH_2_), 178.45 (CONH), 154.17 (**C**_3_-CH_3_), 148.78, 140.86, 139.32, 131.94, 130.77, 130.42, 129.68, 124.98, 124.92, 122.35, 121.94, 119.20, 113.31, 109.72, 73.41 (**C**_3_-OH), 48.94 (CO**C**H_2_), 21.14 (CH_3_), 14.84 (CH_3_). Elemental analysis of compound C_21_H_17_N_3_O_3_S demonstrated close agreement between the calculated values [C, 64.44%; H, 4.38%; N, 10.73%; S, 8.19%] and the experimentally determined values [C, 64.62%; H, 4.40%; N, 10.76%; S, 8.23%].

###### 3-Hydroxy-7-methyl-3-(2-(3-methylbenzo[4,5]imidazo[2,1-*b*]thiazol-2-yl)-2-oxoethyl)indolin-2-one (6e)

Brown powder (75%), with M.P. 232 °C. ^1^H NMR (400 MHz, DMSO-*d*_6_) *δ* (ppm): 10.36 (s, 1H, CONH), 8.05 (d, *J* = 8.0 Hz, 1H, proton of the aromatic ring), 7.70 (d, *J* = 8.1 Hz, 1H, proton of the aromatic ring), 7.41 (s, 1H, proton of the aromatic ring), 7.33–7.30 (m, 1H, proton of the aromatic ring), 7.18 (d, *J* = 7.2 Hz, 1H, proton of the aromatic ring), 7.02 (d, *J* = 7.6 Hz, 1H, proton of the aromatic ring), 6.82 (t, *J* = 7.5 Hz, 1H, proton of the aromatic ring), 6.14 (s, 1H, OH), 3.90 (d, *J* = 17.1 Hz, 1H, COCH_2_), 3.58 (d, *J* = 17.1 Hz, 1H, COCH_2_), 3.08 (s, 3H, CH_3_), 2.23 (s, 3H, CH_3_). ^13^C NMR (101 MHz, DMSO-*d*_6_) *δ* (ppm): 190.19 (**C**OCH_2_), 178.90 (CONH), 154.17 (**C**_3_-CH_3_), 148.78, 141.81, 139.91, 139.32, 138.90, 131.54, 130.88, 130.83, 130.77, 124.98, 124.91, 123.56, 123.06, 122.49, 122.40, 122.02, 121.94, 121.89, 121.69, 121.57, 119.20, 119.11, 113.32, 113.24, 73.52 (**C**_3_-OH), 48.96 (CO**C**H_2_), 16.84 (CH_3_), 14.86 (CH_3_). Elemental analysis of compound C_21_H_17_N_3_O_3_S demonstrated close agreement between the calculated values [C, 64.44%; H, 4.38%; N, 10.73%; S, 8.19%] and the experimentally determined values [C, 64.65%; H, 4.35%; N, 10.80%; S, 8.15%].

###### 3-Hydroxy-1-methyl-3-(2-(3-methylbenzo[4,5]imidazo[2,1-*b*]thiazol-2-yl)-2-oxoethyl)indolin-2-one (8a)

Orange powder (79%), with M.P. 220 °C. ^1^H NMR (400 MHz, DMSO-*d*_6_) *δ* (ppm): 8.05 (d, *J* = 7.9 Hz, 1H, proton of the aromatic ring), 7.70 (d, *J* = 8.0 Hz, 1H, proton of the aromatic ring), 7.43–7.39 (m, 2H, protons of the aromatic ring), 7.30 (ddd, *J* = 7.4, 6.5, 1.1 Hz, 2H, protons of the aromatic ring), 7.03–6.97 (m, 2H, protons of the aromatic ring), 6.27 (s, 1H, OH), 4.00 (d, *J* = 17.3 Hz, 1H, COCH_2_), 3.66 (d, *J* = 17.3 Hz, 1H, COCH_2_), 3.17 (s, 3H, NCH_3_), 3.07 (s, 3H, CH_3_). ^13^C NMR (101 MHz, DMSO-*d*_6_) *δ* (ppm): 190.12 (**C**OCH_2_), 176.77 (CONH), 154.17 (**C**_3_-CH_3_), 148.77, 144.80, 139.47, 131.20, 130.76, 129.74, 125.01, 123.84, 122.43, 121.97, 119.21, 119.19, 113.32, 108.82, 72.99 (**C**_3_-OH), 49.10 (CO**C**H_2_), 26.48 (NCH_3_), 14.90 (CH_3_). Elemental analysis of compound C_21_H_17_N_3_O_3_S demonstrated close agreement between the calculated values [C, 64.44%; H, 4.38%; N, 10.73%; S, 8.19%] and the experimentally determined values [C, 64.72%; H, 4.37%; N, 10.75%; S, 8.17%].

###### 5-Chloro-3-hydroxy-1-methyl-3-(2-(3-methylbenzo[4,5]imidazo[2,1-*b*]thiazol-2-yl)-2-oxoethyl)indolin-2-one (8b)

Purple powder (68%), with M.P. 223 °C. ^1^H NMR (400 MHz, DMSO-*d*_6_) *δ* (ppm): 8.07 (d, *J* = 8.2 Hz, 1H, proton of the aromatic ring), 7.71 (d, *J* = 8.2 Hz, 1H, proton of the aromatic ring), 7.55 (d, *J* = 2.2 Hz, 1H, proton of the aromatic ring), 7.44–7.34 (m, 3H, protons of the aromatic ring), 7.05 (d, *J* = 8.3 Hz, 1H, proton of the aromatic ring), 6.41 (s, 1H, OH), 4.13 (d, *J* = 17.6 Hz, 1H, COCH_2_), 3.70 (d, *J* = 17.6 Hz, 1H, COCH_2_), 3.17 (s, 3H, NCH_3_), 3.09 (s, 3H, CH_3_). ^13^C-NMR (DEPTQ ↑/↓) (*δ*): 190.31 (↓), 176.56 (↓), 154.28 (↓), 148.89 (↓), 143.84 (↓), 139.80 (↓), 133.43 (↓), 130.88 (↓), 129.44 (↑), 126.58 (↑), 125.11 (↑), 124.35 (↑), 122.44 (↑), 122.06 (↑), 119.32 (↑), 113.43 (↑), 110.38 (↑), 73.00 (↓), 49.00 (↓), 26.70 (↑), 15.07 (↑). Elemental analysis of compound C_21_H_16_ClN_3_O_3_S demonstrated close agreement between the calculated values [C, 59.22%; H, 3.79%; N, 9.87%; S, 7.53%] and the experimentally determined values [C, 58.99%; H, 3.80%; N, 9.89%; S, 7.56%].

###### 5-Bromo-3-hydroxy-1-methyl-3-(2-(3-methylbenzo[4,5]imidazo[2,1-*b*]thiazol-2-yl)-2-oxoethyl)indolin-2-one (8c)

Beige powder (69%), with M.P. 226 °C. ^1^H NMR (400 MHz, DMSO-*d*_6_) *δ* (ppm): 8.05 (d, *J* = 8.4 Hz, 1H, proton of the aromatic ring), 7.77–7.59 (m, 2H, protons of the aromatic ring), 7.52–7.27 (m, 3H, protons of the aromatic ring), 7.00 (d, *J* = 8.5 Hz, 1H, proton of the aromatic ring), 6.41 (s, 1H, OH), 4.13 (d, *J* = 17.7 Hz, 1H, COCH_2_), 3.69 (d, *J* = 17.7 Hz, 1H, COCH_2_), 3.17 (s, 3H, NCH_3_), 3.07 (s, 3H, CH_3_). ^13^C NMR (101 MHz, DMSO-*d*_6_) *δ* (ppm): 190.23 (**C**OCH_2_), 176.38 (CONH), 154.21 (**C**_3_-CH_3_), 148.79, 144.19, 139.67, 133.71, 132.25, 130.76, 126.95, 125.03, 122.38, 121.99, 119.22, 114.29, 113.29, 110.87, 72.90 (**C**_3_-OH), 48.92 (CO**C**H_2_), 26.60 (NCH_3_), 14.96 (CH_3_). Elemental analysis of compound C_21_H_16_BrN_3_O_3_S demonstrated close agreement between the calculated values [C, 53.63%; H, 3.43%; N, 8.93%; S, 6.82%] and the experimentally determined values [C, 53.49%; H, 3.44%; N, 8.96%; S, 6.79%].

###### 1-Ethyl-3-hydroxy-3-(2-(3-methylbenzo[4,5]imidazo[2,1-*b*]thiazol-2-yl)-2-oxoethyl)indolin-2-one (8d)

White powder (74%), with M.P. 224 °C. ^1^H NMR (400 MHz, DMSO-*d*_6_) *δ* (ppm): 8.03 (d, *J* = 8.1 Hz, 1H, proton of the aromatic ring), 7.69 (d, *J* = 8.1 Hz, 1H, proton of the aromatic ring), 7.45–7.37 (m, 2H, protons of the aromatic ring), 7.33–7.26 (m, 2H, protons of the aromatic ring), 7.04 (d, *J* = 7.8 Hz, 1H, proton of the aromatic ring), 6.98 (t, *J* = 7.5 Hz, 1H, proton of the aromatic ring), 6.26 (s, 1H, OH), 3.98 (d, *J* = 17.2 Hz, 1H, COCH_2_), 3.74 (q, *J* = 7.1 Hz, 2H, N**CH**_**2**_CH_3_), 3.65 (d, *J* = 17.2 Hz, 1H, COCH_2_), 3.05 (s, 3H, CH_3_), 1.22 (t, *J* = 7.1 Hz, 3H, NCH_2_**CH**_**3**_). ^13^C NMR (101 MHz, DMSO-*d*_6_) *δ* (ppm): 190.02, 176.34, 154.15, 148.76, 143.81, 139.35, 131.36, 130.73, 129.73, 124.99, 124.05, 122.31, 122.21, 121.95, 119.19, 113.28, 108.90, 72.99 (**C**_3_-OH), 49.17 (CO**C**H), 34.41 (N**CH**_**2**_CH_3_), 14.85 (NCH_2_**CH**_**3**_), 12.61 (CH_3_). Elemental analysis of compound C_22_H_19_N_3_O_3_S demonstrated close agreement between the calculated values [C, 65.17%; H, 4.72%; N, 10.36%; S, 7.91%] and the experimentally determined values [C, 64.96%; H, 4.69%; N, 10.30%; S, 7.94%].

###### 3-Hydroxy-3-(2-(3-methylbenzo[4,5]imidazo[2,1-*b*]thiazol-2-yl)-2-oxoethyl)-1-propylindolin-2-one (8e)

White powder (87%), with M.P. 222 °C. ^1^H NMR (400 MHz, DMSO-*d*_6_) *δ* (ppm): 8.02 (d, *J* = 8.2 Hz, 1H, proton of the aromatic ring), 7.66 (d, *J* = 8.1 Hz, 1H, proton of the aromatic ring), 7.37 (dt, *J* = 7.7, 4.0 Hz, 2H, protons of the aromatic ring), 7.26 (dt, *J* = 11.9, 7.8 Hz, 2H, protons of the aromatic ring), 7.00 (d, *J* = 7.8 Hz, 1H, protons of the aromatic ring), 6.93 (t, *J* = 7.5 Hz, 1H, protons of the aromatic ring), 6.21 (s, 1H, OH), 3.94 (d, *J* = 17.2 Hz, 1H, COCH_2_), 3.65–3.56 (m, 3H, COCH_2_ and N**CH**_**2**_CH_2_CH_3_), 3.03 (s, 3H, CH_3_), 1.64 (h, *J* = 7.4 Hz, 2H, NCH_2_**CH**_**2**_CH_3_), 0.92 (t, *J* = 7.4 Hz, 3H, NCH_2_CH_2_**CH**_**3**_). ^13^C NMR (101 MHz, DMSO-*d*_6_) *δ* (ppm): 190.10, 176.81, 154.22, 148.86, 144.36, 139.45, 131.33, 130.84, 129.75, 125.05, 124.05, 122.36, 122.23, 122.01, 119.28, 113.39, 109.07, 73.01, 49.16, 41.41, 20.80, 14.94, 11.80. Elemental analysis of compound C_23_H_21_N_3_O_3_S demonstrated close agreement between the calculated values [C, 65.85%; H, 5.05%; N, 10.02%; S, 7.64%] and the experimentally determined values [C, 66.02%; H, 5.03%; N, 9.99%; S, 7.66%].

###### 3-Hydroxy-1-isobutyl-3-(2-(3-methylbenzo[4,5]imidazo[2,1-*b*]thiazol-2-yl)-2-oxoethyl)indolin-2-one (8f)

Pale brown powder (72%), with M.P. 212 °C. ^1^H NMR (400 MHz, DMSO-*d*_6_) *δ* (ppm): 8.05 (d, *J* = 8.1 Hz, 1H, proton of the aromatic ring), 7.70 (d, *J* = 8.1 Hz, 1H, proton of the aromatic ring), 7.43–7.39 (m, 2H, protons of the aromatic ring), 7.32–7.26 (m, 2H, protons of the aromatic ring), 7.04 (d, *J* = 7.8 Hz, 1H, proton of the aromatic ring), 6.97 (t, *J* = 7.5 Hz, 1H, proton of the aromatic ring), 6.27 (s, 1H, OH), 3.99 (d, *J* = 17.2 Hz, 1H, COCH_2_), 3.66 (d, *J* = 17.3 Hz, 1H, COCH_2_), 3.49 (dd, *J* = 7.3, 3.3 Hz, 2H, **CH**_**2**_CH), 3.07 (s, 3H, CH_3_), 2.11 (dt, *J* = 13.7, 6.9 Hz, 1H, **CH**(CH_3_)_2_), 0.97 (dd, *J* = 6.7, 4.8 Hz, 6H, CH(**CH**_**3**_)_2_). ^13^C NMR (101 MHz, DMSO-*d*_6_) *δ* (ppm): 190.05, 177.01, 154.15, 148.78, 144.65, 139.42, 131.19, 130.76, 129.64, 125.00, 123.92, 122.28, 122.16, 121.95, 119.20, 113.32, 109.22, 72.90 (**C**_3_-OH), 48.97 (**CH**_**2**_CH), 47.31 (CO**C**H), 27.31 (**CH**(CH_3_)_2_), 20.61 (CH(**CH**_**3**_)_2_), 20.58 (CH(**CH**_**3**_)_2_), 14.86 (CH_3_). Elemental analysis of compound C_24_H_23_N_3_O_3_S demonstrated close agreement between the calculated values [C, 66.49%; H, 5.35%; N, 9.69%; S, 7.40%] and the experimentally determined values [C, 66.32%; H, 5.33%; N, 9.70%; S, 7.43%].

###### 1-Benzyl-3-hydroxy-3-(2-(3-methylbenzo[4,5]imidazo[2,1-*b*]thiazol-2-yl)-2-oxoethyl)indolin-2-one (8g)

White powder (66%), with M.P. 232 °C. ^1^H NMR (400 MHz, DMSO-*d*_6_) *δ* (ppm): 8.07 (d, *J* = 4.6 Hz, 1H, proton of the aromatic ring), 7.71 (d, *J* = 8.1 Hz, 1H, proton of the aromatic ring), 7.59–7.56 (m, 1H, proton of the aromatic ring), 7.50–7.43 (m, 4H, protons of the aromatic ring), 7.34 (dd, *J* = 5.0, 2.6 Hz, 3H, protons of the aromatic ring), 7.20 (td, *J* = 7.7, 1.2 Hz, 1H, proton of the aromatic ring), 6.97 (d, *J* = 7.6 Hz, 1H, proton of the aromatic ring), 6.82 (d, *J* = 7.8 Hz, 1H, proton of the aromatic ring), 6.42 (s, 1H, OH), 4.92 (d, *J* = 9.5 Hz, 2H, **CH**_**2**_Ph), 4.06 (d, *J* = 17.2 Hz, 1H, COCH_2_), 3.74 (d, *J* = 17.2 Hz, 1H, COCH_2_), 3.10 (s, 3H, CH_3_). ^13^C-NMR (DEPTQ ↑/↓) (*δ*): 190.22 (↓), 177.06 (↓), 154.24 (↓), 148.89 (↓), 143.96 (↓), 139.67 (↓), 136.94 (↓), 131.35 (↓), 130.84 (↓), 129.68 (↑), 128.97 (↑), 127.91 (↑), 127.85 (↑), 127.80 (↑), 125.09 (↑), 124.12 (↑), 122.59 (↑), 122.15 (↓), 122.03 (↑), 119.30 (↑), 113.42 (↑), 109.56 (↑), 73.19 (↓), 49.10 (↓), 43.33 (↓), 14.94 (↑). Elemental analysis of compound C_27_H_21_N_3_O_3_S demonstrated close agreement between the calculated values [C, 69.36%; H, 4.53%; N, 8.99%; S, 6.86%] and the experimentally determined values [C, 69.19%; H, 4.55%; N, 9.02%; S, 6.90%].

###### 3-Hydroxy-3-(2-(3-methylbenzo[4,5]imidazo[2,1-*b*]thiazol-2-yl)-2-oxoethyl)-1-phenethylindolin-2-one (8h)

White powder (76%), with M.P. 236 °C. ^1^H NMR (400 MHz, DMSO-*d*_6_) *δ* (ppm): 8.05 (d, *J* = 8.2 Hz, 1H, proton of the aromatic ring), 7.67 (d, *J* = 8.1 Hz, 1H, proton of the aromatic ring), 7.38 (t, *J* = 6.8 Hz, 2H, proton of the aromatic ring), 7.34–7.20 (m, 7H, proton of the aromatic ring), 7.01 (d, *J* = 7.8 Hz, 1H, proton of the aromatic ring), 6.94 (t, *J* = 7.4 Hz, 1H, proton of the aromatic ring), 6.24 (s, 1H, OH), 3.94 (d, *J* = 17.2 Hz, 1H, COCH_2_), 3.89–3.78 (m, 2H, NCH_2_**CH**_**2**_Ph), 3.59 (d, *J* = 17.2 Hz, 1H, COCH_2_), 3.06 (s, 3H, CH_3_), 2.89 (h, *J* = 6.1 Hz, 2H, NCH_2_**CH**_**2**_Ph). ^13^C-NMR (DEPTQ ↑/↓) (*δ*): 190.17 (↓), 176.68 (↓), 154.24 (↓), 148.88 (↓), 143.91 (↓), 139.60 (↓), 139.17 (↓), 131.27 (↓), 130.88 (↓), 129.79 (↑), 129.40 (↑), 129.00 (↑), 126.92 (↑), 125.09 (↑), 124.18 (↑), 122.38 (↑), 122.04 (↑), 119.31 (↑), 113.46 (↑), 109.16 (↑), 73.07 (↓), 49.24 (↓), 41.42 (↓), 33.40 (↓), 14.99 (↑). Elemental analysis of compound C_28_H_23_N_3_O_3_S demonstrated close agreement between the calculated values [C, 69.84%; H, 4.81%; N, 8.73%; S, 6.66%] and the experimentally determined values [C, 70.02%; H, 4.80%; N, 8.78%; S, 6.64%].

#### MAO-A and MAO-B inhibition assay

MAO-A and MAO-B enzymes and the reference inhibitors were obtained from Sigma-Aldrich (St. Louis, MO, USA), except for pargyline, which was purchased from BioAssay Systems (Hayward, CA, USA). Enzymatic activity was determined according to previously reported procedures,^38–41^ using kynuramine (0.06 mM) for MAO-A and benzylamine (0.3 mM) for MAO-B as the respective substrates. Product formation was continuously followed for 45 min at 316 nm for MAO-A and 250 nm for MAO-B. Measurements were carried out in 200 µL reaction mixtures using 96-well UV plates (Corning Incorporated, Kennebunk, ME, USA) and a Varioskan LUX microplate reader (Thermo Fisher Scientific, Waltham, MA, USA).

#### Cholinesterase inhibitory activity

The inhibitory effects of the synthesized compounds on acetylcholinesterase (AChE) and butyrylcholinesterase (BuChE) were determined using commercial assay kits: the AChE Inhibitor Screening Kit (Abcam, Cambridge, UK; Cat. No. ab283363) and the BuChE Activity Assay Kit (Sigma-Aldrich, St. Louis, MO, USA; Cat. No. MAK551). Donepezil served as the reference inhibitor in both assays. Stock solutions of the test compounds and donepezil were prepared in dimethyl sulfoxide (DMSO) and subsequently diluted to final concentrations of 10, 3, 1, 0.3, 0.1, 0.03, 0.01, and 0.003 µM. Enzyme inhibition was assessed by incubating AChE or BuChE with the respective compounds at different concentrations following the assay conditions recommended by the manufacturers. Enzymatic activity was quantified spectrophotometrically by recording absorbance at 414 nm using a Multiskan® EX ELISA microplate reader (Thermo Fisher Scientific, USA). All measurements were performed in triplicate, and the data are reported as mean ± SD. IC_50_ values were derived from eight-point concentration–response curves using GraphPad Prism 8.0 (GraphPad Software Inc., San Diego, CA, USA).

#### Molecular docking

The crystal structures of human MAO-B complexed with safinamide (PDB ID: 2V5Z) and human acetylcholinesterase (hAChE) bound to donepezil (PDB ID: 4EY7) were retrieved from the RCSB Protein Data Bank.^36,37^ The structure of compound 6c was generated using MarvinSketch 22.9.^42^ Protein and ligand preparation was carried out through the UCSF Chimera interface, followed by molecular docking using AutoDock Vina.^43–45^ For MAO-B, the docking search space was centered at *x* = 53.08, *y* = 155.83, and *z* = 27.01, with dimensions of 20 × 20 × 20 points along the *x*-, *y*-, and *z*-axes, respectively. For hAChE, the corresponding grid was centered at *x* = −12.32, *y* = −44.72, and *z* = 28.17 and defined by dimensions of 19.30 × 14.40 × 14.54 points along the three Cartesian axes. The resulting ligand–protein interactions were visualized in both two and three dimensions using Discovery Studio Visualizer.^46^

#### Molecular dynamics simulation protocol

The GROMACS 2026 software program was used to conduct the molecular dynamics simulation in a cubic box encircling the TIP3P water model, Na^+^, and Cl^−^ ions.^47^ Compound 6c was parametrized using the CHARMM General Force Field (CGenFF) forcefield, while MAO-B was parametrized using the CHARMM 36 forcefield.^48,49^ The energy minimization was performed using the steepest descent algorithm for 50 000 steps. The molecular dynamics simulation for 200 ns, with a 2-fs integration step, was run after equilibration using 125 ps NVT and 500 ps NPT. The pressure and temperature were set to 1 bar and 310 K, respectively, using the stochastic cell-rescaling (c-rescale) barostat and velocity-rescale (v-rescale) thermostat for coupling. The LINCS algorithm was used to restrict the hydrogen atom. The neighbor list was generated *via* the Verlet cutoff method. The particle-mesh Ewald (PME) method was used for long-range electrostatic interactions. The cutoff distance for van der Waals and coulombic interactions was set to 12 Å. Frames were saved every 200 ps throughout the 200 ns simulation. The trajectories were analyzed using the GROMACS tools.^50^ Binding free energies were estimated using the MM/PBSA method as implemented in gmx_MMPBSA v1.6.4, applying the internal PBSA solver. A total of 1001 frames extracted from the 200 ns production trajectory were analyzed, in which the complex, the receptor and the ligand are taken from the same simulation. Entropic contributions were not computed. The results are therefore used for the relative comparison of energy components and not as predictions of absolute binding affinity.^51^

## Author contributions

Conceptualization: W. M. E., H. K., and H. O. T.; supervision: W. M. E.; data curation: M. M. H., Z. M. E., and G. K.; funding acquisition: W. M. E. T. A.-W., and H. K.; methodology: A. K. E.-D., J. L., A. A. E.-H., M. R. E., H. K., and H. O. T.; visualization: A. K. E.-D., J. L., G. K., M. R. E., and H. O. T.; validation: Z. M. E., M. M. H., A. A. E.-H., and M.-J. P.; writing – original draft: W. M. E., T. A.-W., and H. O. T.; writing – review and editing: W. M. E., Z. M. E., A. A. E.-H., M.-J. P., H. K., and H. O. T. Finally, all authors approved the current submitted version of the manuscript.

## Conflicts of interest

There are no conflicts to declare.

## Supplementary Material

RA-016-D6RA05787J-s001

## Data Availability

All data generated or analyzed during this study are included in this manuscript and its supplementary information (SI). Supplementary information is available. See DOI: https://doi.org/10.1039/d6ra05787j.
