# Supplementary material for: Discovery of tricyclic benzo[4,5]imidazo[2,1-b]thiazole-3-hydroxyindolin-2-one hybrids as selective MAO-B and cholinesterase inhibitors
Source: RSC Adv. 2026 Sep 24;16(45):57349–66. doi: 10.1039/d6ra05787j (PMC13611827; doi:10.1039/d6ra05787j)
Supplement: Supplementary file 1 [file RA-016-D6RA05787J-s001.pdf]

Supplementary Information (SI) for RSC Advances.
This journal is © The Royal Society of Chemistry

Supporting Information

Discovery of Tricyclic Benzo[4,5]imidazo[2,1-*b*]thiazole-3-Hydroxyindolin-2-one Hybrids as Selective MAO-B and Cholinesterase Inhibitors

Wagdy M. Eldehna [1,*,#], Ashraf K. El-Damasy [2,*,#], Jaeyeop Lim [3,#], Mariam M. Hewala [4], Zainab M. Elsayed [4], Tarfah Al-Warhi [5], Anwar A. El-Hamaky [6], Man-Jeong Paik [3], Gyochang Keum [7], Mohamed R. Elnagar [8,9], Hoon Kim [3,*], Haytham O. Tawfik [6,10]

[1] *Department of Pharmaceutical Chemistry, Faculty of Pharmacy, Kafrelsheikh University, Kafrelsheikh, P.O. Box 33516, Egypt*

[2] *Department of Medicinal Chemistry, Faculty of Pharmacy, Mansoura University, Mansoura 35516, Egypt*

[3] *College of Pharmacy, Sunchon National University, Suncheon 57922, Republic of Korea*

[4] *Scientific Research and Innovation Support Unit, Faculty of Pharmacy, Kafrelsheikh University, Kafr El-Shaikh 33516, Egypt*

[5] *Department of Chemistry, College of Science, Princess Nourah Bint Abdulrahman University, P.O. Box 84428, Riyadh 11671, Saudi Arabia*

[6] *Department of Pharmaceutical Chemistry, Faculty of Pharmacy, Tanta University, Tanta 31527, Egypt*

[7] *Medicinal Materials Research Center, Biomedical Research Division, Korea Institute of Science and Technology (KIST), Hwarang-ro14gil 5, Seongbuk-gu, Seoul 02792, Republic of Korea*

[8] *Department of Pharmacology and Toxicology, Faculty of Pharmacy, Al-Azhar University, Cairo 11823, Egypt.*

[9] *Department of Pharmacology, College of Pharmacy, The Islamic University, Najaf 54001, Iraq.*

[10] *Department of Pharmaceutical Chemistry, Faculty of Pharmacy, Alsalam University, 31511, Tanta, Egypt*

[*] Correspondence: W.M.E.; wagdy2000@gmail.com, A. K. El-D.; ph_karem2000@man.edu.eg, and H. K.; hoon@sunchon.ac.kr.

[#] These authors contributed equally.

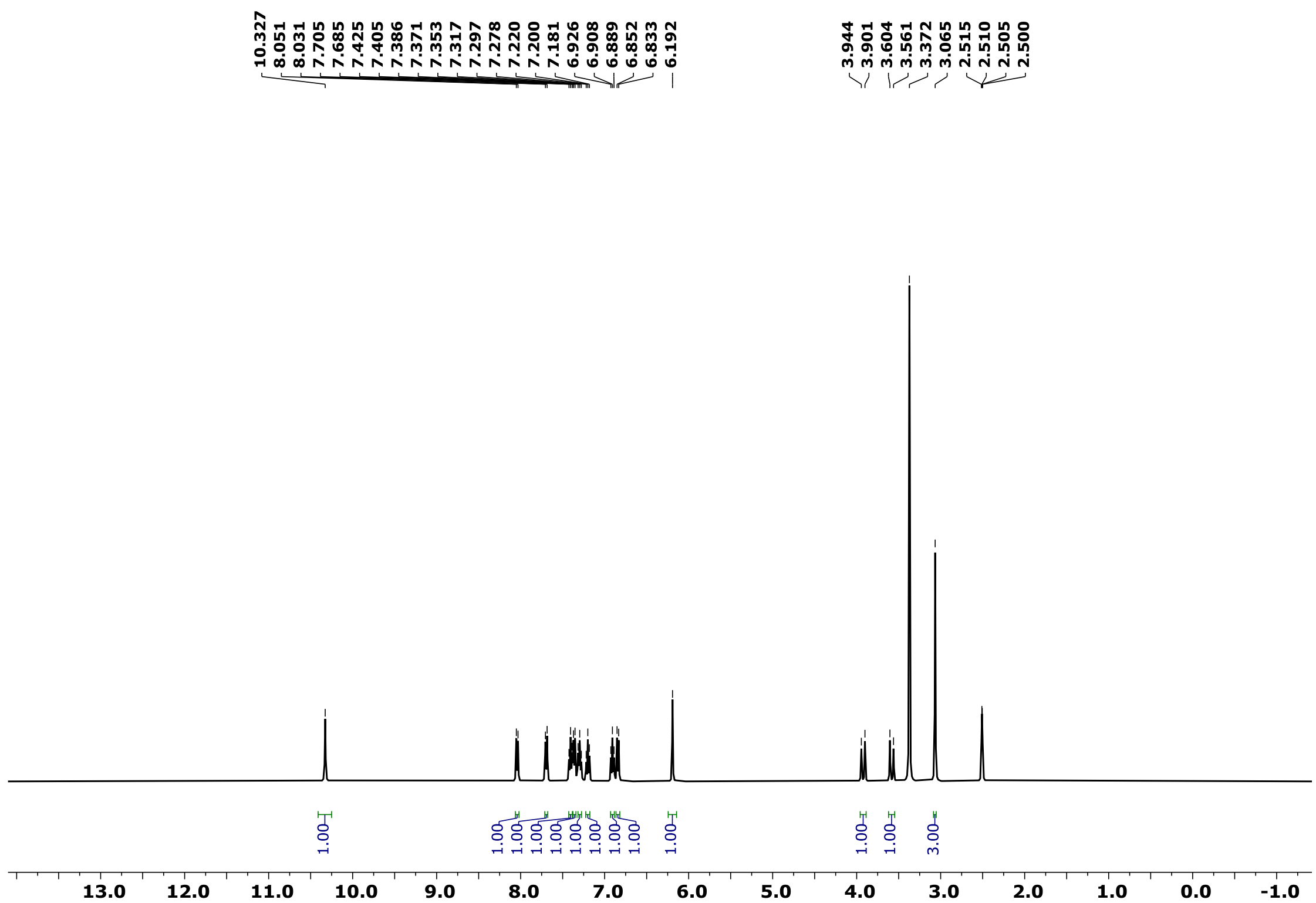

Figure S1. ^{1}H NMR (600 MHz, DMSO-d_6) spectrum of compound (**6a**)

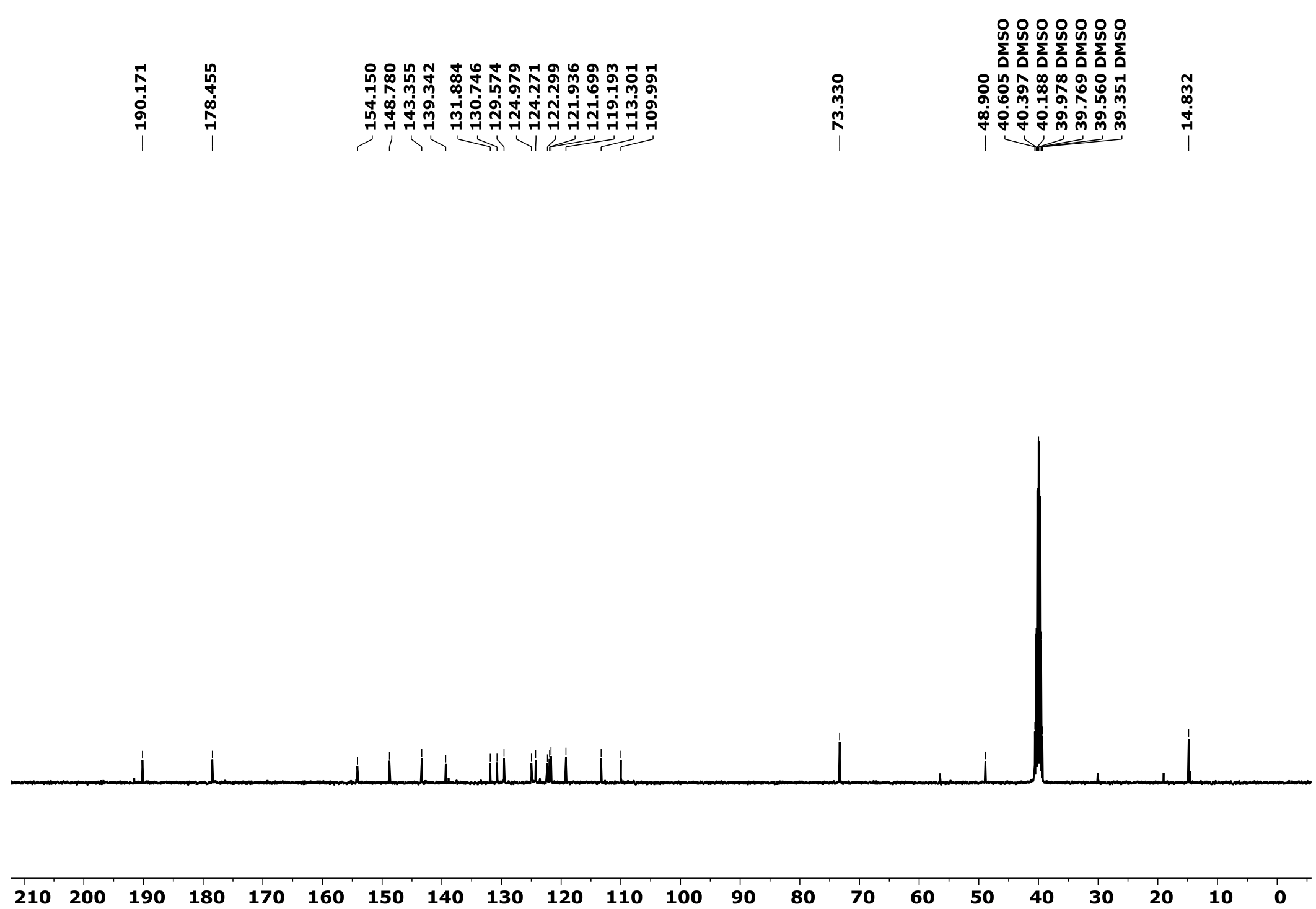

Figure S2. ^{13}C NMR (150 MHz, DMSO-d_6) spectrum of compound (**6a**)

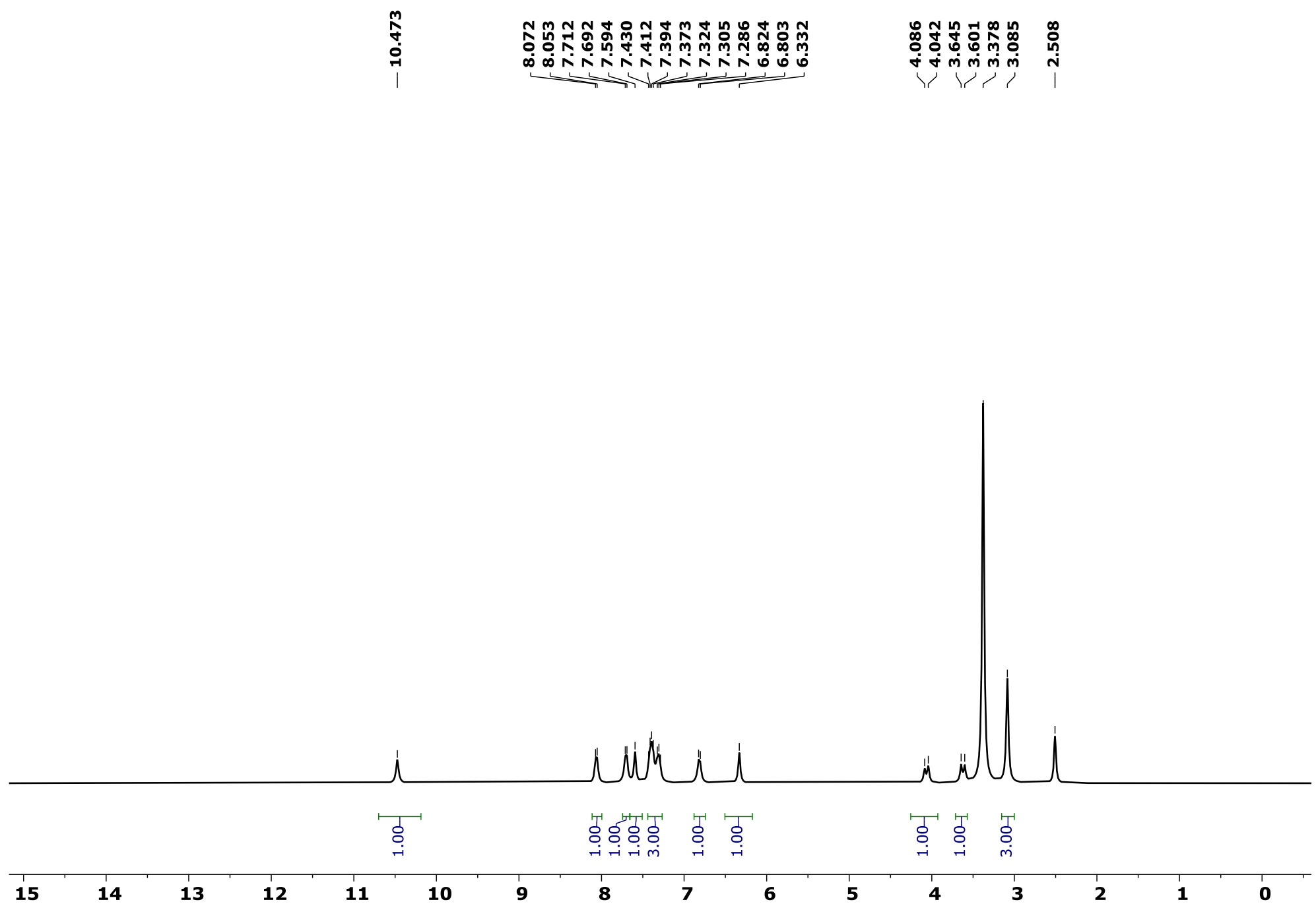

Figure S3. ^{1}H NMR (600 MHz, DMSO-d_6) spectrum of compound (**6b**)

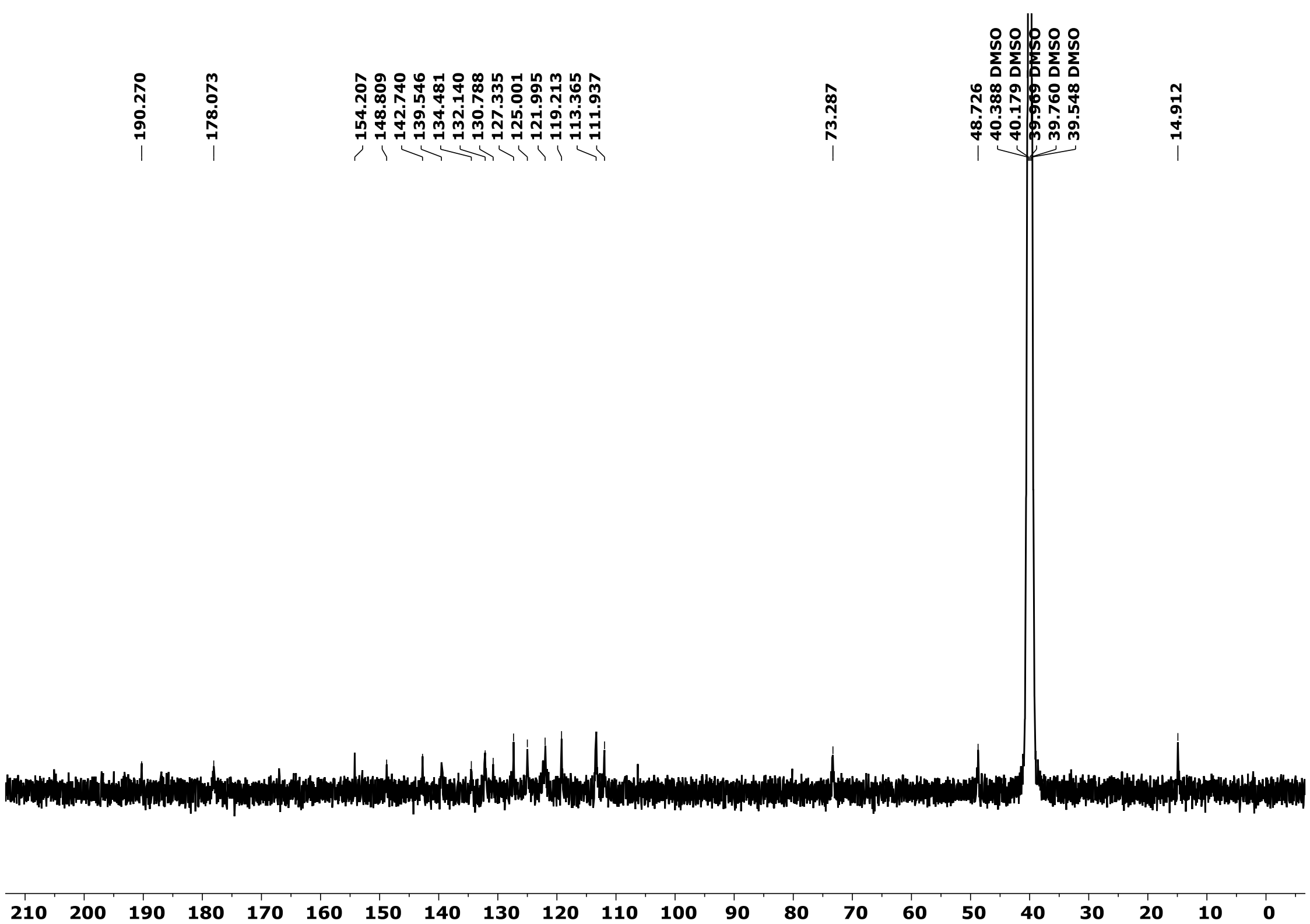

Figure S4. ^{13}C NMR (150 MHz, DMSO-d_6) spectrum of compound (**6b**)

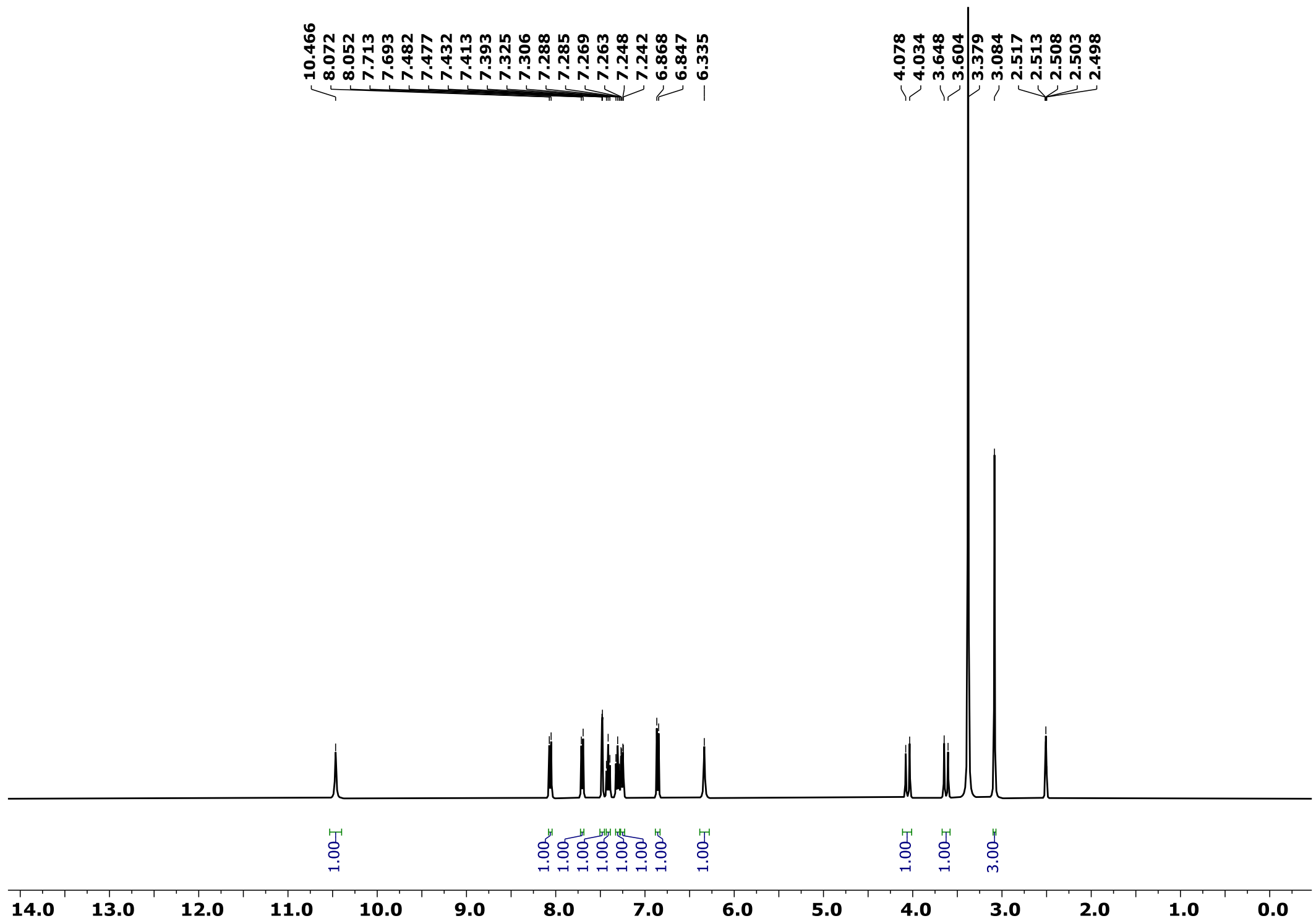

Figure S5. ^{1}H NMR (600 MHz, DMSO-d_6) spectrum of compound (**6c**)

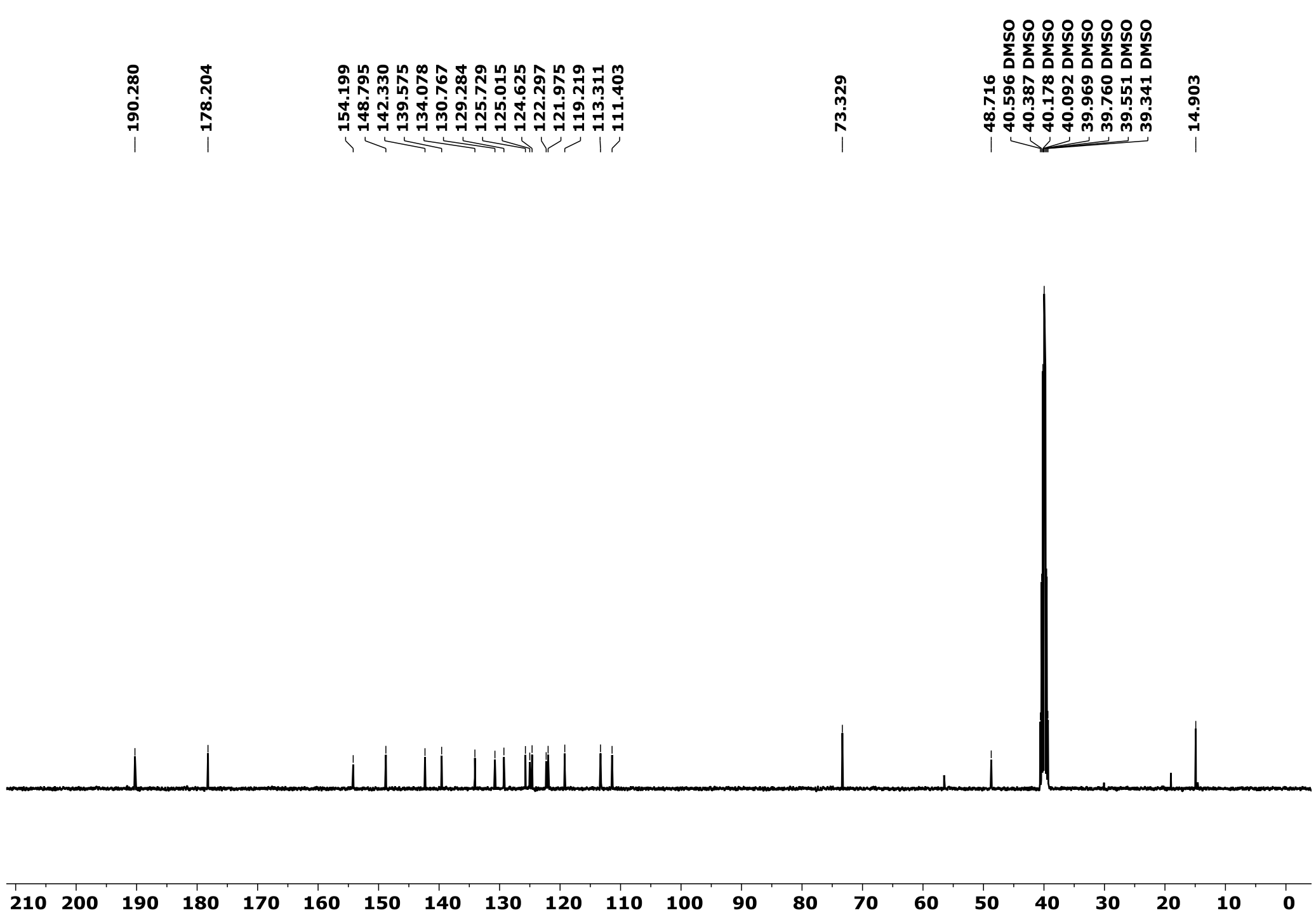

Figure S6. ^{13}C NMR (150 MHz, DMSO-d_6) spectrum of compound (**6c**)

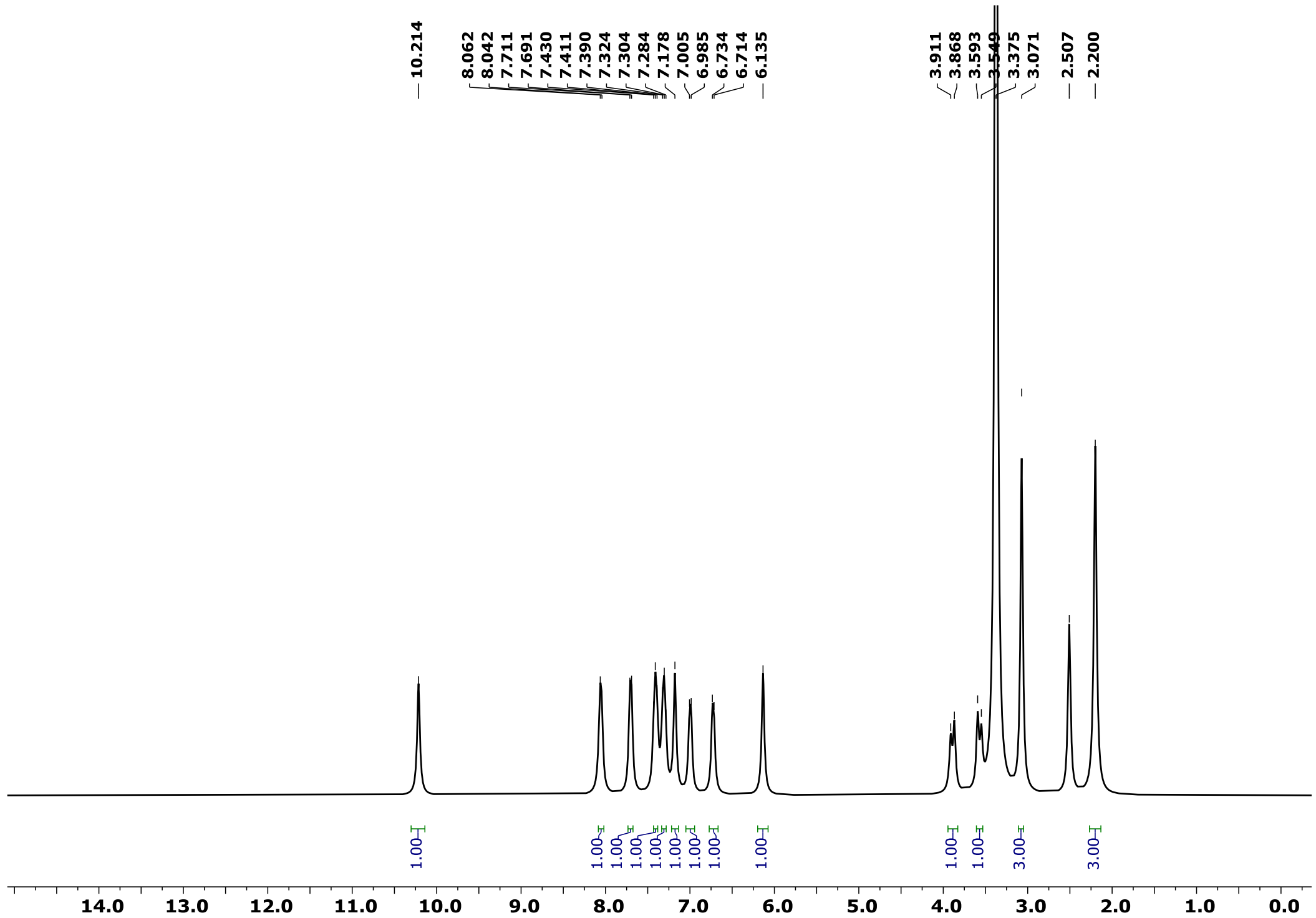

Figure S7. ^{1}H NMR (600 MHz, DMSO-d_6) spectrum of compound (**6d**)

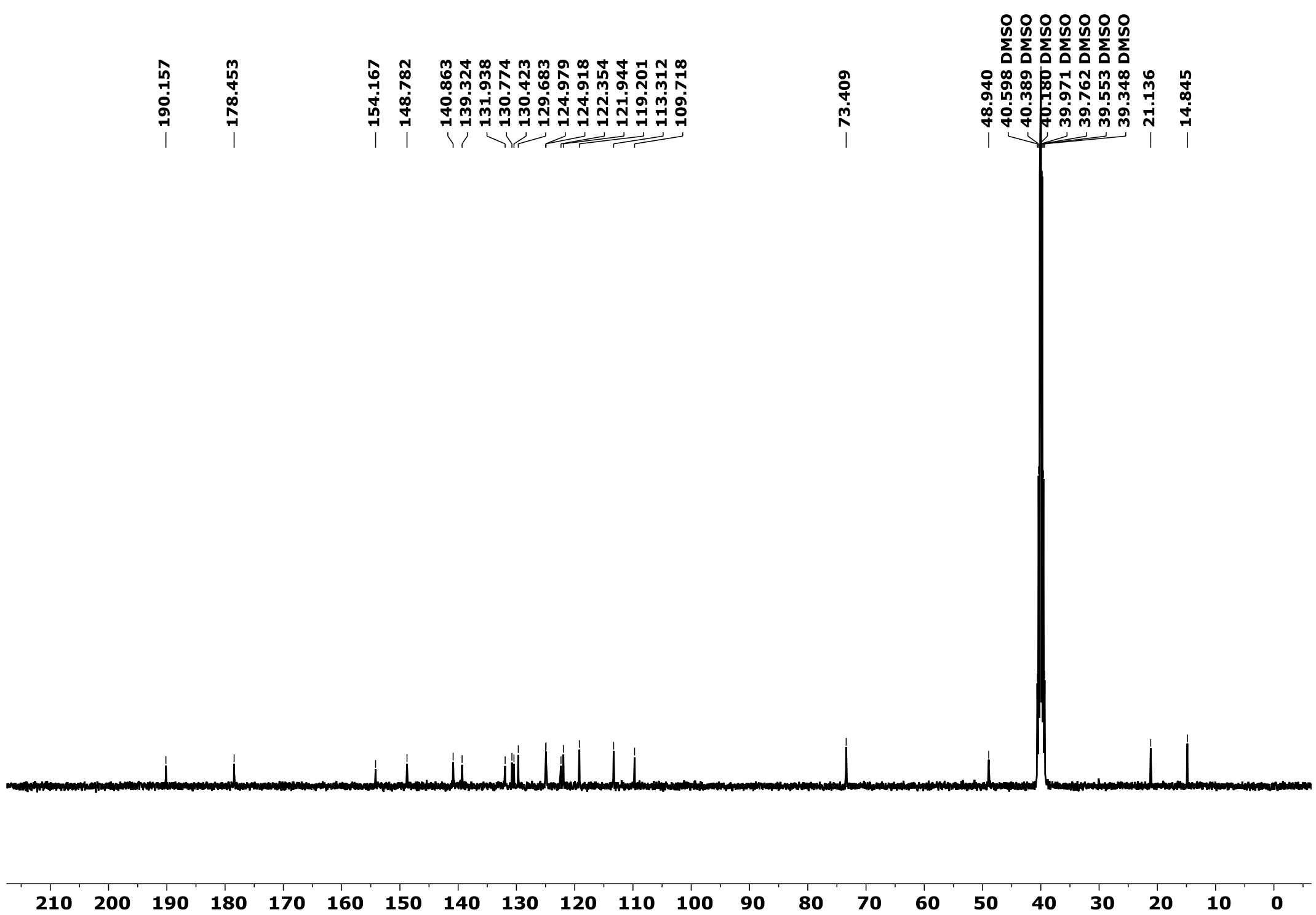

Figure S8. ^{13}C NMR (150 MHz, DMSO-d_6) spectrum of compound (**6d**)

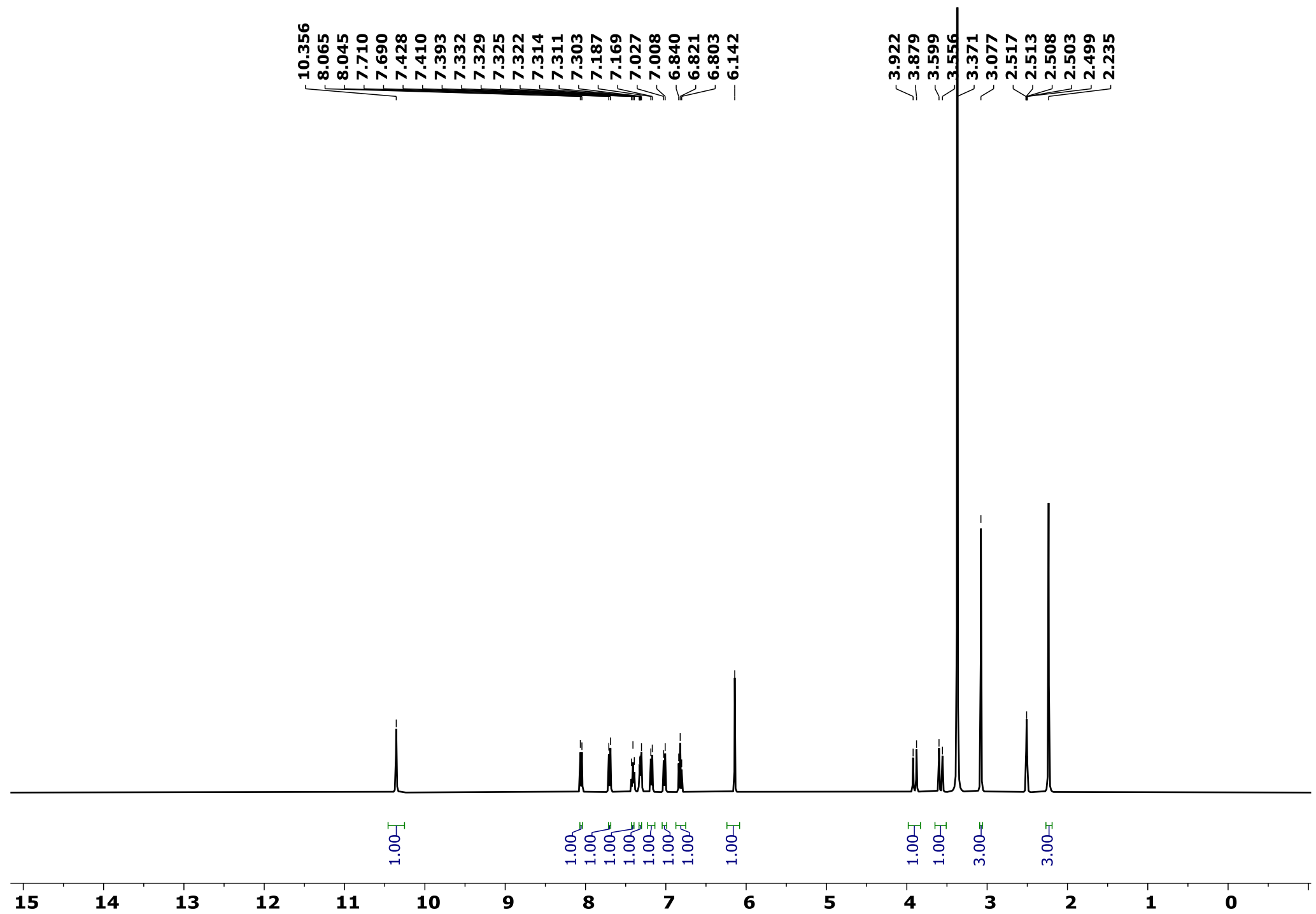

Figure S9. ^{1}H NMR (600 MHz, DMSO-d_6) spectrum of compound (**6e**)

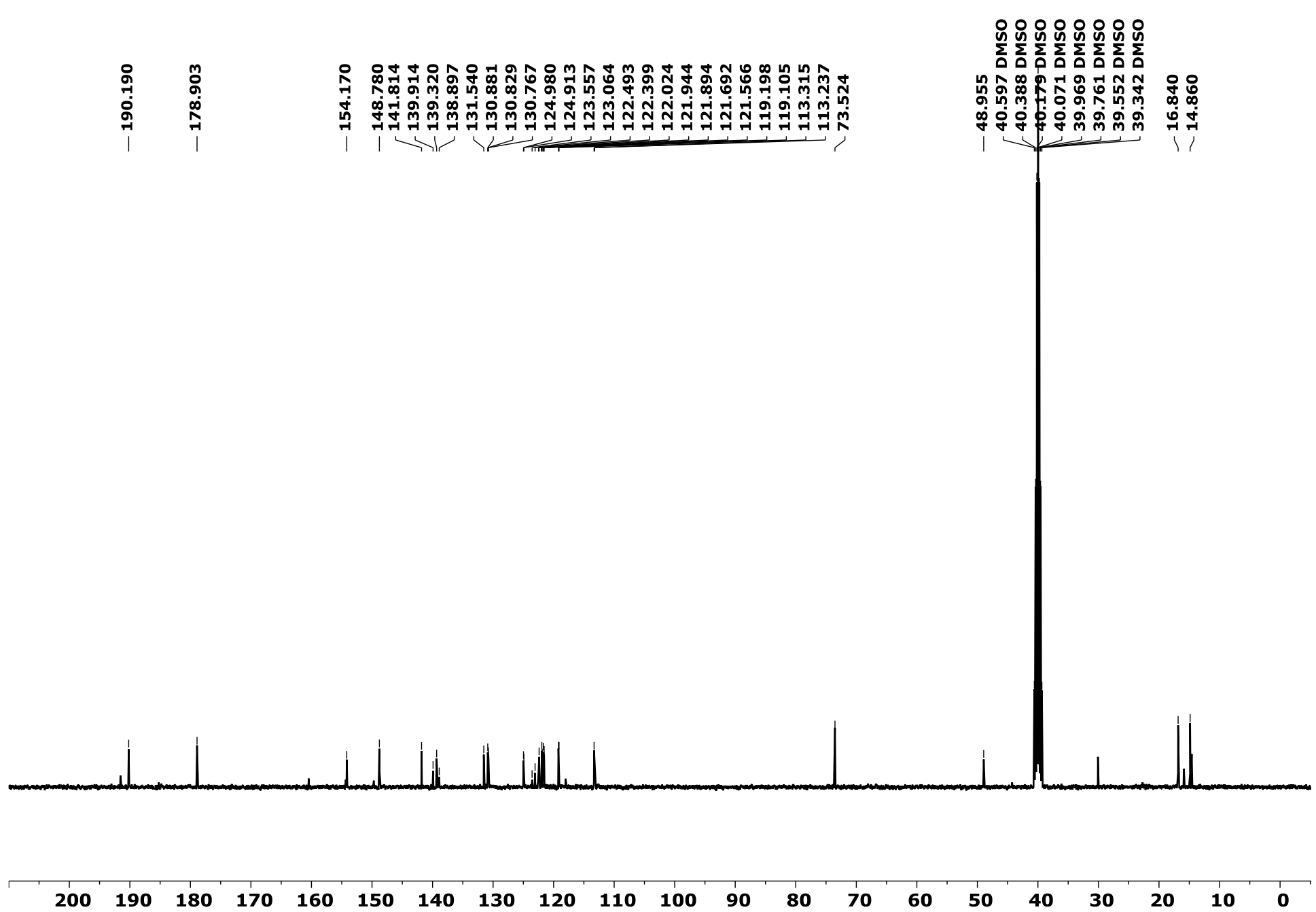

Figure S10. ^{13}C NMR (150 MHz, DMSO-d_6) spectrum of compound (**6e**)

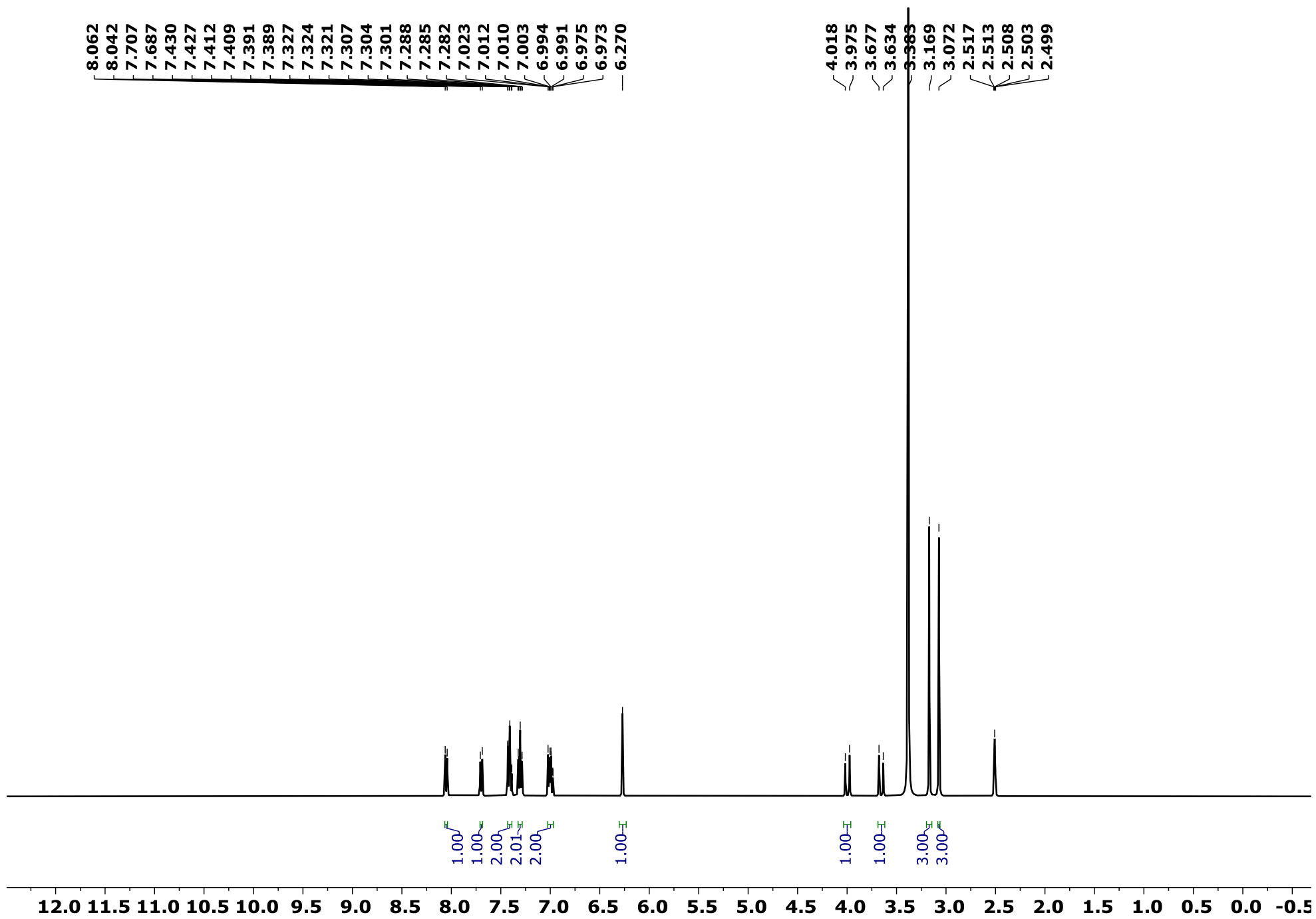

Figure S11. ^{1}H NMR (600 MHz, DMSO-d_6) spectrum of compound (**8a**)

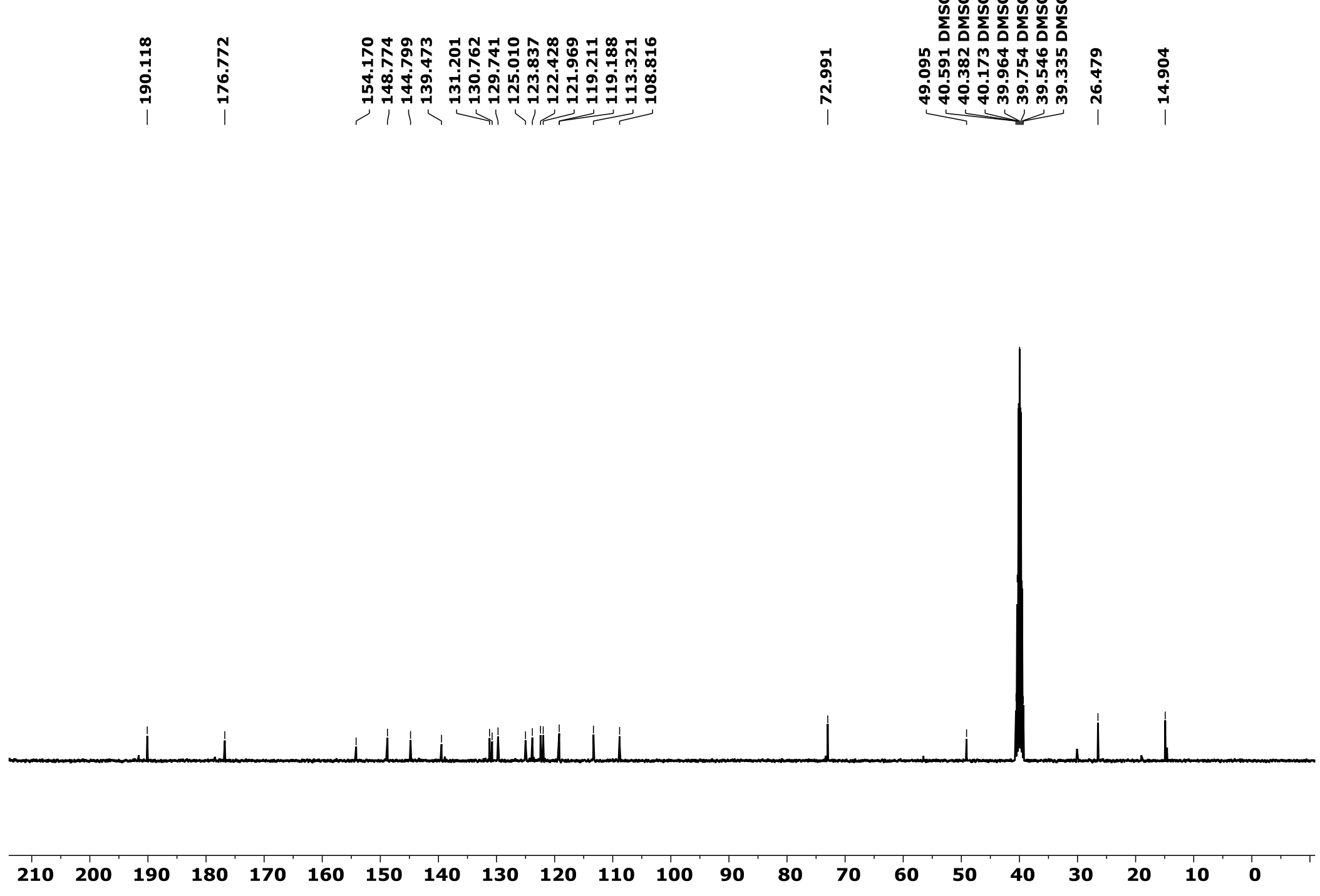

Figure S12. ^{13}C NMR (150 MHz, DMSO-d_6) spectrum of compound (**8a**)

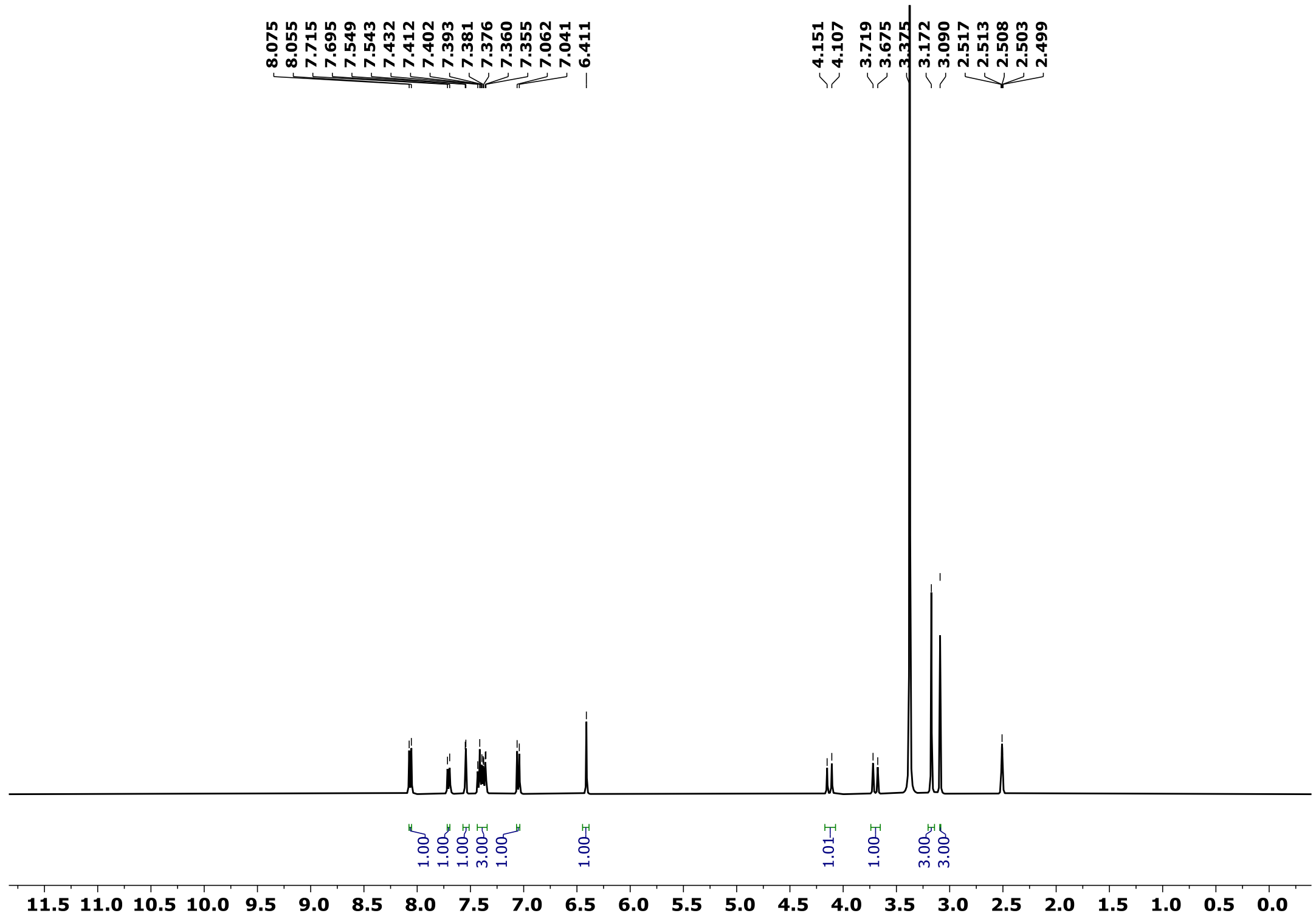

Figure S13. ^{1}H NMR (600 MHz, DMSO-d_6) spectrum of compound (**8b**)

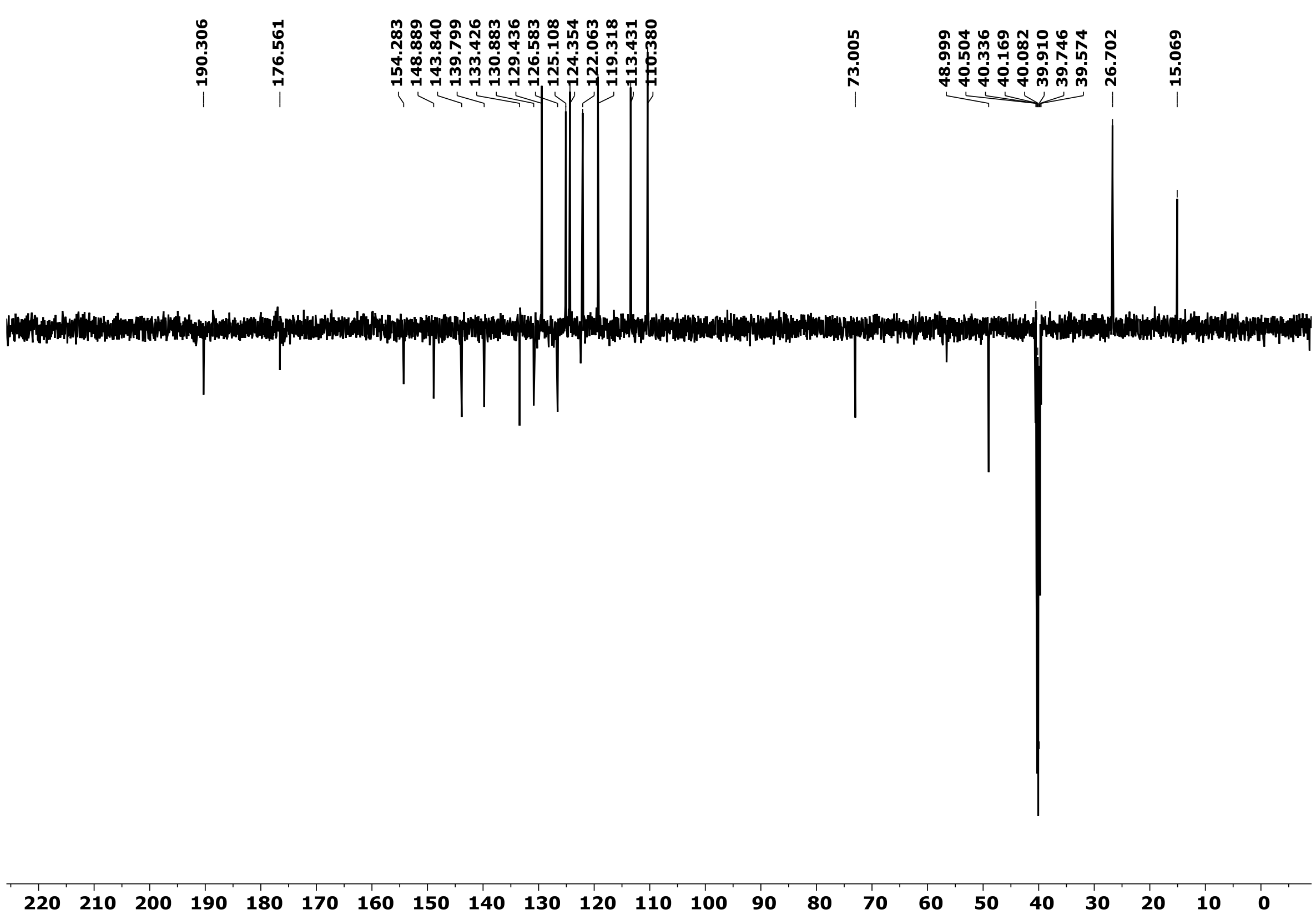

Figure S14. ^{13}C NMR (150 MHz, DMSO-d_6) spectrum of compound (**8b**)

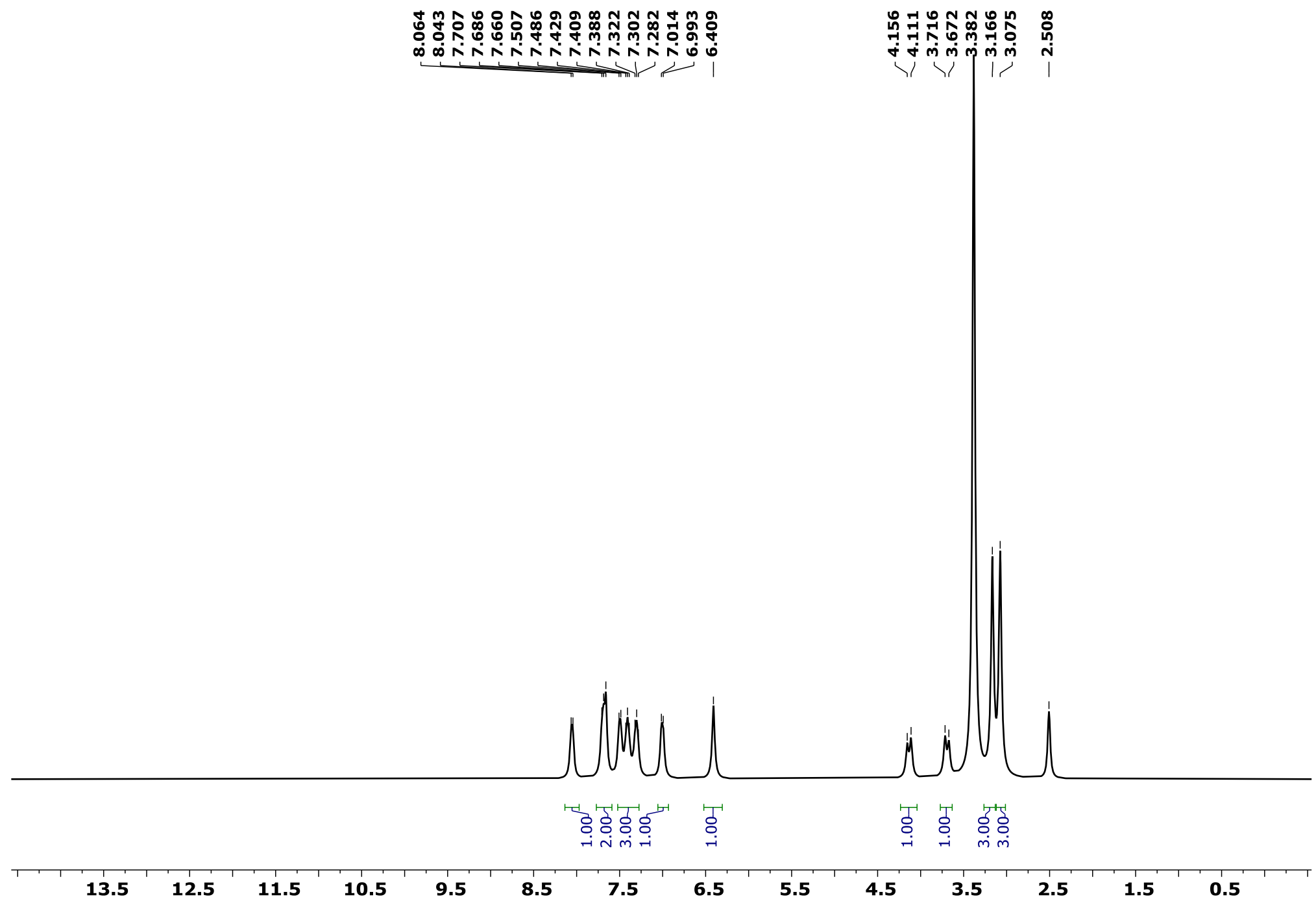

Figure S15. ^{1}H NMR (600 MHz, DMSO-d_6) spectrum of compound (**8c**)

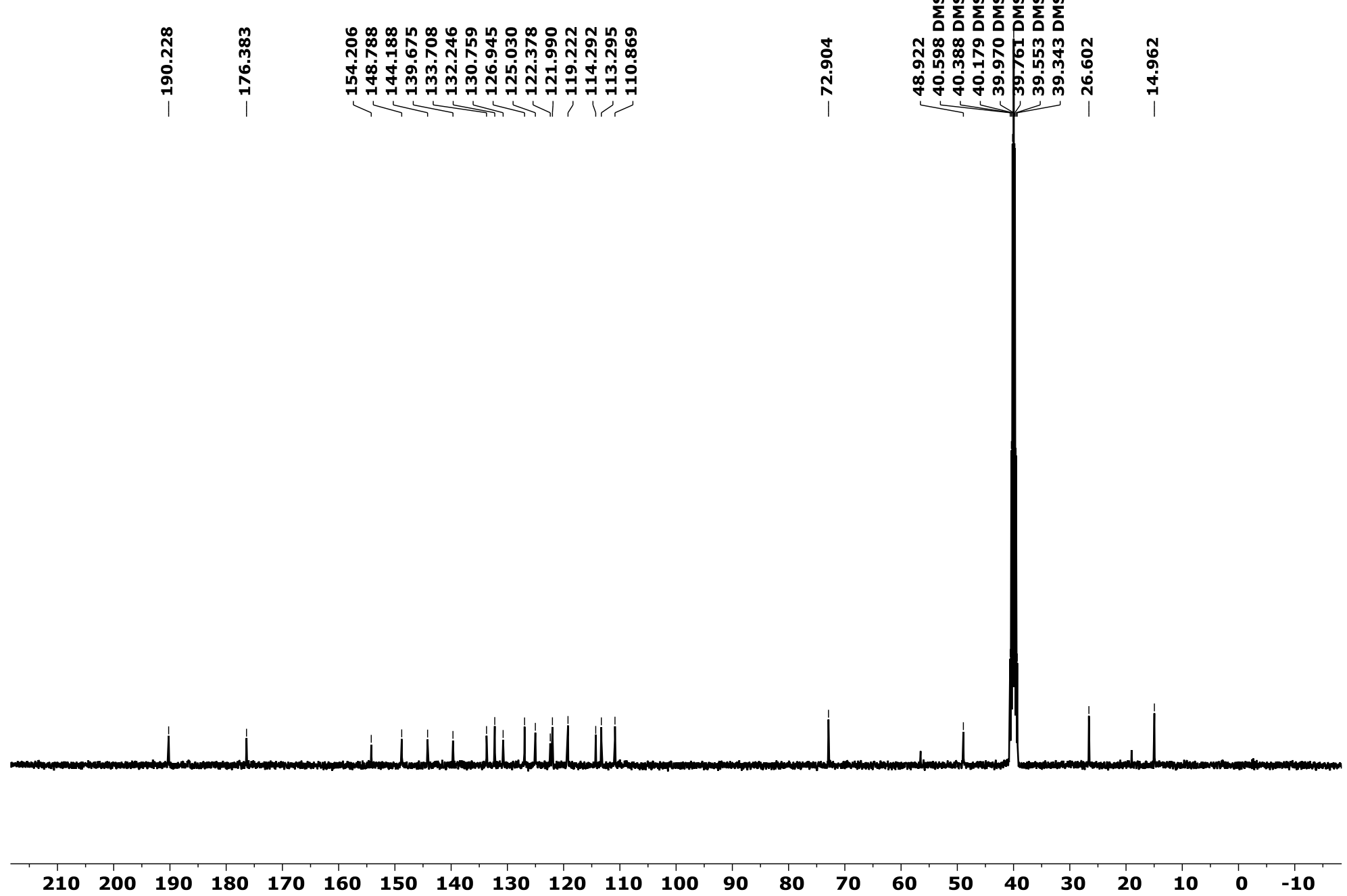

Figure S16. ^{13}C NMR (150 MHz, DMSO-d_6) spectrum of compound (**8c**)

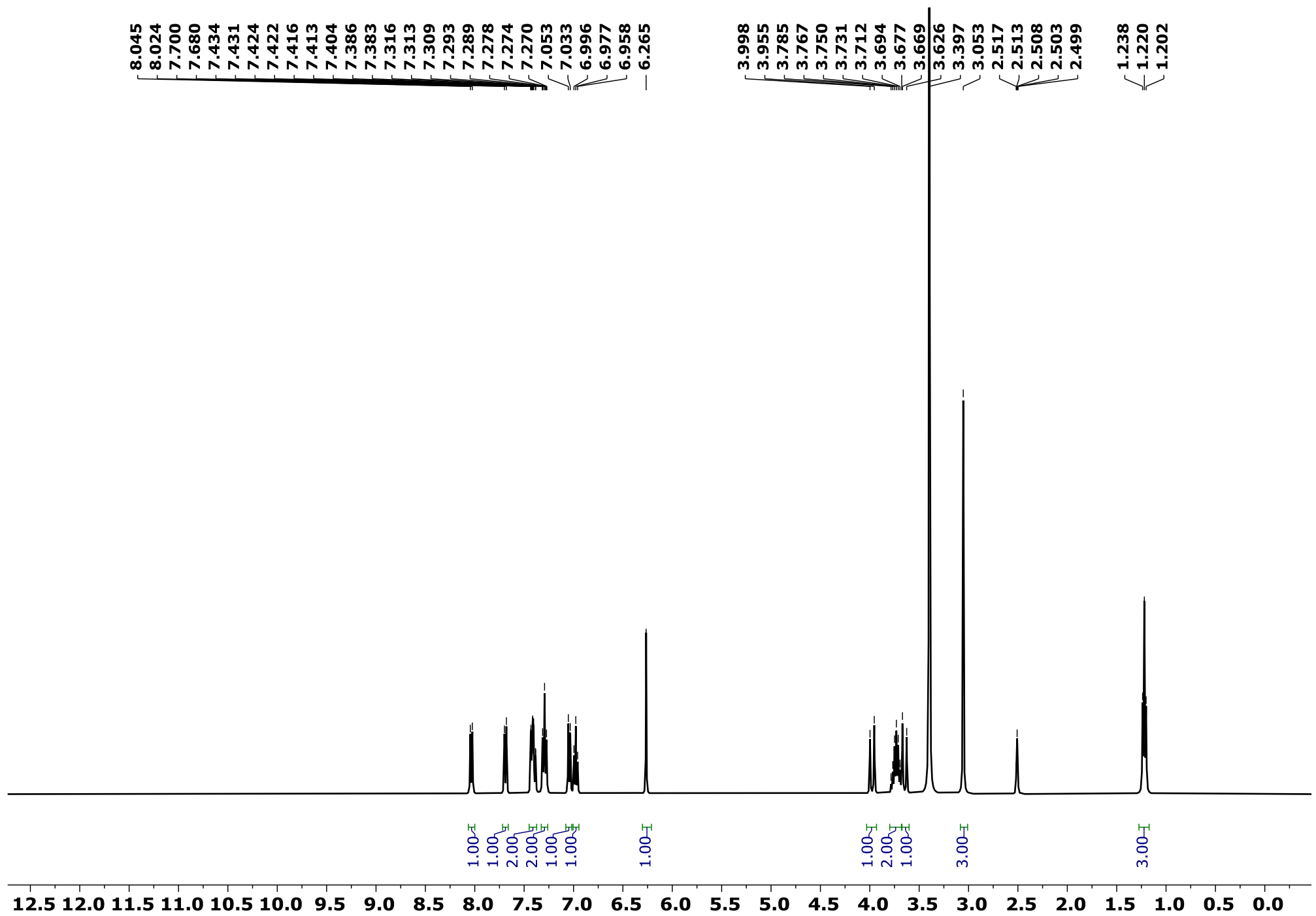

Figure S17. ^{1}H NMR (600 MHz, DMSO-d_6) spectrum of compound (**8d**)

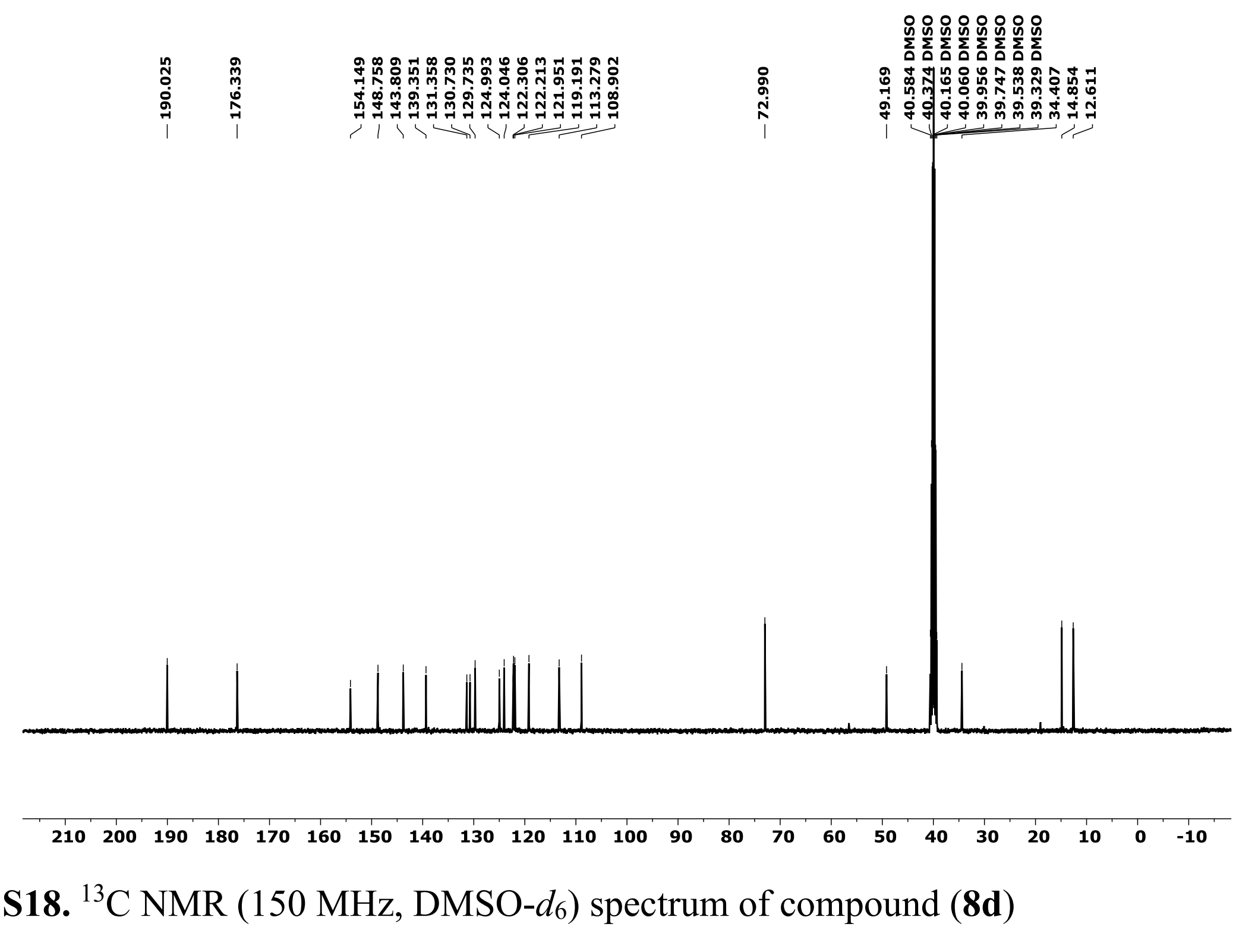

Figure S18. ^{13}C NMR (150 MHz, DMSO-d_6) spectrum of compound (**8d**)

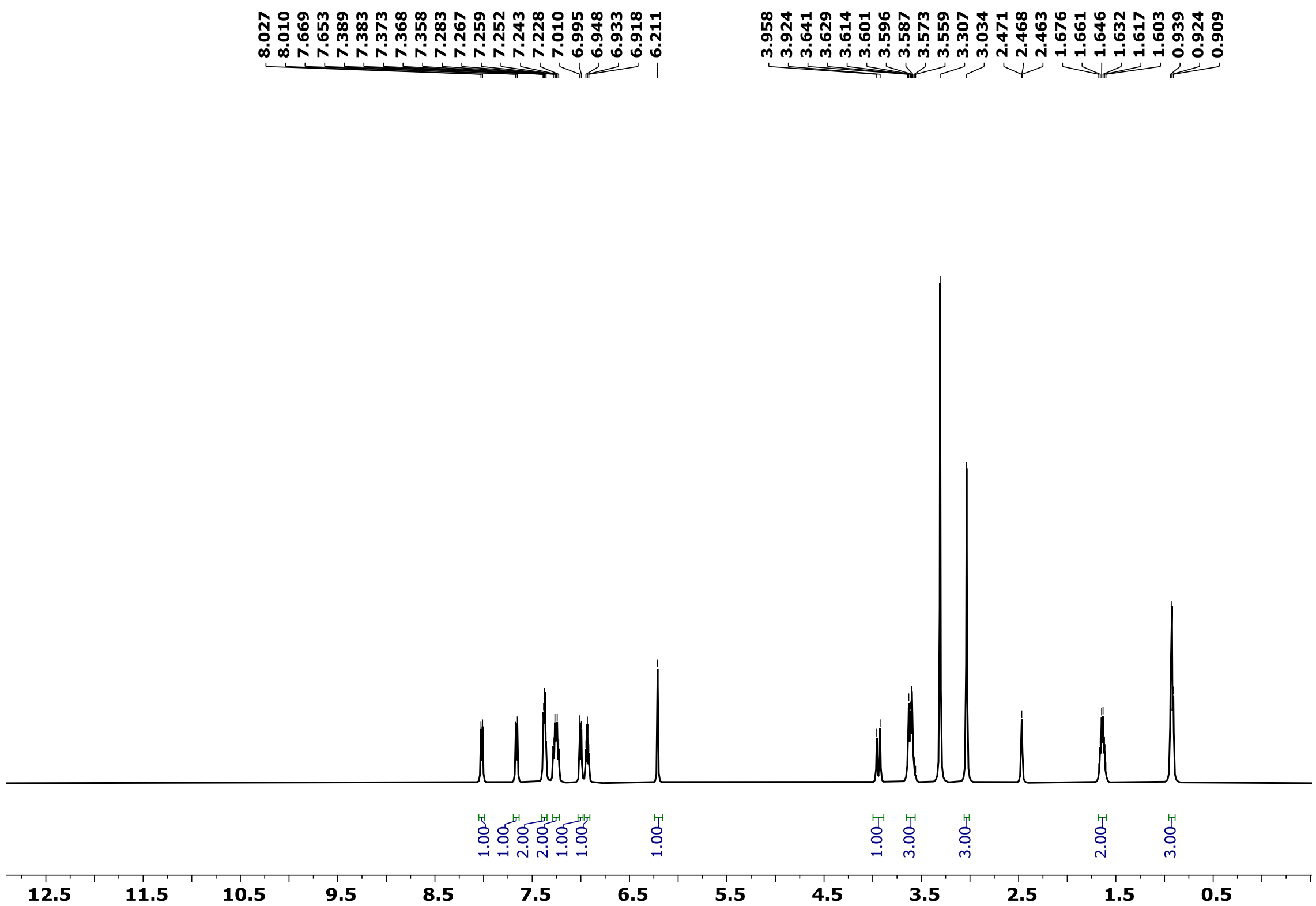

Figure S19. ^{1}H NMR (600 MHz, DMSO-d_6) spectrum of compound (**8e**)

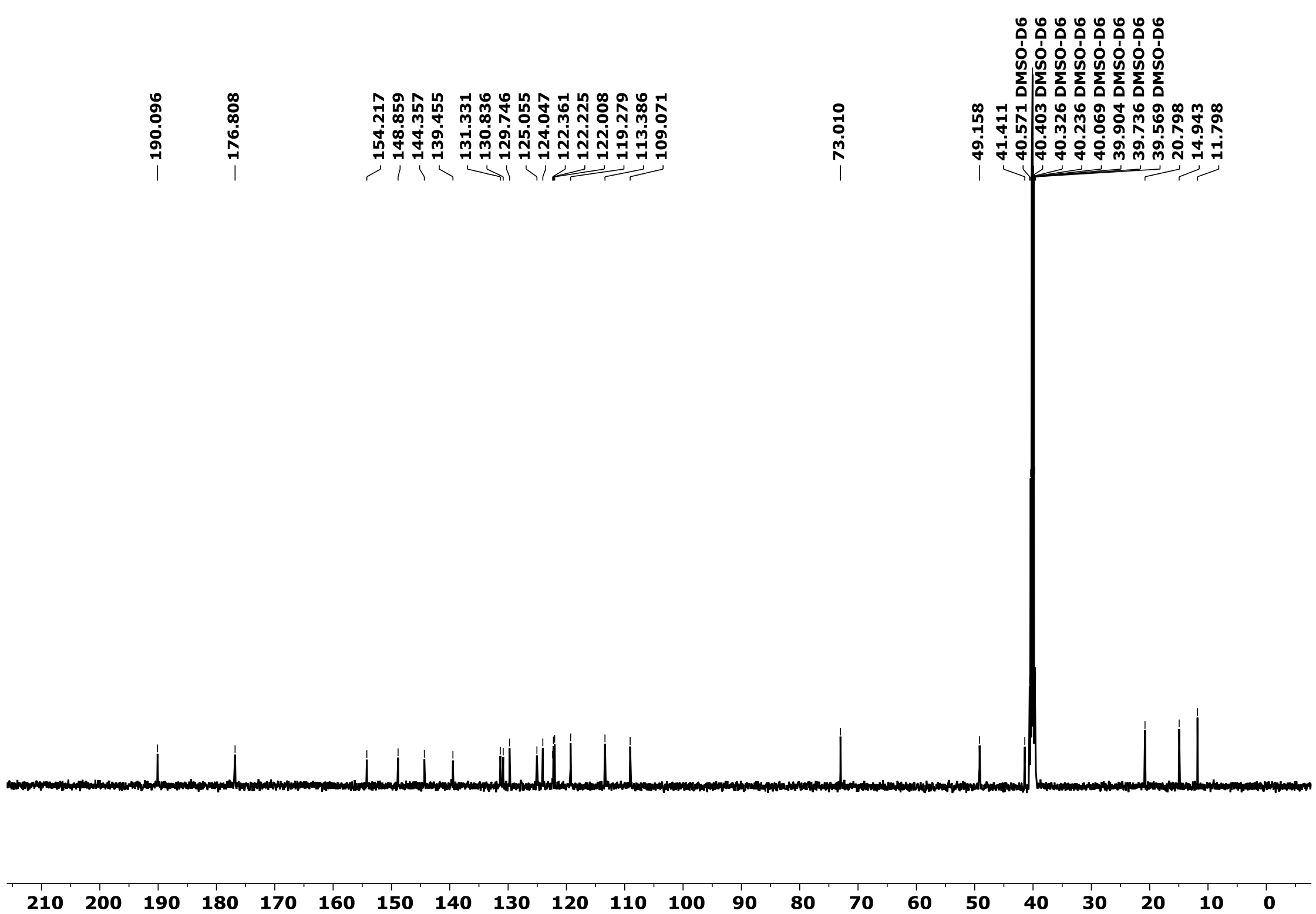

Figure S20. ^{13}C NMR (150 MHz, DMSO-d_6) spectrum of compound (**8e**)

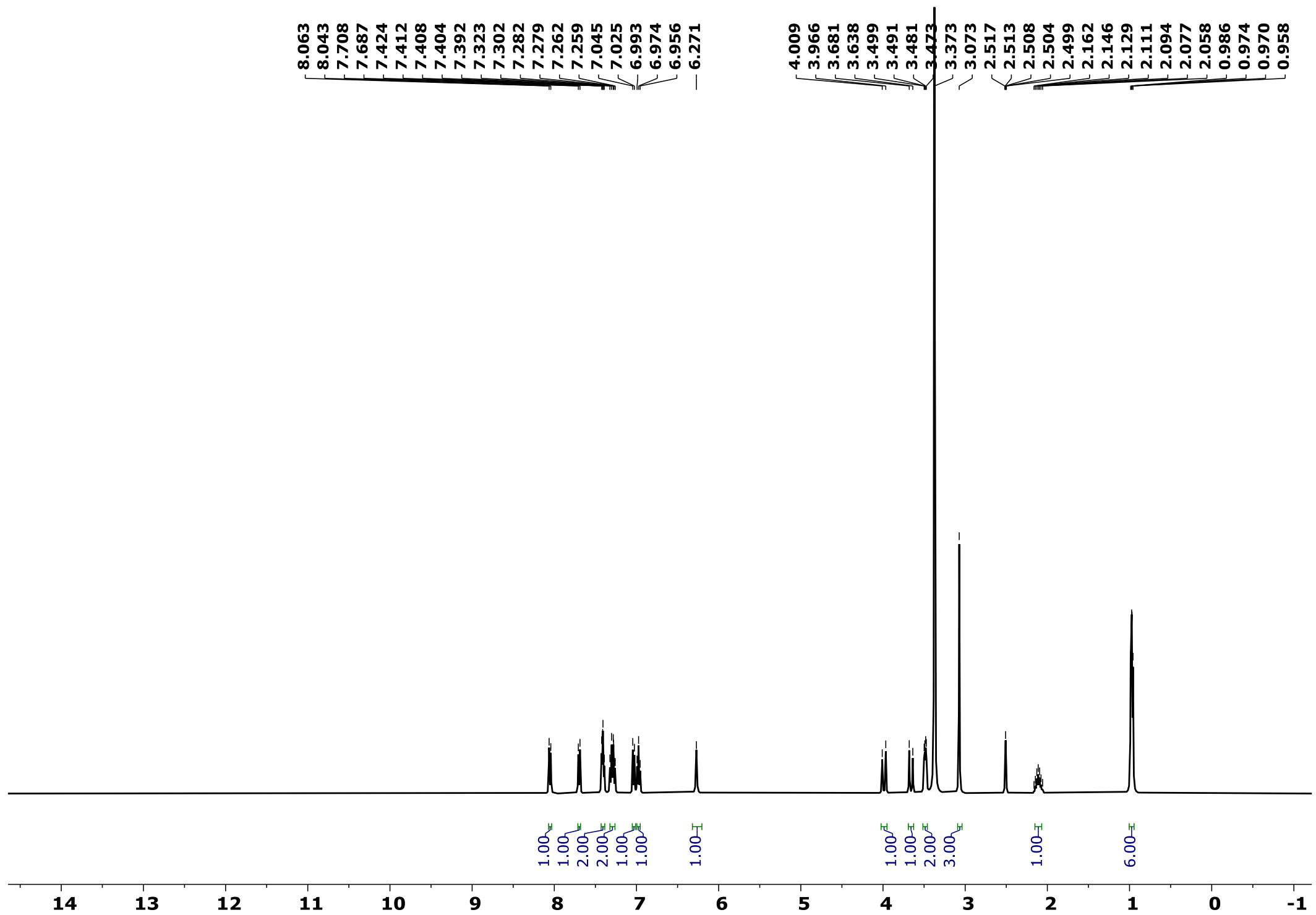

Figure S21. ^{1}H NMR (600 MHz, DMSO-d_6) spectrum of compound (**8f**)

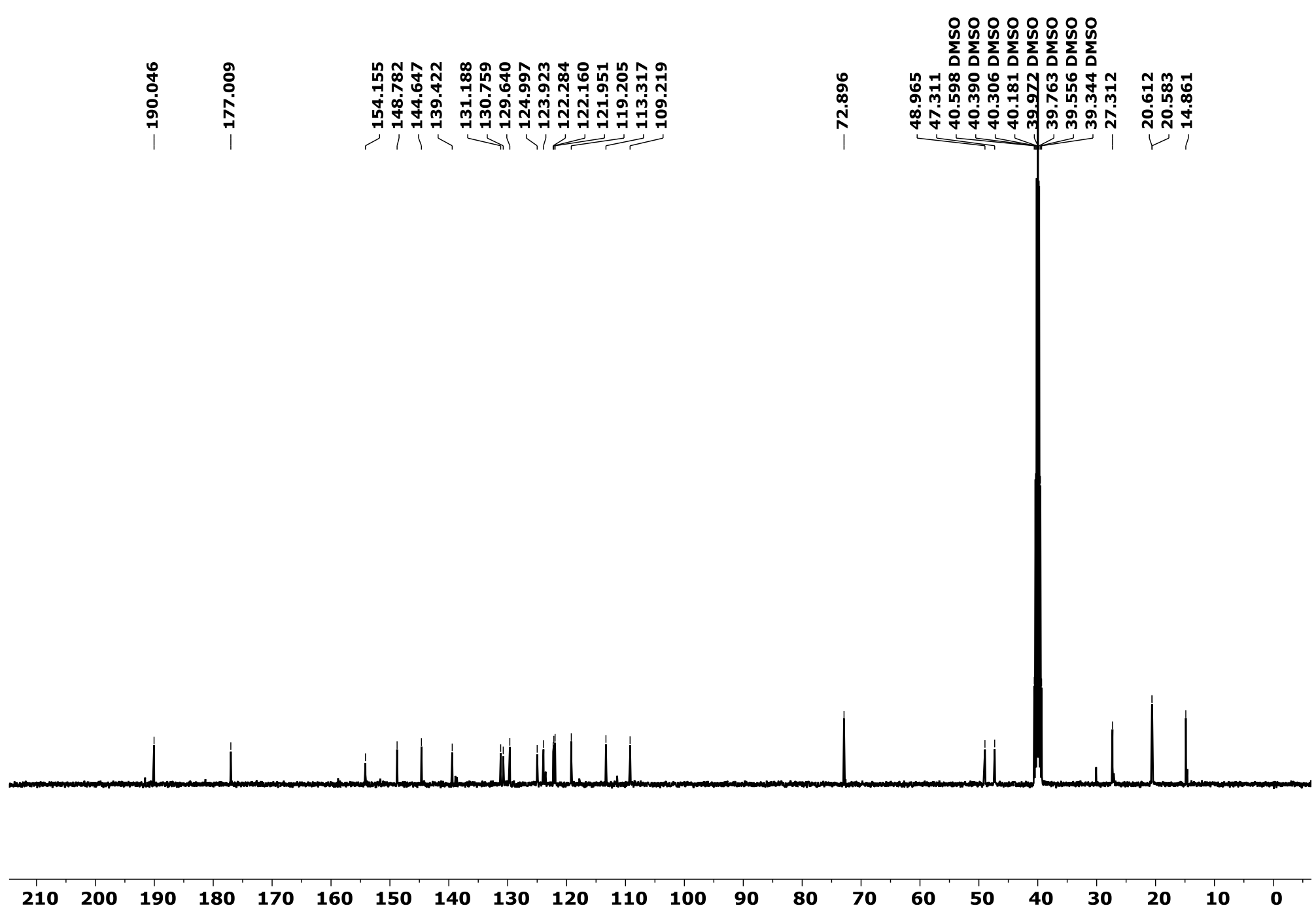

Figure S22. ^{13}C NMR (150 MHz, DMSO-d_6) spectrum of compound (**8f**)

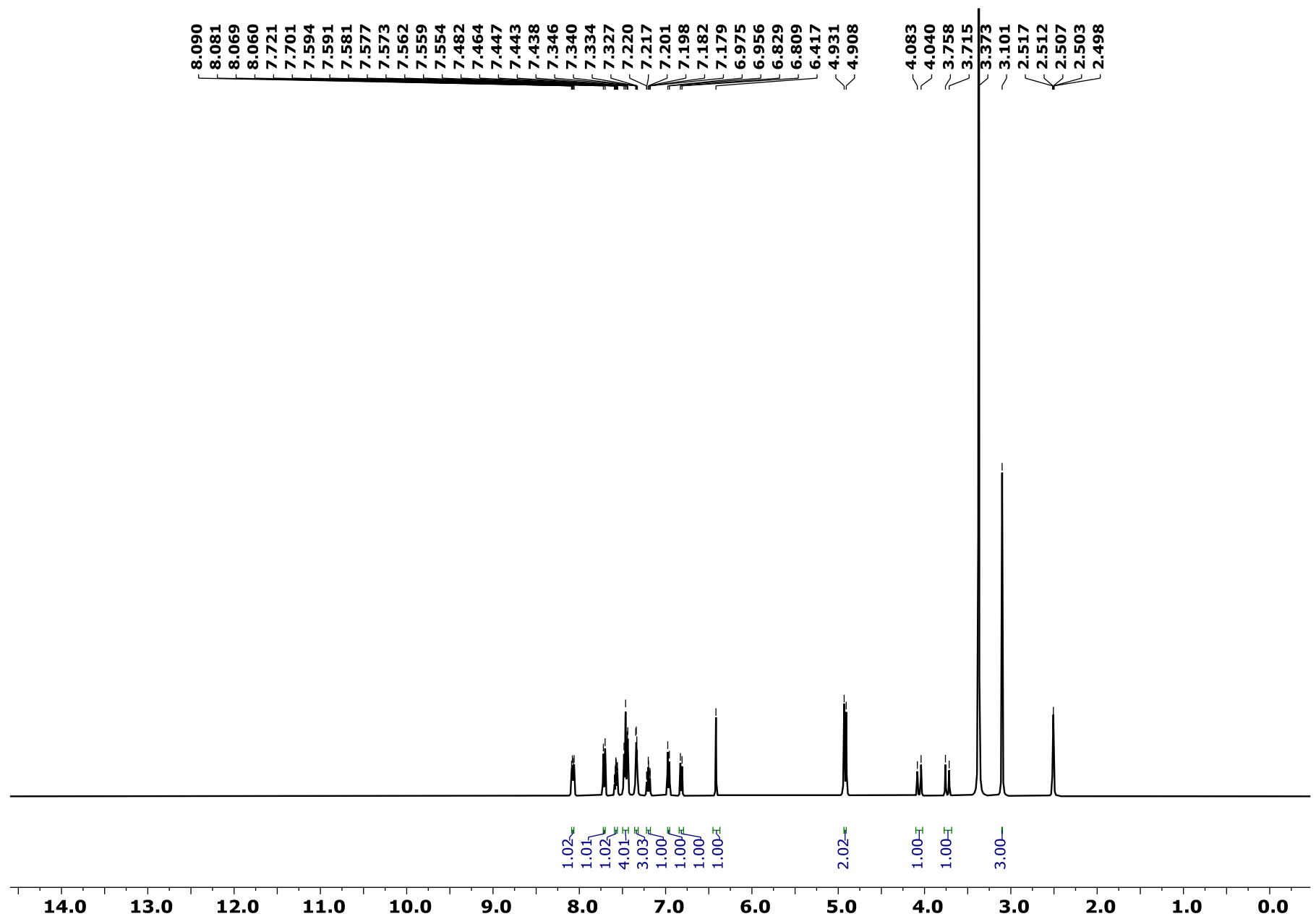

Figure S23. ^{1}H NMR (600 MHz, DMSO-d_6) spectrum of compound (**8g**)

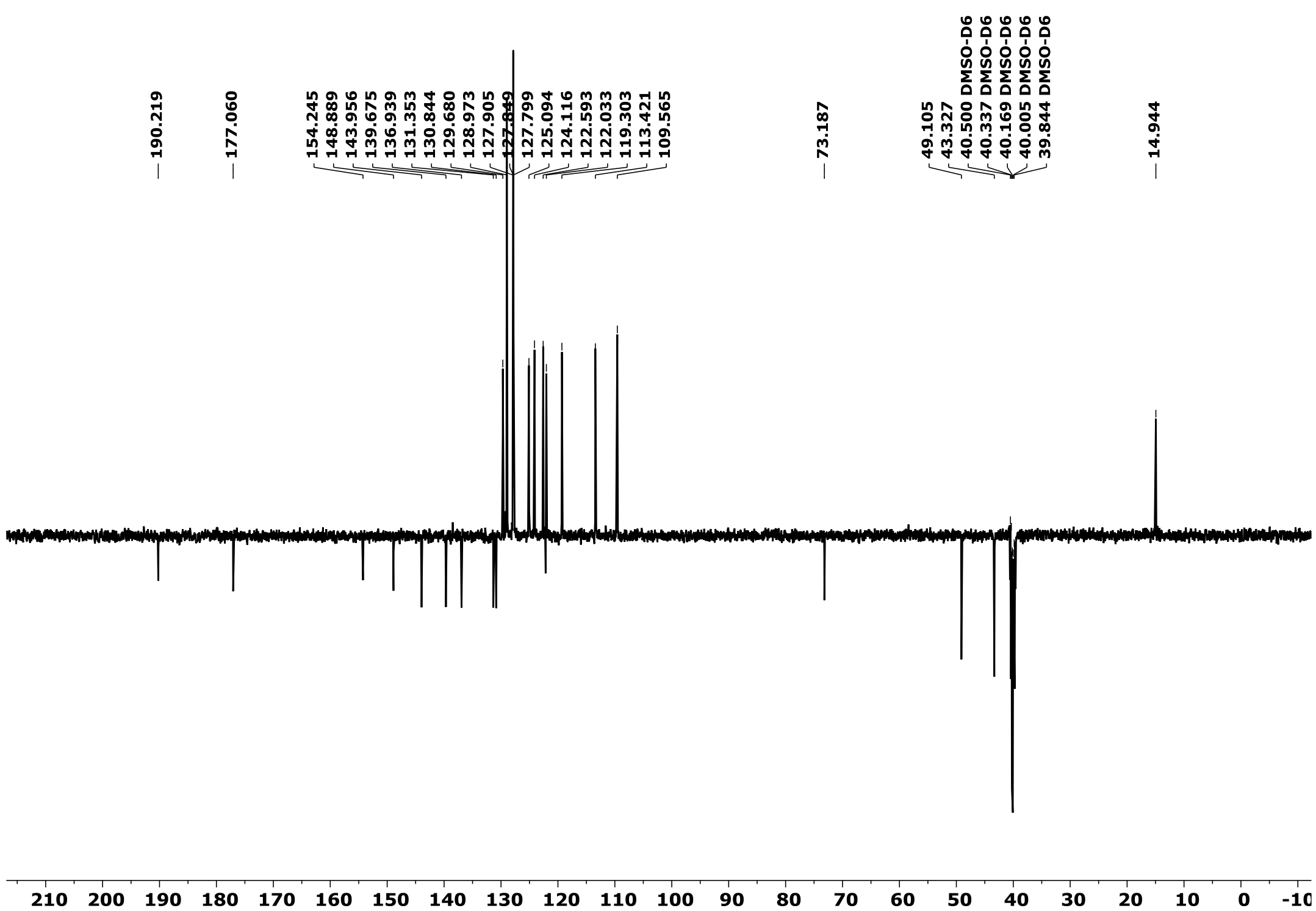

Figure S24. DEPTQ (150 MHz, DMSO-d_6) spectrum of compound (**8g**)

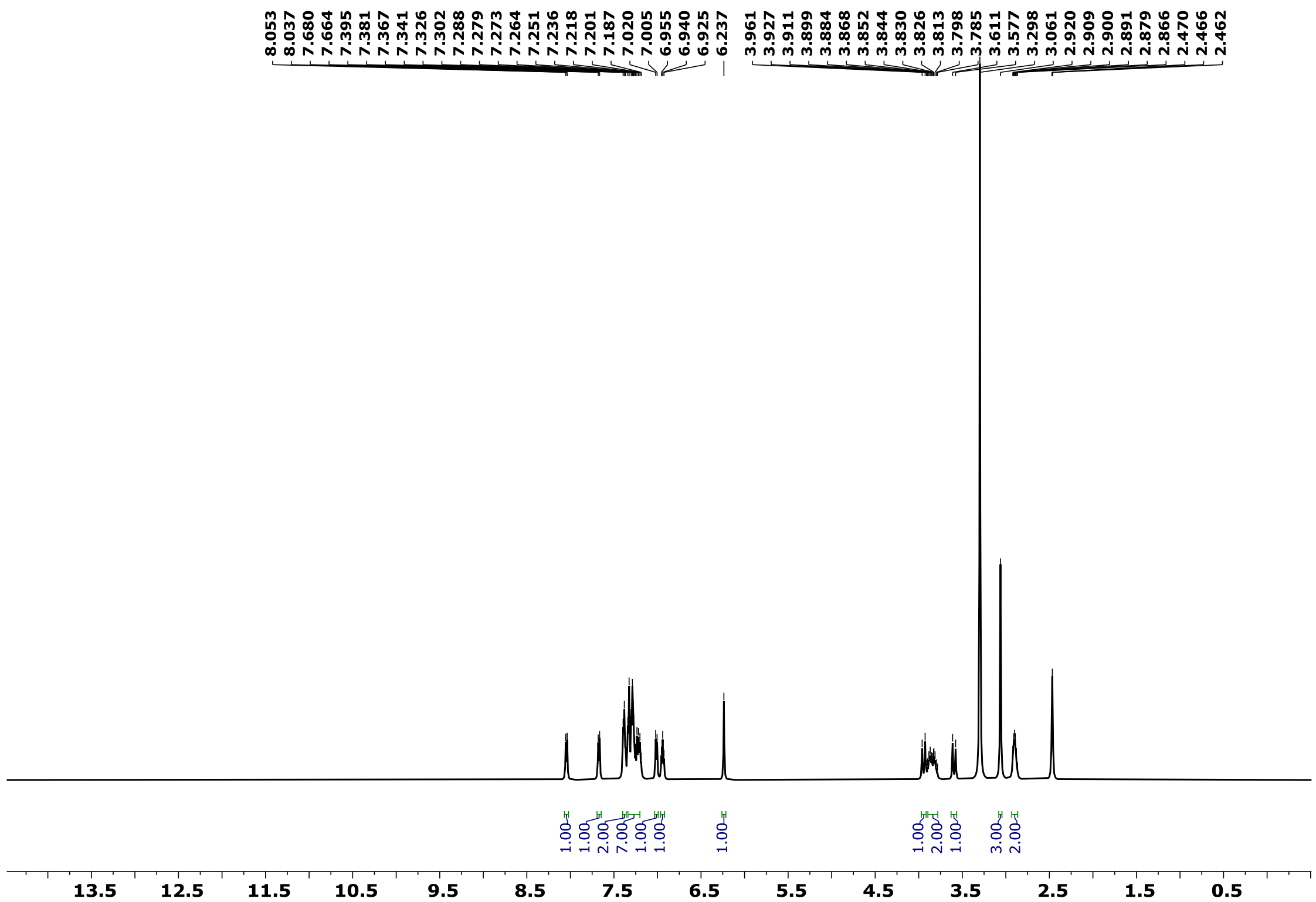

Figure S25. ^{1}H NMR (600 MHz, DMSO-d_6) spectrum of compound (**8h**)

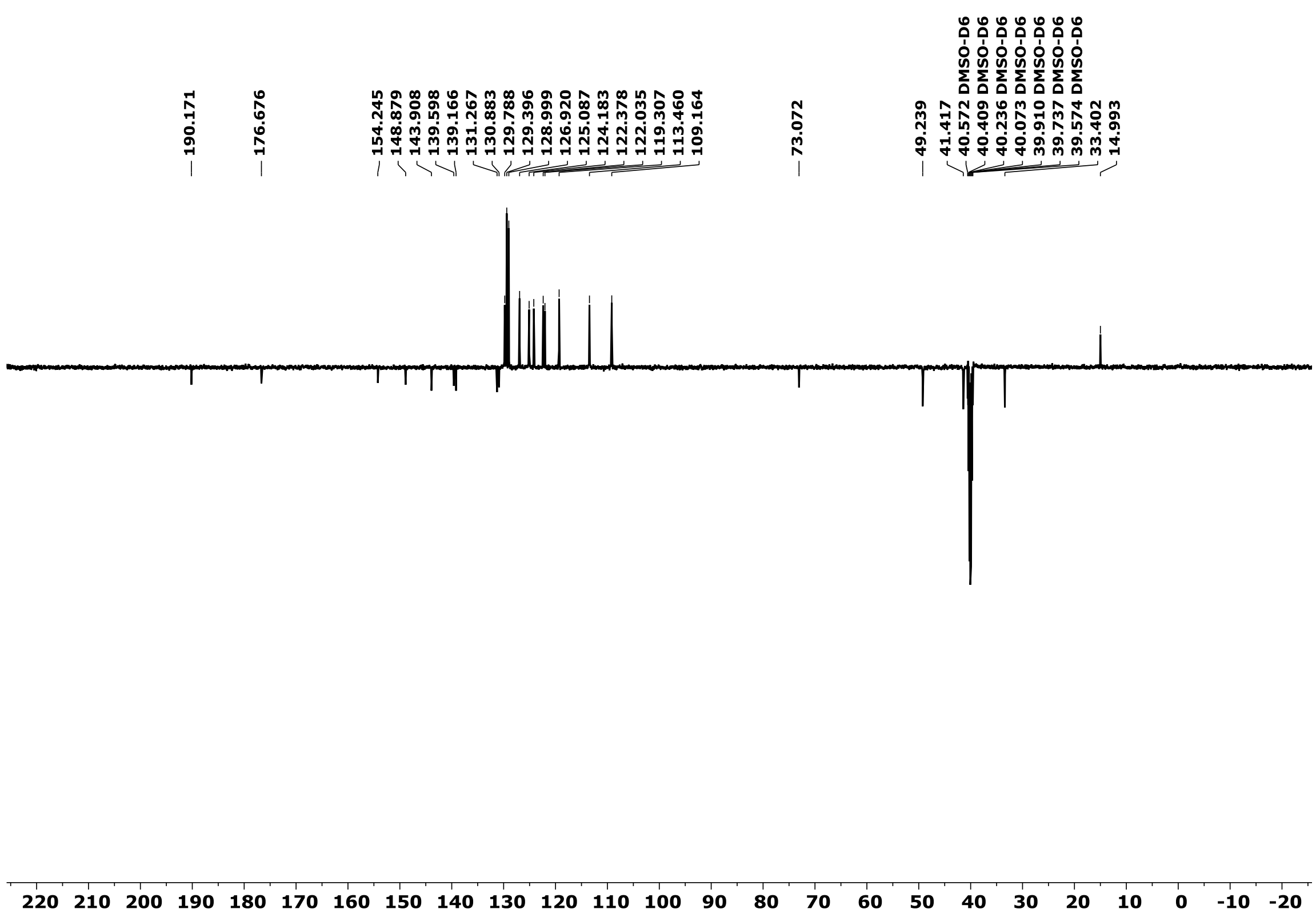

Figure S26. ^{13}C NMR (150 MHz, DMSO-d_6) spectrum of compound (**8h**)

Operator ID:	Tanta Univeristy
Company name:	Thermo Fisher
Method filename:	C:\new calibration 24072023\CHNS_24072023.m
Method name:	CHNS Flash smart
Analysed:	14/01/2025 10:32
Elemental Analyser method:	
Sampler method:	
Sample ID:	6c (# 2)
Chromatogram filename:	6c.dat
Sample weight:	1.52

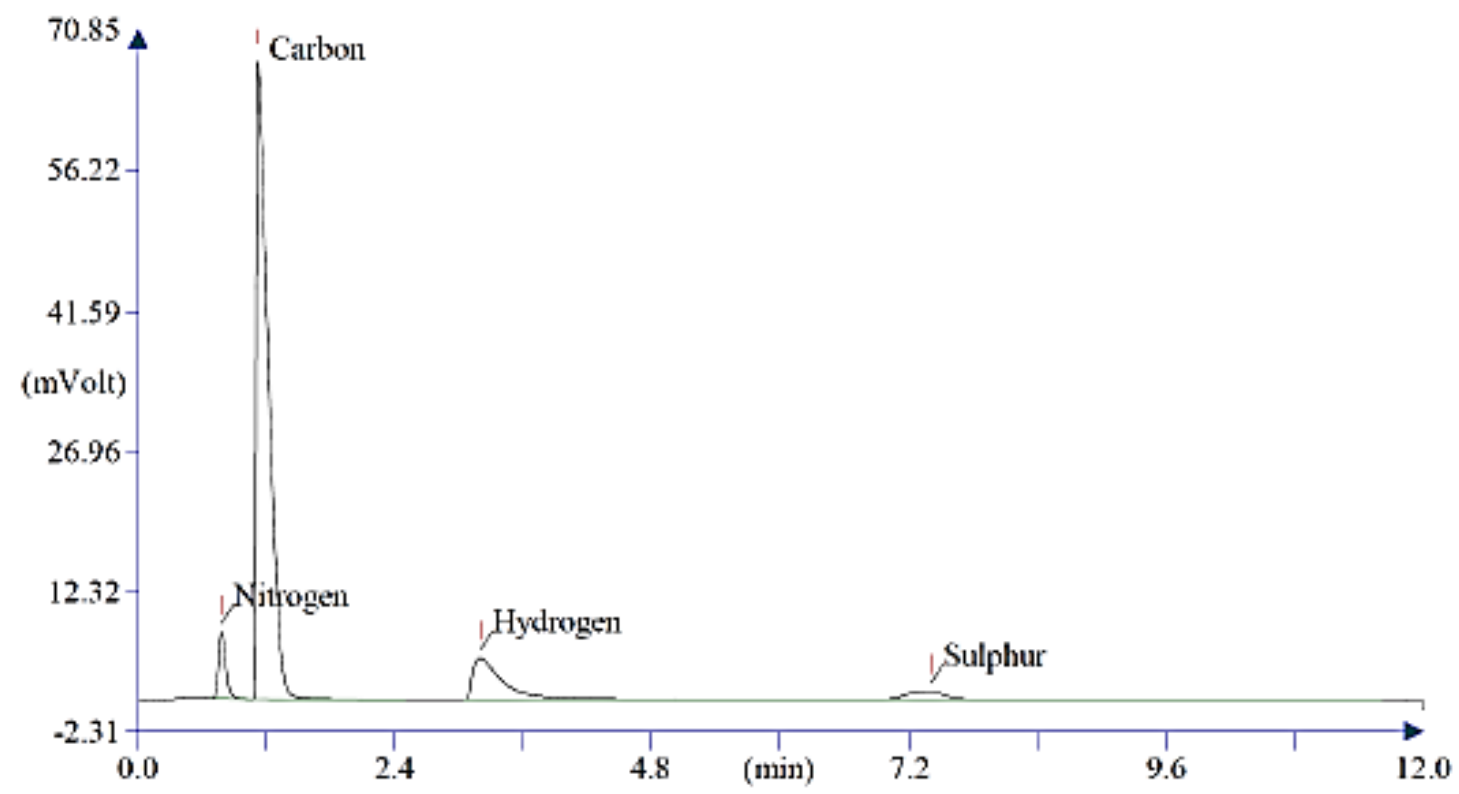

Element Name	%	Ret.Time	Area
Nitrogen	9.2401	0.79	387800
Carbon	52.8518	1.12	5364400
Hydrogen	3.1017	3.23	1020300
Sulphur	7.0013	7.46	287800
Totals	72.1949		7060300

Figure S27. Original instrument-generated CHNS elemental analysis report for compound **6c**, showing the chromatographic signals, retention times, peak areas, and experimentally determined elemental composition.
